# Cesium Carbonate-Catalyzed Oxidative *Cross*-Dehydrogenative Thiolation of Phosphonothioates

**DOI:** 10.1021/acs.joc.4c02718

**Published:** 2025-02-06

**Authors:** Hsiu-Te Hung, Rekha Bai, Sung-Hung Lee, Indrajit Karmakar, Chin-Fa Lee

**Affiliations:** † Department of Chemistry, National Chung Hsing University, Taichung-402202, Taiwan 402, Republic of China; ‡ i-Center for Advanced Science and Technology (iCAST), National Chung Hsing University, Taichung-402202, Taiwan 402, Republic of China; § Innovation and Development Center of Sustainable Agriculture (IDCSA), National Chung Hsing University, Taichung-402202, Taiwan 402, Republic of China

## Abstract

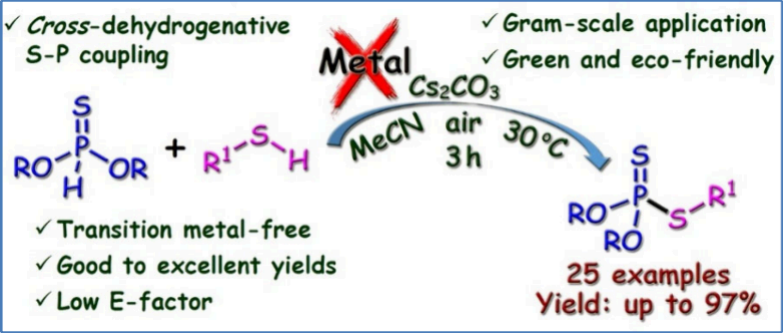

Herein, we report an aerobic oxidative *cross*-dehydrogenative coupling (CDC) reaction between thiols and phosphonothioates. The reactions were conducted under mild and transition-metal-free conditions in the presence of air. Not only aryl thiols but also alkyl thiols were successfully coupled with phosphonothioates to form the corresponding phosphorodithioates in good to excellent yields.

## 1 Introduction

**Figure 1:**
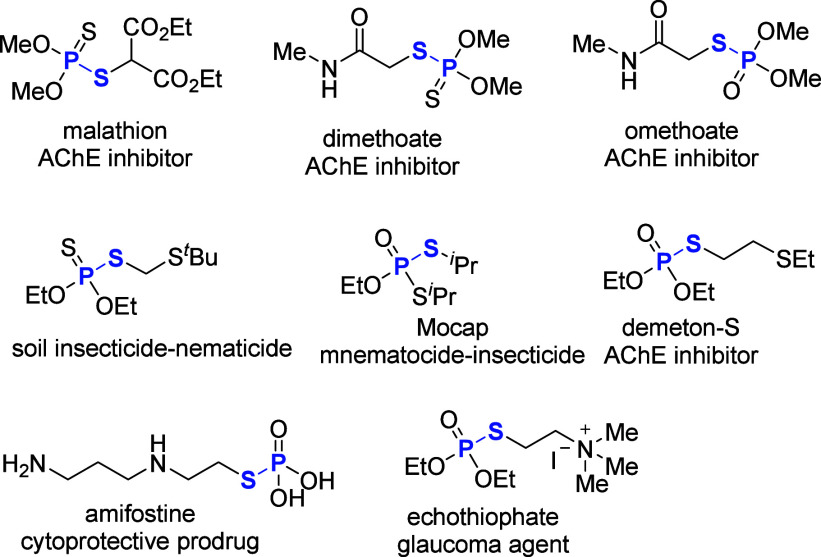
Representative biologically active thiophosphates and dithiophosphates.

**Scheme 1:**
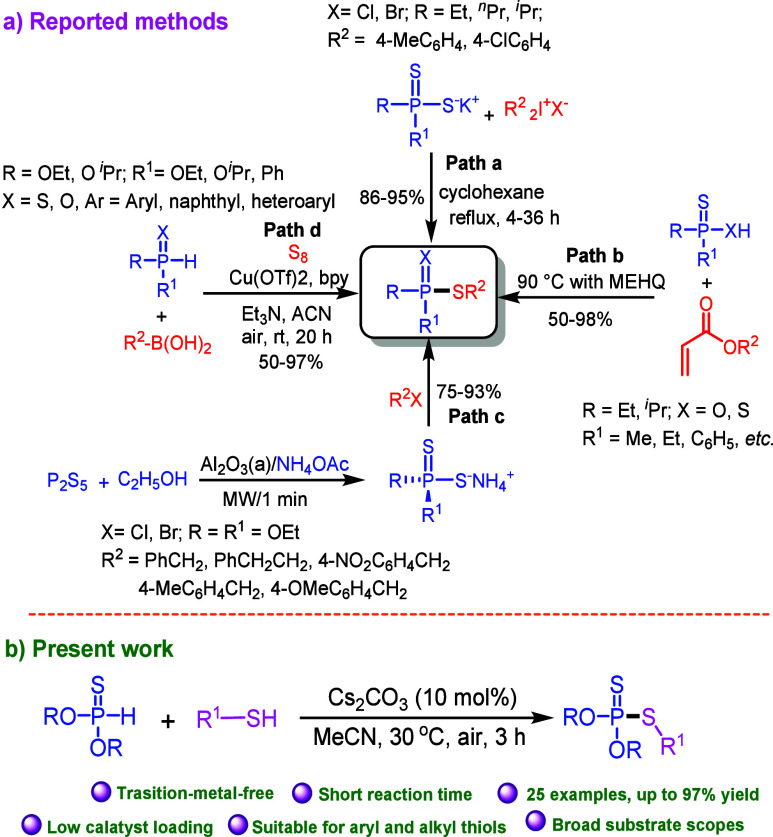
(a) Reported Method for the Synthesis of Phosphorodithioates, (b) Present Work

Phosphate esters are crucial in organic synthesis as they are present in biologically active moieties and serve as versatile intermediates in amide synthesis.^1−8^ Phosphorodithioates are well-known for their applications as antiviral agents,^9−11^ plant growth regulators,^12^ enzyme inhibitors,^13^ and lubricants^14^ (Figure 1). Additionally, several phosphorodithioates have been introduced as thionating agents.^15−19^ Consequently, the preparation of phosphorodithioates has garnered significant attention due to their diverse applications.^20−32^ Liu et al. prepared *O*,*O*-dialkyl *S*-aryl phosphorothiolates by reacting diaryliodonium salt with potassium *O*,*O*-dialkyl phosphorothiolates in cyclohexane under reflux conditions (Scheme 1a, Path a).^29^ A Michael addition reaction of *O*,*O*-dialkylthiophosphoric acid and *O*,*O*-dialkyldithiophosphoric acids with various acrylates for the synthesis of β-carboxylated thiolophosphates was developed by Oget and co-workers (Scheme 1a, Path b).^30^ Kaboudin and Norouzi have developed an alumina-supported and microwave-assisted reaction for the preparation of phosphorodithioates and thiiranes in one step by using phosphorus pentasulfides under solvent-free conditions (Scheme 1a, Path c).^31^ Tang and co-workers successfully developed a multistep reaction for the preparation of *S*-aryl phosphorothioates via phosphorothiolation of boronic acids, P(S)H compounds, and sulfur powder (Scheme 1a, Path d).^32^ However, these systems have certain drawbacks, such as the use of toxic reagents, harsh reaction conditions, narrow substrate scopes, and the involvement of air-sensitive reagents. Therefore, the development of milder, more environmentally friendly methods for the synthesis of phosphorothioates is highly desirable.

*Cross*-dehydrogenative coupling (CDC) reactions have gained significant attention for their ability to enhance reaction efficiency and improve atom economy.^33−38^ However, the oxidative CDC of thiols and phosphonates to form P–S bond remains challenging because the P–H and S–H bonds are readily oxidized by stoichiometric oxidants.^39−41^ Molecular oxygen (O_2_), a green and ideal oxidant, is widely used in organic synthesis.^42−52^ Here, we present a simple Cs_2_CO_3_-catalyzed aerobic oxidative *cross*-dehydrogenative coupling between thiols and phosphonates for the synthesis of dithiophosphates. The system, characterized by mild reaction conditions, good functional group tolerance, and broad S–H and P(S)-H scopes, provides a valuable protocol for organic synthesis (Scheme 1b).

## 2 Results and Discussion

Initially, *O*,*O*-diethyl phosphonothioate (**1a**, 0.5 mmol) and thiophenol (**2a**, 0.6 mmol) were selected as the substrates to optimize the reaction conditions. The results are summarized in Table 1. Product **3a** was obtained in 95% yield when the reaction was conducted in the presence of 10 mol % of Cs_2_CO_3_ in acetonitrile at 30 °C for 3 h under open air (Table 1, entry 1). A Lower amount of Cs_2_CO_3_ diminished the product yield to 80% (Table 1, entry 2). An 89% yield was observed when Cs_2_CO_3_ increased to 15 mol % (Table 1, entry 3). Other alkali-metal bases such as Li_2_CO_3_ and Na_2_CO_3_ showed no reactivity in this system (Table 1, entries 4 and 5). A 59% yield of the target was obtained when K_2_CO_3_ was used as a base (Table 1, entry 6). Testing other cesium salts, including CsOH, CsOAc, and CsOPiv, showed that these bases were less reactive than Cs_2_CO_3_ to give the product in 45–75% yields (Table 1, entries 7–9). A lower yield was observed when the reaction was performed at 50 °C (Table 1, entry 10). Solvents were examined (Table 1, entries 11–15). When DMSO was used as the solvent, only 15% of the product was isolated (Table 1, entry 11). Other solvents such as EA, toluene, DCM, EtOH showed poor results. Only trace amounts of the product were detected by GC-MS analysis when the reaction was carried out under neat conditions (Table 1, entry 16). A 95% yield was obtained when the reaction was performed under an oxygen atmosphere (Table 1, entry 17). Trace amount of the product was detected when the reaction was reacted under nitrogen (Table 1, entry 18). Both shorter and longer reaction times led to decreased product yields (Table 1, entries 19 and 20, respectively). Based on these results, we decided to carry out the reactions by using Cs_2_CO_3_ (10 mol %) and MeCN under air as the optimized reaction conditions.

**Table 1:**
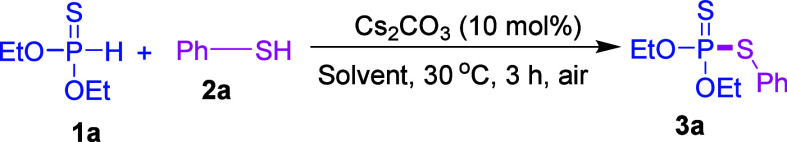
Optimization of the Reaction Conditions^a^

Entry	Base	Solvent	Yield of **3a** (%)^b^
**1**	**Cs**_**2**_**CO**_**3**_	**MeCN**	**95**
2^c^	Cs_2_CO_3_	MeCN	80
3^d^	Cs_2_CO_3_	MeCN	89
4	Li_2_CO_3_	MeCN	NR
5	Na_2_CO_3_	MeCN	NR
6	K_2_CO_3_	MeCN	59
7	CsOH	MeCN	45
8	CsOAc	MeCN	68
9	CsOPiv	MeCN	75
10^e^	Cs_2_CO_3_	MeCN	71
11	Cs_2_CO_3_	DMSO	15
12	Cs_2_CO_3_	EA	Trace
13	Cs_2_CO_3_	Toluene	NR
14	Cs_2_CO_3_	DCM	NR
15	Cs_2_CO_3_	EtOH	Trace
16	Cs_2_CO_3_	Neat	Trace
17^f^	Cs_2_CO_3_	MeCN	95
18^g^	Cs_2_CO_3_	MeCN	Trace
19^h^	Cs_2_CO_3_	MeCN	48
20^i^	Cs_2_CO_3_	MeCN	80

a All reaction were carried out with **1a** (0.5 mmol), **2a** (0.6 mmol), base (10 mol %) in solvent (2 mL) for 3 h under air.

b Isolated yields.

c Cs_2_CO_3_ used 7 mol %.

d Cs_2_CO_3_ used 15 mol %.

e 50 °C.

f O_2_.

g N_2_.

h 1 h.

i 5 h. MeCN = acetonitrile, DMSO = dimethyl sulfoxide, EA = ethyl acetate, DCM = dichloromethane, EtOH = ethyl alcohol.

**Scheme 2:**
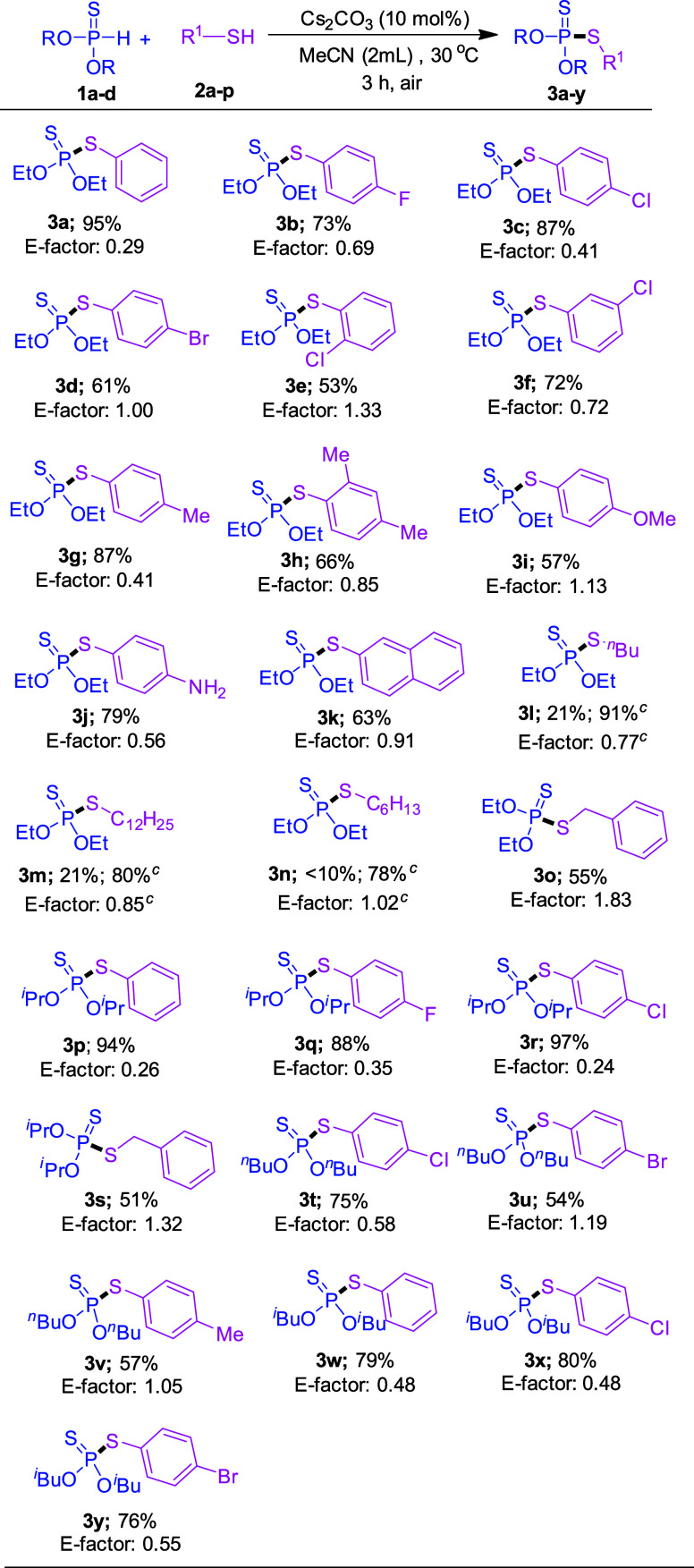
Substrate Scope of Thiols and Phosphonothioates^,^ All reaction carried out with **1** (0.5 mmol), **2** (0.6 mmol), and Cs_2_CO_3_ (10 mol %) in MeCN (2 mL) for 3 h under air. Isolated yields of **3a**–**3y**. Cs_2_CO_3_ (40 mol %) was used.

With the optimized reaction conditions in hand, we then explored the scope of this P–S *cross*-coupling reaction (Scheme 2). Thiophenols with halogen atoms such as F, Cl and Br at the *para*-position on the aromatic ring reacted with *O,O*-diethyl phosphonothioate (**1a**), affording the corresponding products **3b**, **3c**, and **3d** in 73%, 87%, and 61% yields, respectively. Thiophenols with chloro groups at *ortho*- and *meta*- positions were also coupled with *O,O*-diethyl phosphonothioate to give phosphorodithioates **3e** and **3f** in 53% and 72% yields, respectively. 4-Methylthiophenol (**2g**) and sterically hindered 2,4-dimethylbenzenethiol (**2h**) were well-tolerated in the reaction conditions, providing the corresponding phosphorodithioates **3g** and **3h** in 87% and 66% yields, respectively. Thiophenols with an electron-donating group and electron-withdrawing group at the *para*-position of the aromatic ring such as -OMe and -NH_2_ afforded the corresponding products **3i**–**3j** in 57–79% yields.

Additionally, naphthalene-2-thiol (**2k**) was also reacted with **1a** to afford *O,O*-diethyl *S*-(naphthalen-2-yl) phosphorodithioate (**3k**) in 63% yield. Alkyl thiols such as 1-butanethiol (**2l**), 1-dodecanethiol (**2m**), hexane-1-thiol (**2n**), and benzyl thiol (**2o**) were also reacted with **1a**, providing phosphorodithioates **3l**–**3o** in 55–91% yields. Next, we extended our methodology to different phosphonothioates such as (O^*i*^Pr)_2_P(S)H, (O^*n*^Bu)_2_P(S)H and (O^*i*^Bu)_2_P(S)H. *O,O*-Diisopropyl phosphonothioate (**1b**) was reacted with thiophenol (**2a**), 4-fluorothiophenol (**2b**), 4-chlorothiophenol (**2c**), and benzyl thiol (**2o**) under the standard reaction conditions, affording the corresponding P–S coupled products **3p**–**3s** in 51–97% yields. *O,O*-Dibutyl phosphonothioate (**1c**) reacted with 4-chlorothiophenol, 4-bromothiophenol, and 4-methylthiophenol, and provided the desired products **3t**–**3v** in good yields.

Moreover, the sterically hindered *O,O*-diisobutyl phosphonothioate was also successfully coupled with thiophenol (**2a**), 4-chlorothiophenol (**2c**) and 4-bromothiophenol (**2d**) giving the corresponding products **3w**–**3y** in 76–80% yields.

**Scheme 3:**
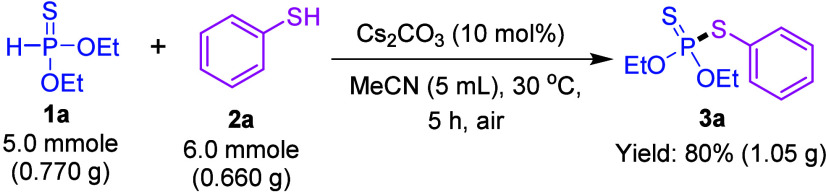
Gram-Scale Experiment

We also tested the scalability of this CDC reaction on a larger scale (5.0 mmol, gram scale) by using our model reaction. The large-scale reaction yielded the target product, *O*,*O*-Diethyl *S*-phenyl phosphorodithioate (**3a**) was afforded in 80% (1.05 g) yield after 5 h (Scheme 3). Notably, the large-scale reaction exhibited results almost comparable to those on the 0.5 mmol scale in terms of both yield and reaction time.

To assess the environmental sustainability of this newly developed method, we calculated the E-factor (g/g) for all the synthesized compounds (**3a**–**3y**), as it is a key green chemistry metric.^53,54^ The calculated E-factors (g/g) range from 0.24 to 1.83, indicating the significant environmental sustainability of the method. Water is the only green byproducts in this transformation.

**Scheme 4:**
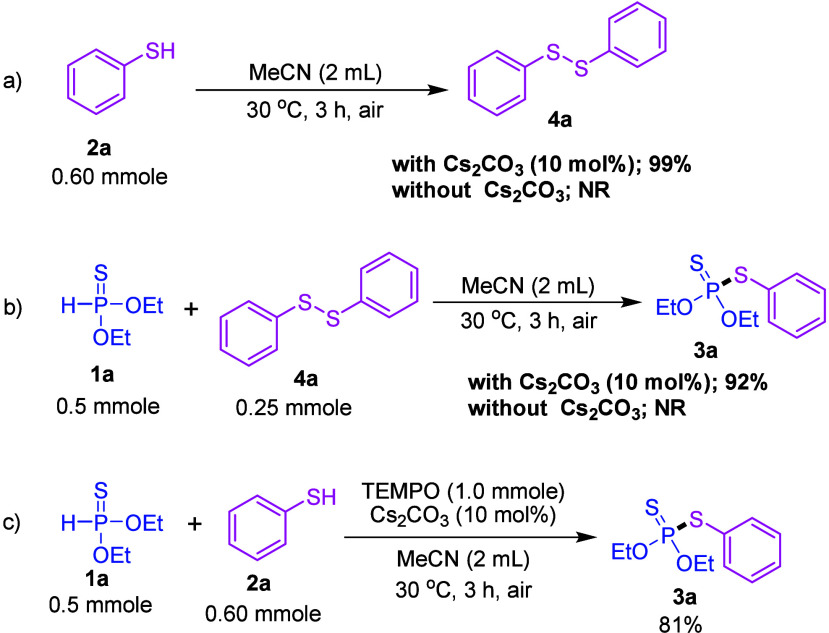
Control Experiments

To elucidate the mechanism of this P–S *cross*-dehydrogenative coupling reaction, we performed control experiments to investigate the roles of Cs_2_CO_3_ and air. When thiophenol (**2a**) was subjected in the optimized reaction conditions, the phenyl disulfide (**4a**) was obtained with a 99% isolated yield (Scheme 4a). This result confirmed that Cs_2_CO_3_ and air are essential for the formation of phenyl disulfide in this CDC reaction (Scheme 4a). We then tested the reaction between *O,O*-diethyl phosphonothioate (**1a**) and phenyl disulfide (**4a**) under the same optimized conditions, resulting in the formation of *O,O*-diethyl *S*-phenyl phosphorodithioate (**3a**) in 92% yield (Scheme 4b). No product was formed in the absence of Cs_2_CO_3_ (Scheme 4b). Additionally, when the reaction was conducted in the presence of 2 equiv of TEMPO, the corresponding P–S coupled product **3a** in 81% yield (Scheme 4c). With no TEMPO-containing product detected. This result suggested the reaction mechanism does not involve a radical pathway.

**Scheme 5:**
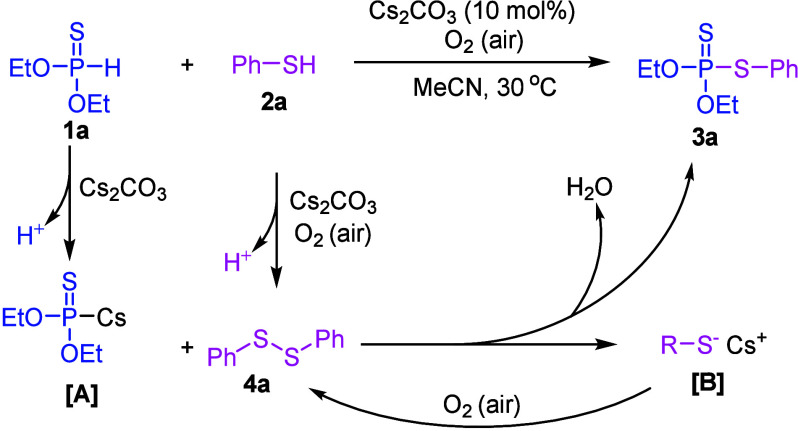
Plausible Mechanism

Based on the control experiments, the mechanism for this reaction is proposed in Scheme 5. Initially, *O*,*O*-diethyl phosphonothioate (**1a**) reacts with Cs_2_CO_3_ to form the intermediate [**A**]. Simultaneously, phenyl disulfide (**4a**) is generated in the presence of Cs_2_CO_3_ and oxygen (from air), as confirmed by the control experiment (Scheme 4a). Intermediate [**A**] undergoes a reaction with disulfide **4a**, leading to the formation of final product **3a**, along with intermediate [**B**] and the release of one molecule of water. Intermediate [**B**] is subsequently oxidized back to disulfide **4a** by O_2_ (air).^55^

## 3 Conclusions

In summary, we have developed a Cs_2_CO_3_-catalyzed *cross*-dehydrogenative coupling reaction between thiols and phosphonothioates to afford phosphorodithioates. The main advantages of this method are the absence of toxic transition metal catalysts, air as an oxidant, its operational simplicity, good to excellent yields, demonstrates good functional group tolerance, scalability to the gram level, and low E-factor, all of which align with the principles of green and sustainable chemistry

## 4 Experimental Section

### General Information

All of the reagents and chemicals were purchased at the highest quality from commercial suppliers and used without further purification. Yields refer to chromatographically and spectroscopically (^1^H NMR) homogeneous material, unless otherwise stated. Reactions were monitored by GC/MS and thin-layer chromatography (TLC). TLC was performed using 0.25 mm E. Merck silica plates (60F_254_), using short-wave UV light as the visualizing agent or KMnO_4_ and heat as developing agents. ^1^H NMR, ^13^C NMR, ^19^F NMR, ^31^P NMR, and HRMS techniques were used for the analysis of the synthesized compounds. Chemical shifts reported in parts per million (ppm) with reference to the TMS at 0.00 ppm for ^1^H NMR and coupling constants (*J*) are given in Hz. ^1^H NMR peak signals were reported as s (singlet), br (broad), d (doublet), dd (double doublet), td (triplet of doublets), ddd (doublet of double doublets), qd (quartet of doublets), quint (pentet), sept (septet), and m (multiplet). In the ^13^C NMR, chemical shifts were reported in ppm by referencing the center line of a triplet of chloroform-d at 77.10 ppm. High-resolution mass spectra (HRMS) were recorded on a Jeol JMS-HX 110 quadrupole type spectrometer provided by the National Chung Hsing University. GC-MS analyses were recorded on Agilent Technologies 5977A GC equipped with Agilent 7890B MS. All melting points (M.P.) were observed by using a Büchi 535 apparatus. Column chromatography was performed over silica-gel (particle size: 100–200 Mesh) using hexanes and ethyl acetate as eluent. Starting materials **1a**-**1d**^25^ were synthesized using reported literature.

### General Procedure for Table 1

To a reaction tube were added *O,O*-diethyl phosphonothioate (**1a**, 0.5 mmol, 0.077g), benzenethiol (2a, 0.6 mmol, 0.066 g), and base (7.0 mol % to 15 mol %) in solvent. The resulting solution was stirred at 30 °C in an oil bath for 1–5 h under gas. After the completion of the reaction, the solvent was evaporated and the crude was diluted with water (20 mL) followed by extracted with ethyl acetate (3 × 20 mL). The combined organic layers were concentrated under reduced pressure to get crude products, which were further purified through column chromatography using ethyl acetate/hexanes (1:9) as an eluent to afford the corresponding products **3a**.

### *O,O*-Diethyl *S*-Phenyl Phosphorodithioate (3a)^25^

The title compound was prepared following the general procedure for Table 1; *O,O*-diethyl phosphonothioate (**1a**, 0.5 mmol, 0.077 g), benzenethiol (**2a**, 0.6 mmol, 0.066 g), and Cs_2_CO_3_ (10 mol %, 0.016 g), after column chromatography (10–15% EtOAc/Hexanes) obtained **3a** as a colorless oil; Yield: 0.125 g, 95%. ^1^H NMR (400 MHz, chloroform-*d*): δ 7.46–7.43 (m, 2H), 7.32–7.19 (m, 3H), 4.23–4.05 (m, 4H), 1.24 (td, *J* = 8.0 and 0.8 Hz, 6H); ^13^C{H} NMR (100 MHz, chloroform-*d*): δ 134.7 (d, *J* = 5.0 Hz), 129.3 (d, *J* = 2.0 Hz), 128.3 (d, *J* = 8.0 Hz), 64.2 (d, *J* = 6.0 Hz), 15.7 (d, *J* = 8.0 Hz); ^31^P NMR (162 MHz, chloroform-*d*) δ 88.66.

### General Procedure for Scheme 2

To a reaction tube were added phosphonate derivatives (0.5 mmol), thiols (0.6 mmol) and Cs_2_CO_3_ (10 mol %) in CH_3_CN solvent. The resulting solution was stirred at 30 ^ο^C in an oil bath for 3 h under air. After the completion of the reaction, the acetonitrile solvent was evaporated and the crude was diluted with water (20 mL) followed by extracted with ethyl acetate (3 × 20 mL). The combined organic layers were concentrated under reduced pressure to get crude products, which were further purified through column chromatography using ethyl acetate/hexanes as an eluent to afford the corresponding products **3b**–**3y**

### General Procedure for Gram-Scale Synthesis

To a reaction tube, *O*,*O*-diethyl phosphonothioate (**1a**, 5.0 mmol, 0.770 g), benzenethiol (**2a**, 6.0 mmol, 0.660 g), and Cs_2_CO_3_ (10 mol %) were added in 5 mL of acetonitrile (CH_3_CN) solvent. The resulting solution was stirred at 30 °C in an oil bath for 5 h under air. Upon completion of the reaction, the acetonitrile solvent was evaporated and the residue was diluted with water (50 mL), followed by extraction with ethyl acetate (3 × 50 mL). The combined organic layers were concentrated under reduced pressure to yield the crude product, which was then purified by column chromatography, using ethyl acetate/hexanes as the eluent. The corresponding product, *O*,*O*-diethyl *S*-phenyl phosphorodithioate (**3a**), was obtained in 80% yield (1.05 g).

### *O,O*-Diethyl *S*-(4-fluorophenyl) phosphorodithioate (3b)^25^

The title compound was prepared following the general procedure for Scheme 2; *O,O*-diethyl phosphonothioate (**1a**, 0.5 mmol, 0.077 g), 4-fluorobenzenethiol (**2b**, 0.6 mmol, 0.077 g), Cs_2_CO_3_ (10 mol %, 0.016 g), after column chromatography (10–15% EtOAc/Hexanes), obtained **3b** as a colorless oil; Yield: 0.102 g, 73% yield. ^1^H NMR (400 MHz, chloroform-*d*): δ 7.52–7.48 (m, 2H), 7.06 (td, *J* = 8.4 and 0.8 Hz, 2H), 4.29–4.11 (m, 4H), 1.32 (td, *J* = 6.8 and 0.8 Hz, 6H); ^13^C{H} NMR (100 MHz, chloroform-*d*): δ 163.55 (dd, *J* = 253 and 4 Hz), 136.7 (q, *J* = 5.0 Hz), 123.5 (q, *J* = 5.0 Hz), 116.6 (d, *J* = 3.0 Hz), 116.4 (d, *J* = 3.0 Hz), 64.4 (d, *J* = 6.0 Hz), 15.8 (d, *J* = 9.0 Hz); ^31^P NMR (162 MHz, chloroform-*d*) δ 88.81 (d, *J* = 6.2 Hz); ^19^F NMR (376 MHz, CDCl_3_) δ −110.93.

### *S*-(4-Chlorophenyl) *O,O*-diethyl phosphorodithioate (3c)^56^

The title compound was prepared following the general procedure for Scheme 2; *O,O*-diethyl phosphonothioate (**1a**, 0.5 mmol, 0.077 g), 4-chlorobenzenethiol (**2c**, 0.6 mmol, 0.087 g), and Cs_2_CO_3_ (10 mol %, 0.016 g), after column chromatography (10–15% EtOAc/Hexanes) obtained **3c** as a colorless oil; Yield: 0.129 g, 87% yield. ^1^H NMR (400 MHz, chloroform-*d*): δ 7.45 (dd, *J* = 8.4 and 2.0 Hz, 2H), 7.34 (d, *J* = 8.4 Hz, 2H), 4.28–4.13 (m, 4H), 1.32 (dd, *J* = 8.4 and 1.2 Hz, 6H); ^13^C{H} NMR (100 MHz, chloroform-*d*): δ 136.0 (d, *J* = 5.0 Hz), 135.8 (d, *J* = 4.0 Hz), 129.5 (d, *J* = 3.0 Hz), 126.8 (d, *J* = 8.0 Hz), 64.4 (d, *J* = 6.0 Hz), 15.8 (d, *J* = 8.0 Hz); ^31^P NMR (162 MHz, chloroform-*d*) δ 88.06. HRMS (EI), calcd for C_10_H_14_ClO_2_PS_2_ [M]^+^ 295.9861, found 295.9863.

### *S*-(4-Bromophenyl) *O,O*-diethyl phosphorodithioate (3d)

The title compound was prepared following the general procedure for Scheme 2; *O,O*-diethyl phosphonothioate (**1a**, 0.5 mmol, 0.077 g), 4-bromobenzenethiol (**2d**, 0.6 mmol, 0.113 g), Cs_2_CO_3_ (10 mol %, 0.016 g), after column chromatography (10–15% EtOAc/Hexanes) obtained **3d** as a colorless oil; Yield: 0.104 g, 61% yield. ^1^H NMR (400 MHz, chloroform-*d*) δ 7.51–7.47 (m, 2H), 7.39–7.26 (m, 2H), 4.29–4.11 (m, 4H), 1.32 (td, *J* = 7.2 and 0.8 Hz, 6H); ^13^C{H} NMR (100 MHz, chloroform-*d*): δ 136.2 (d, *J* = 5.0 Hz), 132.5 (d, *J* = 3.0 Hz), 127.5 (d, *J* = 7.0 Hz), 124.0 (d, *J* = 4.0 Hz), 64.4 (d, *J* = 6.0 Hz), 15.8 (d, *J* = 8.0 Hz); ^31^P NMR (162 MHz, chloroform-*d*): δ 87.79. HRMS (EI), calcd for C_10_H_14_BrO_2_PS_2_ [M]^+^ 339.9356, found 339.9354.

### *S*-(2-Chlorophenyl) *O,O*-diethyl phosphorodithioate (3e)

The title compound was prepared following the general procedure for Scheme 2; *O,O*-diethyl phosphonothioate (**1a**, 0.5 mmol, 0.077 g), 2-chlorobenzenethiol (**2e**, 0.6 mmol, 0.087 g), Cs_2_CO_3_ (10 mol %, 0.016 g), after column chromatography (10–15% EtOAc/Hexanes) obtained **3e** as a colorless oil; Yield: 0.078 g, 53% yield. ^1^H NMR (400 MHz, chloroform-*d*): δ 7.69 (dt, *J* = 8.0 and 2.4 Hz, 1H), 7.47 (dt, *J* = 7.6 and 0.8 Hz, 1H), 7.34–7.29 (m, 1H), 7.28–7.24 (m, 1H), 4.33–4.17 (m, 4H), 1.32 (td, *J* = 7.2 and 0.8 Hz, 6H); ^13^C{H} NMR (100 MHz, chloroform-*d*): δ 138.1 (d, *J* = 6.0 Hz), 137.3 (d, *J* = 4.0 Hz), 130.7 (d, *J* = 3.0 Hz), 130.3 (d, *J* = 3.0 Hz), 128.0 (d, *J* = 9.0 Hz), 127.4 (d, *J* = 3.0 Hz), 64.5 (d, *J* = 5.0 Hz), 15.8 (d, *J* = 11.0 Hz); ^31^P NMR (162 MHz, chloroform-*d*): δ 87.36. HRMS (EI), calcd for C_10_H_14_ClO_2_PS_2_ [M]^+^ 295.9861, found, 295.9867.

### *S*-(3-Chlorophenyl) *O,O*-diethyl phosphorodithioate (3f)^57^

The title compound was prepared following the general procedure for Scheme 2; *O,O*-diethyl phosphonothioate (**1a**, 0.5 mmol, 0.077 g), 3-chlorobenzenethiol (**2f**, 0.6 mmol, 0.087 g), and Cs_2_CO_3_ (10 mol %, 0.016 g), after column chromatography (10–15% EtOAc/Hexanes) obtained **3f** as a colorless oil; Yield: 0.106 g, 72% yield. ^1^H NMR (400 MHz, chloroform-*d*): δ 7.51 (q, *J* = 2.0 Hz, 1H), 7.42–7.39 (m, 1H), 7.38–7.34 (m, 1H), 7.31–7.26 (m,1H), 4.30–4.13 (m, 4H), 1.33 (t, *J* = 7.2 and 0.8 Hz, 6H); ^13^C{H} NMR (100 MHz, chloroform-*d*): δ 134.7 (d, *J* = 2.0 Hz), 134.5 (d, *J* = 4.0 Hz), 132.8 (d, *J* = 4.0 Hz), 130.2 (d, *J* = 10.0 Hz), 130.1 (d, *J* = 7.0 Hz), 129.5 (d, *J* = 3.0 Hz), 64.5 (d, *J* = 6.0 Hz), 15.8 (d, *J* = 8.0 Hz); ^31^P NMR (162 MHz, chloroform-*d*): δ 87.41. HRMS (EI), calcd for C_10_H_14_ClO_2_PS_2_ [M]+ 295.9861, found 295.9867.

### *O,O*-Diethyl S-(*p*-tolyl) phosphorodithioate (3g)^25^

The title compound was prepared following the general procedure for Scheme 2; *O,O*-diethyl phosphonothioate (**1a**, 0.5 mmol, 0.077 g), 4-methylbenzenethiol (**2g**, 0.6 mmol, 0.075 g), Cs_2_CO_3_ (10 mol %, 0.016 g), after column chromatography (15–20% EtOAc/Hexanes) obtained **3g** as a colorless oil; Yield: 0.120 g, 87% yield. ^1^H NMR (400 MHz, chloroform-*d*): δ 7.39 (dt, *J* = 4.4 and 2.0 Hz, 2H), 7.18–7.15 (m, 2H), 4.27–4.14 (m, 4H), 2.35 (d, *J* = 2.4 Hz, 3H), 1.32 (td, *J* = 7.2 and 0.8 Hz, 6H); ^13^C{H} NMR (100 MHz, chloroform-*d*): δ 139.7 (d, *J* = 2.0 Hz), 134.8 (d, *J* = 5.0 Hz), 130.1 (d, *J* = 2.0 Hz), 124.6 (d, *J* = 8.0 Hz), 64.1 (d, *J* = 5.0 Hz), 21.3, 15.8 (d, *J* = 9.0 Hz); ^31^P NMR (162 MHz, chloroform-*d*): δ 89.28.

### *S*-(2,4-Dimethylphenyl) *O,O*-diethyl phosphorodithioate (3h)

The title compound was prepared following the general procedure for Scheme 2; *O,O*-diethyl phosphonothioate (**1a**, 0.5 mmol, 0.077 g), 2,4-dimethylbenzenethiol (**2h**, 0.6 mmol, 0.083 g), Cs_2_CO_3_ (10 mol %, 0.016 g), after column chromatography (15–20% EtOAc/Hexanes) obtained **3h** as a colorless oil; Yield: 0.096 g, 66% yield. ^1^H NMR (400 MHz, chloroform-*d*): δ 7.40 (dd, *J* = 8.0 and 2.4 Hz, 1H), 7.08 (d, *J* = 2.0 Hz, 1H), 6.99 (dd, *J* = 8.0 and 2.0 Hz, 1H), 4.26–4.09 (m, 4H), 2.46 (d, *J* = 1.2 Hz, 3H), 2.31 (d, *J* = 2.8 Hz, 3H), 1.31 (td, *J* = 7.2 and 0.8 Hz, 6H); ^13^C{H} NMR (100 MHz, chloroform-*d*): δ 142.0 (d, *J* = 5.0 Hz), 139.9 (d, *J* = 4.0 Hz), 136.4 (d, *J* = 4.0 Hz), 131.7 (d, *J* = 3.0 Hz), 127.5 (d, *J* = 3.0 Hz), 123.9 (d, *J* = 8.0 Hz), 64.2 (d, *J* = 7.0 Hz), 21.3, 21.2, 15.8 (d, *J* = 8.0 Hz); ^31^P NMR (162 MHz, chloroform-*d*): δ 90.51. HRMS (EI), calcd for C_12_H_19_O_2_PS_2_ [M]^+^ 290.0564, found 290.0560.

### *O,O*-Diethyl *S*-(4-methoxyphenyl) phosphorodithioate (3i)^57^

The title compound was prepared following the general procedure for Scheme 2; *O,O*-diethyl phosphonothioate (**1a**, 0.5 mmol, 0.077 g), 4-methoxybenzenethiol (**2i**, 0.6 mmol, 0.084 g), Cs_2_CO_3_ (10 mol %, 0.016 g), after column chromatography (25–30% EtOAc/Hexanes) obtained **3i** as a colorless oil; Yield: 0.084 g, 57% yield. ^1^H NMR (400 MHz, chloroform-*d*): δ 7.43–7.40 (m, 2H), 6.89–6.87 (m, 2H), 4.27–4.13 (m, 4H), 3.81 (d, *J* = 1.2 Hz, 3H), 1.31 (td, *J* = 7.2 and 1.2 Hz, 6H); ^13^C{H} NMR (100 MHz, chloroform-*d*): δ 160.7 (d, *J* = 1.0 Hz), 136.6 (d, *J* = 4.0 Hz), 118.6 (d, *J* = 9.0 Hz), 114.9 (d, *J* = 2.0 Hz), 64.1 (d, *J* = 5.0 Hz), 55.4, 15.8 (d, *J* = 9.0 Hz); ^31^P NMR (162 MHz, chloroform-*d*): δ 89.67.

### *S*-(4-Aminophenyl) *O,O*-diethyl phosphorodithioate (3j)^25^

The title compound was prepared following the general procedure for Scheme 2; *O,O*-diethyl phosphonothioate (**1a**, 0.5 mmol, 0.077 g), 4-aminobenzenethiol (**2j**, 0.6 mmol, 0.075 g), Cs_2_CO_3_ (10 mol %, 0.016 g), after column chromatography (25–30% EtOAc/Hexanes) obtained **3j** as a colorless oil; Yield: 0.109 g, 79% yield. ^1^H NMR (400 MHz, chloroform-*d*): δ 7.28–7.24 (m, 2H), 6.63 (d, *J* = 8.4 Hz, 2H), 4.27–4.09 (m, 4H), 3.82 (s, 2H), 1.31 (td, *J* = 7.2 and 0.8 Hz, 6H); ^13^C{H} NMR (100 MHz, chloroform-*d*): δ 147.8, 136.5 (d, *J* = 4.0 Hz), 134.0, 115.5 (d, *J* = 3.0 Hz), 64.0 (d, *J* = 6.0 Hz), 15.8 (d, *J* = 9.0 Hz); ^31^P NMR (162 MHz, chloroform-*d*): δ 90.41. HRMS (EI), calcd for C_10_H_16_NO_2_PS_2_ [M]^+^ 277.0360, found 277.0356.

### *O,O*-Diethyl *S*-(naphthalen-2-yl) phosphorodithioate (3k)

The title compound was prepared following the general procedure for Scheme 2; *O,O*-diethyl phosphonothioate (**1a**, 0.5 mmol, 0.077 g), naphthalene-2-thiol (**2k**, 0.6 mmol, 0.096 g), and Cs_2_CO_3_ (10 mol %, 0.016 g), after column chromatography (20–25% EtOAc/Hexanes) obtained **3k** as a colorless oil; Yield: 0.099 g, 63% yield. ^1^H NMR (400 MHz, chloroform-*d*): δ 8.03 (t, *J* = 2.8 Hz, 1H), 7.87–7.81 (m, 3H), 7.57–7.51 (m, 3H), 4.32–4.16 (m, 4H), 1.32 (td, *J* = 7.2 and 0.8 Hz, 6H); ^13^C{H} NMR (100 MHz, chloroform-*d*): δ 134.8 (d, *J* = 6.0 Hz), 133.6 (d, *J* = 6.0 Hz), 133.2 (d, *J* = 3.0 Hz), 131.1 (d, *J* = 4.0 Hz), 128.9 (d, *J* = 3.0 Hz), 127.9 (d, *J* = 6.0 Hz), 127.2, 126.8, 125.5 (d, *J* = 8.0 Hz), 64.3 (d, *J* = 6.0 Hz), 15.8 (d, *J* = 8.0 Hz); ^31^P NMR (162 MHz, chloroform-*d*): δ 88.64. HRMS (EI), calcd for C_14_H_17_O_2_PS_2_ [M]^+^ 312.0408, found 312.0399.

### *S*-Butyl *O,O*-diethyl phosphorodithioate (3l)^25^

The title compound was prepared following the general procedure for Scheme 2; *O,O*-diethyl phosphonothioate (**1a**, 0.5 mmol, 0.077 g), butane-1-thiol (**2l**, 0.6 mmol, 0.054 g), and Cs_2_CO_3_ (10 mol %, 0.016 g), after column chromatography (20–25% EtOAc/Hexanes) obtained **3l** as a colorless oil; Yield: 0.110 g, 91% yield. ^1^H NMR (400 MHz, chloroform-*d*): δ 4.26–4.08 (m, 4H), 2.90–2.83 (m, 2H), 1.68–1.61 (m, 2H), 1.41–1.34 (m, 8H), 0.93 (t, *J* = 7.2 Hz, 3H); ^13^C{H} NMR (100 MHz, chloroform-*d*): δ 63.8 (d, *J* = 6.0 Hz), 33.3 (d, *J* = 4.0 Hz), 32.4 (d, *J* = 6.0 Hz), 21.8, 15.9 (d, *J* = 8.0 Hz), 13.6; ^31^P NMR (162 MHz, chloroform-*d*): δ 95.88. HRMS (EI), calcd for C_8_H_19_O_2_PS_2_ [M]^+^ 242.0564, found.

### *S*-Dodecyl *O,O*-diethyl phosphorodithioate (3m)

The title compound was prepared following the general procedure for Scheme 2; *O,O*-diethyl phosphonothioate (**1a**, 0.5 mmol, 0.077 g), dodecane-1-thiol ( **2m**, 0.6 mmol, 0.121 g), Cs_2_CO_3_ (10 mol %, 0.016 g), after column chromatography (20–25% EtOAc/Hexanes) obtained **3m** as a colorless oil; Yield: 0.142 g, 80% yield. ^1^H NMR (400 MHz, chloroform-*d*): δ 4.26–4.08 (m, 4H), 2.89–2.81 (m, 2H), 1.70–1.62 (m, 2H), 1.39–1.34 (m, 6H), 1.25 (s, 18H), 0.88 (t, *J* = 7.2 Hz, 3H); ^13^C{H} NMR (100 MHz, chloroform-*d*): δ 63.8 (d, *J* = 5.0 Hz), 33.6 (d, *J* = 4.0 Hz), 31.9, 30.4 (d, *J* = 5.0 Hz), 29.69, 29.62, 29.5, 29.4, 29.1, 28.7, 22.7, 15.9 (d, *J* = 9.0 Hz), 14.1; ^31^P NMR (162 MHz, chloroform-*d*): δ 95.89. HRMS (EI), calcd for C_16_H_35_O_2_PS_2_ [M]^+^ 354.1816, found 354.1823.

### *O,O*-Diethyl *S*-hexyl phosphorodithioate (3n)

The title compound was prepared following the general procedure for Scheme 2; *O,O*-diethyl phosphonothioate (**1a**, 0.5 mmol, 0.077 g), hexane-1-thiol (**2n**, 0.6 mmol, 0.071 g), Cs_2_CO_3_ (10 mol %, 0.016 g), after column chromatography (20–25% EtOAc/Hexanes) obtained **3n** as a colorless oil; Yield: 0.105 g, 78% yield. ^1^H NMR (400 MHz, chloroform-*d*): δ 4.24–4.09 (m, 4H), 2.89–2.81 (m, 2H), 1.68–1.62 (m, 2H), 1.38–1.34 (m, 8H), 1.31–1.25 (m, 4H), 0.90–0.87 (m, 3H); ^13^C{H} NMR (100 MHz, chloroform-*d*): δ 63.9 (*d*, *J* = 6.0 Hz), 33.7 (d, *J* = 4.0 Hz), 31.3, 30.4 (d, *J* = 5.0 Hz), 28.4, 22.6, 15.9 (d, *J* = 8.0 Hz), 14.1; ^31^P NMR (162 MHz, chloroform-*d*): δ 95.85. HRMS (EI), calcd for C_10_H_23_O_2_PS_2_ [M+1]^+^ 271.0911, found 271.0945.

### *S*-Benzyl *O,O*-diethyl phosphorodithioate (3o)^31^

The title compound was prepared following the general procedure for Scheme 2; *O,O*-diethyl phosphonothioate (**1a**, 0.5 mmol, 0.077 g), phenyl methanethiol (**2o**, 0.6 mmol, 0.075 g), Cs_2_CO_3_ (10 mol %, 0.016 g), after column chromatography (10–15% EtOAc/Hexanes) obtained **3o** as a colorless oil; Yield: 0.076 g, 55% yield. ^1^H NMR (400 MHz, chloroform-*d*): δ 7.36–7.29 (m, 3H), 7.28–7.25 (m, 2H), 4.18–3.96 (m, 6H), 1.28 (t, *J* = 7.2 Hz, 6H); ^13^C{H} NMR (100 MHz, chloroform-*d*): δ 137.2, 129.0, 128.6, 127.6, 63.9 (d, *J* = 6.0 Hz), 37.6 (d, *J* = 4.0 Hz), 15.8 (d, *J* = 8.0 Hz); ^31^P NMR (162 MHz, chloroform-*d*): δ 93.90 HRMS (EI), calcd for C_11_H_17_O_2_PS_2_ [M]^+^ 276.0408, found 276.0399.

### *O,O*-Diisopropyl *S*-phenyl phosphorodithioate (3p)^25^

The title compound was prepared following the general procedure for Scheme 2; *O,O*-diisopropyl phosphonothioate (**1b**, 0.5 mmol, 0.091 g), benzenethiol (**2a**, 0.6 mmol, 0.066 g), and Cs_2_CO_3_ (10 mol %, 0.016 g), after column chromatography (10–15% EtOAc/Hexanes) obtained **3p** as a colorless oil; Yield: 0.137 g, 94% yield. ^1^H NMR (400 MHz, chloroform-*d*): δ 7.59–7.55 (m, 2H), 7.36–7.26 (m, 3H), 4.91–4.81 (m, 2H), 1.32 (dd, *J* = 6.4 and 2.0 Hz, 6H), 1.27 (dd, *J* = 6.0 and 2.0 Hz, 6H); ^13^C{H} NMR (100 MHz, chloroform-*d*): δ 134.6 (d, *J* = 5.0 Hz), 129.1 (d, *J* = 3.0 Hz), 129.0 (d, *J* = 3.0 Hz), 74.0 (d, *J* = 7.0 Hz), 23.8 (d, *J* = 4.0 Hz), 23.4 (d, *J* = 6.0 Hz); ^31^P NMR (162 MHz, chloroform-*d*): δ 86.56.

### *S*-(4-Fluorophenyl) *O,O*-diisopropyl phosphorodithioate (3q)

The title compound was prepared following the general procedure for Scheme 2; *O,O*-diisopropyl phosphonothioate (**1b**, 0.5 mmol, 0.091 g), 4-fluorobenzenethiol (**2b**, 0.6 mmol, 0.077 g), and Cs_2_CO_3_ (10 mol %, 0.016 g), after column chromatography (10–15% EtOAc/Hexanes) obtained **3q** as a colorless oil; Yield: 0.136 g, 88% yield. ^1^H NMR (400 MHz, chloroform-*d*): δ 7.57–7.53 (m, 2H), 7.08–7.03 (m, 2H), 4.91–4.79 (m, 2H), 1.32 (d, *J* = 6.0 Hz, 6H), 1.26 (d, *J* = 6.0 Hz, 6H); ^13^C{H} NMR (100 MHz, chloroform-*d*): δ 163.6 (d, *J* = 254 Hz), 136.9 (dd, *J* = 8.0 and 5.0 Hz), 124.1 (dd, *J* = 8.0 and 4.0 Hz), 116.2 (dd, *J* = 22.0 and 3.0 Hz), 74.1 (d, *J* = 8.0 Hz), 23.7 (d, *J* = 4.0 Hz), 23.4 (d, *J* = 4.0 Hz); ^31^P NMR (162 MHz, chloroform-*d*): δ 86.82(d, *J* = 6.1 Hz); ^19^F NMR (376 MHz, chloroform-*d*): δ −111.47··· HRMS (EI), calcd for C_12_H_18_FO_2_PS_2_ [M]^+^ 308.0470, found 308.0465

### *S*-(4-Chlorophenyl) *O,O*-diisopropyl phosphorodithioate (3r)^57^

The title compound was prepared following the general procedure for Scheme 2; *O,O*-diisopropyl phosphonothioate (**1b**, 0.5 mmol, 0.091 g), 4-chlorobenzenethiol (**2c**, 0.6 mmol, 0.087 g), and Cs_2_CO_3_ (10 mol %, 0.016 g), after column chromatography (10–15% EtOAc/Hexanes) obtained **3r** as a colorless oil; Yield: 0.157 g, 97% yield. ^1^H NMR (400 MHz, chloroform-*d*): δ 7.52–7.48 (m, 2H), 7.34–7.30 (m, 2H), 4.92–4.81 (m, 2H), 1.30 (dd, *J* = 14.0 and 6.0 Hz, 12H); ^13^C{H} NMR (100 MHz, chloroform-*d*): δ 135.9 (d, J = 5.0 Hz), 135.5, 129.3, 127.6 (d, *J* = 8.0 Hz), 74.2 (d, *J* = 7.0 Hz), 23.7 (d, *J* = 5.0 Hz), 23.4 (d, *J* = 5.0 Hz); ^31^P NMR (162 MHz, chloroform-*d*): δ 85.94.

### *S*-Benzyl *O*,*O*-diisopropyl phosphorodithioate (3s)^58^

The title compound was prepared following the general procedure for Scheme 2, *O,O*-diisopropyl phosphonothioate (**1b**, 0.5 mmol, 0.091 g), phenyl methanethiol (**2p**, 0.6 mmol, 0.075 g), and Cs_2_CO_3_ (10 mol %, 0.016 g), after column chromatography (10–15% EtOAc/Hexanes) obtained **3s** as a colorless oil; Yield: 0.078 g, 51% yield. ^1^H NMR (400 MHz, chloroform-*d*): δ 7.36–7.32 (m, 2H), 7.31–7.25 (m, 2H), 7.24–7.22 (m, 1H), 4.86–4.74 (m, 2H), 4.10 (d, *J* = 14.4 Hz, 2H), 1.30 (dd, *J* = 12.8 and 6.4 Hz, 12H); ^13^C{H} NMR (100 MHz, chloroform-*d*): δ 137.1 (d, *J* = 7.0 Hz), 129.0, 128.7, 127.6, 73.5 (d, *J* = 7.0 Hz), 38.0 (d, *J* = 4.0 Hz), 23.7 (d, *J* = 5.0 Hz), 23.4 (d, *J* = 6.0 Hz); ^31^P NMR (162 MHz, chloroform-*d*): δ 91.27.

### *O,O*-Dibutyl *S*-(4-chlorophenyl) phosphorodithioate (3t)^25^

The title compound was prepared following the general procedure for Scheme 2; *O,O*-dibutyl phosphonothioate (**1c**, 0.5 mmol, 0.105 g), 4-chlorobenzene thiol (**2c**, 0.6 mmol, 0.077 g), and Cs_2_CO_3_ (10 mol %, 0.016 g), after column chromatography (10–15% EtOAc/Hexanes) obtained **3t** as a colorless oil; Yield: 0.132 g, 75% yield. ^1^H NMR (400 MHz, chloroform-*d*): δ 7.44 (dd, *J* = 8.8 and 2.0 Hz, 2H), 7.33 (dd, *J* = 8.8 and 1.2 Hz, 2H), 4.21–4.04 (m, 4H), 1.67–1.60 (m, 4H), 1.41–1.31 (m, 4H), 0.92 (t, *J* = 7.6 Hz, 6H); ^13^C{H} NMR (100 MHz, chloroform-*d*): δ 136.0(d, *J* = 5.0 Hz), 135.8, 129.5 (d, *J* = 2.0 Hz), 126.9, 68.1 (d, *J* = 7.0 Hz), 31.9 (d, *J* = 8.0 Hz), 18.8, 13.7; ^31^P NMR (162 MHz, chloroform-*d*): δ 88.43.

### *S*-(4-Bromophenyl) *O,O*-dibutyl phosphorodithioate (3u)

The title compound was prepared following the general procedure for Scheme 2; *O,O*-dibutyl phosphonothioate (**1c**, 0.5 mmol, 0.105 g), 4-bromobenzenethiol (**2d**, 0.6 mmol, 0.113 g), and Cs_2_CO_3_ (10 mol %, 0.016 g), after column chromatography (10–15% EtOAc/Hexanes) obtained **3u** as a colorless oil; Yield: 0.107 g, 54% yield. ^1^H NMR (400 MHz, chloroform-*d*): δ 7.49–7.47 (m, 2H), 7.37 (d, *J* = 8.8 and 2.0 Hz, 2H), 4.21–4.04 (m, 4H), 1.67–1.60 (m, 4H), 1.40–1.31 (m, 4H), 0.91 (t, *J* = 7.6 Hz, 6H); ^13^C{H} NMR (100 MHz, chloroform-*d*): δ 136.2 (d, *J* = 5.0 Hz), 132.4 (d, *J* = 2.0 Hz), 127.6 (d, *J* = 8.0 Hz), 123.9 (d, *J* = 3.0 Hz), 68.1 (d, *J* = 7.0 Hz), 31.9 (d, *J* = 8.0 Hz), 18.8, 13.6; ^31^P NMR (162 MHz, chloroform-*d*): δ 88.16. HRMS (EI), calcd for C_14_H_22_BrO_2_PS_2_ [M]^+^ 395.9982, found 395.9986.

### *O,O*-Dibutyl *S*-(*p*-tolyl) phosphorodithioate (3v)

The title compound was prepared following the general procedure for Scheme 2; *O,O*-dibutyl phosphonothioate (**1c**, 0.5 mmol, 0.105 g), 4-methylbenzenethiol (**2g**, 0.6 mmol, 0.075 g), and Cs_2_CO_3_ (10 mol %, 0.016 g), after column chromatography (10–15% EtOAc/Hexanes) obtained **3v** as a colorless oil; Yield: 0.095 g, 57% yield. ^1^H NMR (400 MHz, chloroform-*d*): δ 7.39 (d, *J* = 8.4 Hz, 2H), 7.15 (dd, *J* = 8.4 and 0.8 Hz, 2H), 4.20–4.04 (m, 4H), 2.35 (d, *J* = 2.0 Hz, 3H), 1.67–1.58 (m, 4H), 1.40–1.31 (m, 4H), 0.91 (t, *J* = 7.6 Hz, 6H); ^13^C{H} NMR (100 MHz, chloroform-*d*): δ 139.5 (d, *J* = 3.0 Hz), 134.8 (d, *J* = 5.0 Hz), 130.0 (d, *J* = 3.0 Hz), 124.7 (d, *J* = 8.0 Hz), 67.9 (d, *J* = 6.0 Hz), 31.9 (d, *J* = 8.0 Hz), 21.3, 18.8, 13.6; ^31^P NMR (162 MHz, chloroform-*d*): δ 89.64. HRMS (EI), calcd for C_15_H_25_O_2_PS_2_ [M]^+^ 332.1034, found 332.1039.

### *O,O*-Diisobutyl *S*-phenyl phosphorodithioate (3w)^25^

The title compound was prepared following the general procedure for Scheme 2; *O,O*-diisobutyl phosphonothioate (**1d**, 0.5 mmol, 0.105 g), benzenethiol (**2a**, 0.6 mmol, 0.066 g), and Cs_2_CO_3_ (10 mol %, 0.016 g), after column chromatography (10–15% EtOAc/Hexanes) obtained **3w** as a colorless oil; Yield: 0.126 g, 79% yield. ^1^H NMR (400 MHz, chloroform-*d*): δ 7.55–7.51 (m, 2H), 7.36–7.34 (m, 3H), 3.97–3.91 (m, 2H), 3.87–3.81 (m, 2H), 1.98–1.88 (m, 2H), 0.89 (dd, *J* = 6.8 and 2.8 Hz, 12H); ^13^C{H} NMR (100 MHz, chloroform-*d*): δ 134.8 (d, *J* = 5.0 Hz), 129.27 (d, *J* = 2.0 Hz), 129.24, 128.3 (d, *J* = 7.0 Hz), 74.0 (d, *J* = 8.0 Hz), 28. Nine (d, *J* = 8.0 Hz), 18.9; ^31^P NMR (162 MHz, chloroform-*d*): δ 88.87.

### *S*-(4-Chlorophenyl) *O,O*-diisobutyl phosphorodithioate (3x)

The title compound was prepared following the general procedure for Scheme 2; *O,O*-diisobutyl phosphonothioate (**1d**, 0.5 mmol, 0.105 g), 4-chlorobenzene thiol (**2c**, 0.6 mmol, 0.077 g), and Cs_2_CO_3_ (10 mol %, 0.016 g), after column chromatography (10–15% EtOAc/Hexanes) obtained **3x** as a colorless oil; Yield: 0.141 g, 80%. ^1^H NMR (400 MHz, chloroform-*d*): δ 7.45–7.42 (m, 2H), 7.32–7.24 (m, 2H), 3.95–3.89 (m, 2H), 3.84–3.79 (m, 2H), 1.95–1.87 (m, 2H), 0.89 (dd, *J* = 6.8 and 2.0 Hz, 12H); ^13^C{H} NMR (100 MHz, chloroform-*d*): δ 136.1 (d, *J* = 5.0 Hz), 135.7 (d, *J* = 3.0 Hz), 129.4 (d, *J* = 2.0 Hz), 127.1 (d, J = 7.0 Hz), 74.2 (d, *J* = 7.0 Hz), 28.9 (d, *J* = 8.0 Hz), 18.9; ^31^P NMR (162 MHz, chloroform-*d*): δ 88.37. HRMS (EI), calcd for C_14_H_22_ClO_2_PS_2_ [M]^+^ 352.0487, found 352.0481.

### *S*-(4-Bromophenyl) *O,O*-diisobutyl phosphorodithioate (3y)

The title compound was prepared following the general procedure for Scheme 2; *O,O*-diisobutyl phosphonothioate (**1d**, 0.5 mmol, 0.105 g), 4-bromobenzene thiol (**2d**, 0.6 mmol, 0.113 g), and Cs_2_CO_3_ (10 mol %, 0.016 g), after column chromatography (10–15% EtOAc/Hexanes) obtained **3y** as a colorless oil. Yield: 0.151 g, 76%. ^1^H NMR (400 MHz, chloroform-*d*): δ 7.49–7.46 (m, 2H), 7.40–7.37 (m, 2H), 3.96–3.90 (m, 2H), 3.86–3.80 (m, 2H), 1.99–1.89 (m, 2H), 0.91 (dd, *J* = 6.8 and 2.0 Hz, 12H); ^13^C{H} NMR (100 MHz, chloroform-*d*): δ 136.3 (d, *J* = 5.0 Hz), 132.4 (d, *J* = 3.0 Hz), 127.5 (d, *J* = 7.0 Hz), 123.9 (d, *J* = 5.0 Hz), 74.2 (d, *J* = 7.0 Hz), 28.9 (d, *J* = 9.0 Hz), 18.8; 31P NMR (162 MHz, chloroform-*d*): δ 88.12. HRMS (EI), calcd for C_14_H_22_BrO_2_PS_2_ [M]^+^ 395.9982, found 395.9974.

## Data Availability

The data underlying this study are available in the published article and its Supporting Information.

## Supporting Information Available

Experimental procedures, characterization data, and spectra (PDF)
