# Supplementary material for: Cesium Carbonate-Catalyzed Oxidative Cross-Dehydrogenative Thiolation of Phosphonothioates
Source: J Org Chem. 2025 Feb 6;90(6):2534–41. doi: 10.1021/acs.joc.4c02718 (PMC11833867; doi:10.1021/acs.joc.4c02718)
Supplement: Supplementary file 1 — jo4c02718_si_001.pdf [file jo4c02718_si_001.pdf]

Supporting Information

Cesium Carbonate-Catalyzed Oxidative *Cross*-Dehydrogenative Thiolation of Phosphonothioates

Hsiu-Te Hung,[a] Rekha Bai,[a] Sung-Hung Lee,[a] Indrajit Karmakar[a] and Chin-Fa Lee*[a,b,c]

[a]*Department of Chemistry, National Chung Hsing University, Taichung-402202, Taiwan 402, Republic of China.*
[b]*i-Center for Advanced Science and Technology (iCAST), National Chung Hsing University, Taichung-402202, Taiwan 402, Republic of China*
[c]*Innovation and Development Center of Sustainable Agriculture (IDCSA), National Chung Hsing University, Taichung-402202, Taiwan 402, Republic of China*

*Corresponding author: Prof. Chin-Fa Lee
E-mail: cfalee@dragon.nchu.edu.tw; ORCID: http://orcid.org/0000-0003-0735-5691

1. **General Information** **S2**
2. **General procedure for Table 1** **S2**
3. **General procedure for Table 2** **S3**
4. **General procedure for gram-scale synthesis** **S3**
5. **NMR Spectra** **S16**
6. **Calculation of E-factors for all the synthesized compounds (3a-3y)** **S93**
7. **References** **S93**

1. General Information

All the reagents and chemicals were purchased at the highest quality from commercially suppliers and used without further purification. Yields refer to chromatographically and spectroscopically (^{1}H NMR) homogeneous material, unless otherwise stated. Reactions were monitored by GC/MS and thin layer chromatography (TLC). TLC was performed using 0.25 mm E. Merck silica plates ($60F_{254}$), using short-wave UV light as the visualizing agent or $KMnO_4$ and heat as developing agents. ^{1}H NMR, ^{13}C NMR, ^{19}F NMR, ^{31}P NMR, and HRMS techniques were used for the analysis of synthesized compounds. Chemical shifts reported in parts per million (ppm) with referencing the TMS at 0.00 ppm for ^{1}H NMR and coupling constants (*J*) were given in Hz. ^{1}H NMR peak signals were reported as s (singlet), br (broad), d (doublet), dd (double doublet), td (triplet of doublet), ddd (doublet of double doublet), qd (quartet of doublet), quint (pentet), sept (septet), and m (multiplet). In the ^{13}C NMR, chemical shifts were reported in ppm with referencing the center line of a triplet of chloroform-d at 77.10 ppm. High-resolution mass spectra (HRMS) were recorded on a Jeol JMS-HX 110 quadrupole type spectrometer provided by the National Chung Hsing University. GC-MS analyses were recorded on Agilent Technologies 5977A GC equipped with Agilent 7890B MS. All Melting points (M.P.) were observed by using a Büchi 535 apparatus. Column chromatography was performed over silica-gel (particle size: 100-200 Mesh) using hexanes and ethyl acetate as eluent. Starting materials **1a**-**1d**[1] were synthesized using reported literature.

2. General procedure for Table 1

To a reaction tube was added *O,O*-diethyl phosphonothioate (**1a**, 0.5 mmol, 0.077g), benzenethiol (2a, 0.6 mmol, 0.066 g), and base (7.0 mol% to 15 mol%) in solvent. The resulting solution was stirred at 30 ^{o}C in oil bath for 1-5 h under gas. After the completion of the reaction, the solvent was evaporated and the crude was diluted with water (20 mL) followed by extracted with ethyl acetate (3 × 20 mL). The combined organic layers were concentrated under reduced pressure to get crude product which were further purified through column chromatography using ethyl acetate/hexanes (1:9) as an eluent to afford the corresponding products **3a**.

O,O-Diethyl S-phenyl phosphorodithioate (3a)[1]

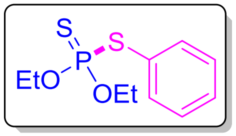

The title compound was prepared following the general procedure for Table 1, O,O-diethyl phosphonothioate (**1a**, 0.5 mmol, 0.077 g), benzenethiol (**2a**, 0.6 mmol, 0.066 g), and Cs_2CO_3 (10 mol%, 0.016 g), after column chromatography (10-15% EtOAc/Hexanes) obtained **3** as a colorless oil; Yield: 0.125 g, 95%. ^{1}H NMR (400 MHz, chloroform-d): δ 7.46-7.43 (m, 2H), 7.32-7.19 (m, 3H), 4.23-4.05 (m, 4H), 1.24 (td, J = 8.0 & 0.8 Hz, 6H); ^{13}C{H} NMR (100 MHz, chloroform-d): δ 134.7 (d, J = 5.0 Hz), 129.3 (d, J = 2.0 Hz), 128.3 (d, J = 8.0 Hz), 64.2 (d, J = 6.0 Hz), 15.7 (d, J = 8.0 Hz); ^{31}P NMR (162 MHz, chloroform-d) δ 88.66.

3. General procedure for Table 2

To a reaction tube was added phosphonate derivatives (0.5 mmol), thiols (0.6 mmol) and Cs_2CO_3 (10 mol%) in CH_3CN solvent. The resulting solution was stirred at 30 °C in oil bath for 3 h under oxygen gas ballon. After the completion of the reaction, the acetonitrile solvent was evaporated and the crude was diluted with water (20 mL) followed by extracted with ethyl acetate (3 × 20 mL). The combined organic layers were concentrated under reduced pressure to get crude products which were further purified through column chromatography using ethyl acetate/hexanes as an eluent to afford the corresponding products **3b-3y**

4. General procedure for gram-scale synthesis

To a reaction tube, O,O-diethyl phosphonothioate (**1a**, 5.0 mmol, 0.770 g), benzenethiol (**2a**, 6.0 mmol, 0.660 g), and Cs_2CO_3 (10 mol%) were added in 5 mL of acetonitrile (CH_3CN) solvent. The resulting solution was stirred at 30 °C in an oil bath for 5 hours under air. Upon completion of the reaction, the acetonitrile solvent was evaporated, and the residue was diluted with water (50 mL), followed by extraction with ethyl acetate (3 × 50 mL). The combined organic layers were concentrated under reduced pressure to yield the crude product, which was then purified by column chromatography using ethyl acetate/hexanes as the eluent. The corresponding product, O,O-diethyl S-phenyl phosphorodithioate (**3a**), was obtained in 80% yield (1.05 g).

_O,O_-Diethyl _S_-(4-fluorophenyl) phosphorodithioate (3b)[1]

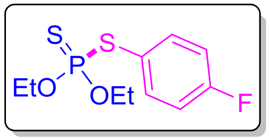

The title compound was prepared following the general procedure for Table 2, _O,O_-diethyl phosphonothioate (**1a**, 0.5 mmol, 0.077 g), 4-fluorobenzenethiol (**2b**, 0.6 mmol, 0.077 g), Cs_2CO_3 (10 mol%, 0.016 g), after column chromatography (10-15% EtOAc/Hexanes) obtained **3b** as a colorless oil; Yield: 0.102 g, 73% yield. ^{1}H NMR (400 MHz, chloroform-_d_): δ 7.52-7.48 (m, 2H), 7.06 (td, J = 8.4 & 0.8 Hz, 2H), 4.29-4.11 (m, 4H), 1.32 (td, J = 6.8 & 0.8 Hz, 6H); ^{13}C{H} NMR (100 MHz, chloroform-_d_): δ 163.55 (dd, J = 253 and 4 Hz), 136.7 (q, J = 5.0 Hz), 123.5 (q, J = 5.0 Hz), 116.6 (d, J = 3.0 Hz), 116.4 (d, J = 3.0 Hz), 64.4 (d, J = 6.0 Hz), 15.8 (d, J = 9.0 Hz); ^{31}P NMR (162 MHz, chloroform-_d_) δ 88.81 (d, J = 6.2 Hz); ^{19}F NMR (376 MHz, $CDCl_3$) δ -110.93.

_S_-(4-Chlorophenyl) _O,O_-diethyl phosphorodithioate (3c)[2]

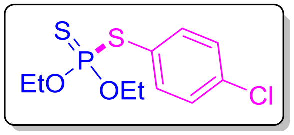

The title compound was prepared following the general procedure for Table 2, _O,O_-diethyl phosphonothioate (**1a**, 0.5 mmol, 0.077 g), 4-chlorobenzenethiol (**2c**, 0.6 mmol, 0.087 g), Cs_2CO_3 (10 mol%, 0.016 g), after column chromatography (10-15% EtOAc/Hexanes) obtained **3c** as a colorless oil; Yield: 0.129 g, 87% yield. ^{1}H NMR (400 MHz, chloroform-_d_): δ 7.45 (dd, J = 8.4 & 2.0 Hz, 2H), 7.34 (d, J = 8.4 Hz, 2H), 4.28-4.13 (m, 4H), 1.32 (dd, J = 8.4 & 1.2 Hz, 6H); ^{13}C{H} NMR (100 MHz, chloroform-_d_): δ 136.0 (d, J = 5.0 Hz), 135.8 (d, J = 4.0 Hz), 129.5 (d, J = 3.0 Hz), 126.8 (d, J = 8.0 Hz), 64.4 (d, J = 6.0 Hz), 15.8 (d, J = 8.0 Hz); ^{31}P NMR (162 MHz, chloroform-_d_) δ 88.06. HRMS (EI), calcd for $C_{10}H_{14}ClO_2PS_2$ $[M]^+$ 295.9861, found 295.9863.

S-(4-Bromophenyl) O,O-diethyl phosphorodithioate (3d)

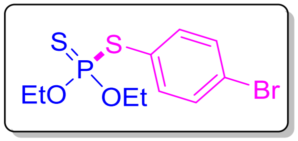

The title compound was prepared following the general procedure for Table 2, *O,O*-diethyl phosphonothioate (**1a**, 0.5 mmol, 0.077 g), 4-bromobenzenethiol (**2d**, 0.6 mmol, 0.113 g), Cs_2CO_3 (10 mol%, 0.016 g), after column chromatography (10-15% EtOAc/Hexanes) obtained **3d** as a color less oil; Yield: 0.104 g, 61% yield. ^{1}H NMR (400 MHz, chloroform-*d*) δ 7.51-7.47 (m, 2H), 7.39-7.26 (m, 2H), 4.29-4.11 (m, 4H), 1.32 (td, J = 7.2 & 0.8 Hz, 6H); ^{13}C{H} NMR (100 MHz, chloroform-*d*): δ 136.2 (d, J = 5.0 Hz), 132.5 (d, J = 3.0 Hz), 127.5 (d, J = 7.0 Hz), 124.0 (d, J = 4.0 Hz), 64.4 (d, J = 6.0 Hz), 15.8 (d, J = 8.0 Hz); ^{31}P NMR (162 MHz, chloroform-*d*): δ 87.79. HRMS (EI), calcd for $C_{10}H_{14}BrO_2PS_2$ $[M]^+$ 339.9356, found 339.9354.

S-(2-Chlorophenyl) O,O-diethyl phosphorodithioate (3e)

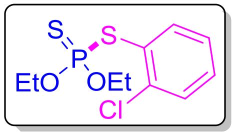

The title compound was prepared following the general procedure for Table 2, *O,O*-diethyl phosphonothioate (**1a**, 0.5 mmol, 0.077 g), 2-chlorobenzenethiol (**2e**, 0.6 mmol, 0.087 g), Cs_2CO_3 (10 mol%, 0.016 g), after column chromatography (10-15% EtOAc/Hexanes) obtained **3e** as a color less oil; Yield: 0.078 g, 53% yield. ^{1}H NMR (400 MHz, chloroform-*d*): δ 7.69 (dt, J = 8.0 & 2.4 Hz, 1H), 7.47 (dt, J = 7.6 & 0.8 Hz, 1H), 7.34-7.29 (m, 1H), 7.28-7.24 (m, 1H), 4.33-4.17 (m, 4H), 1.32 (td, J = 7.2 & 0.8 Hz, 6H); ^{13}C{H} NMR (100 MHz, chloroform-*d*): δ 138.1 (d, J = 6.0 Hz), 137.3 (d, J = 4.0 Hz), 130.7 (d, J = 3.0 Hz), 130.3 (d, J = 3.0 Hz), 128.0 (d, J = 9.0 Hz), 127.4 (d, J = 3.0 Hz), 64.5 (d, J = 5.0 Hz), 15.8 (d, J = 11.0 Hz); ^{31}P NMR (162 MHz, chloroform-*d*): δ 87.36. HRMS (EI), calcd for $C_{10}H_{14}ClO_2PS_2$ $[M]^+$ 295.9861, found, 295.9867.

***S*-(3-Chlorophenyl) *O,O*-diethyl phosphorodithioate (3f)**[3]

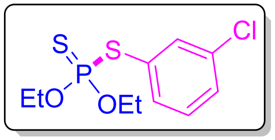

The title compound was prepared following the general procedure for Table 2, *O,O*-diethyl phosphonothioate (**1a**, 0.5 mmol, 0.077 g), 3-chlorobenzenethiol (**2f**, 0.6 mmol, 0.087 g), Cs_2CO_3 (10 mol%, 0.016 g), after column chromatography (10-15% EtOAc/Hexanes) obtained **3f** as a colorless oil; Yield: 0.106 g, 72% yield. ^{1}H NMR (400 MHz, chloroform-*d*): δ 7.51 (q, J = 2.0 Hz, 1H), 7.42-7.39 (m, 1H), 7.38-7.34 (m, 1H), 7.31-7.26 (m ,1H), 4.30-4.13 (m, 4H), 1.33 (t, J = 7.2 & 0.8 Hz, 6H); ^{13}C{H} NMR (100 MHz, chloroform-*d*): δ 134.7 (d, J = 2.0 Hz), 134.5 (d, J = 4.0 Hz), 132.8 (d, J = 4.0 Hz), 130.2 (d, J = 10.0 Hz), 130.1 (d, J = 7.0 Hz), 129.5 (d, J = 3.0 Hz), 64.5 (d, J = 6.0 Hz), 15.8 (d, J = 8.0 Hz); ^{31}P NMR (162 MHz, chloroform-*d*): δ 87.41. HRMS (EI), calcd for $C_{10}H_{14}ClO_2PS_2$ [M]+ 295.9861, found 295.9867.

***O,O*-Diethyl S-(*p*-tolyl) phosphorodithioate (3g)**[1]

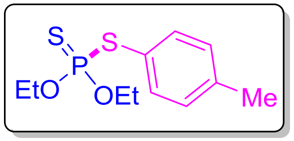

The title compound was prepared following the general procedure for Table 2, *O,O*-diethyl phosphonothioate (**1a**, 0.5 mmol, 0.077 g), 4-methylbenzenethiol (**2g**, 0.6 mmol, 0.075 g), Cs_2CO_3 (10 mol%, 0.016 g), after column chromatography (15-20% EtOAc/Hexanes) obtained **3g** as a color less oil; Yield: 0.120 g, 87% yield. ^{1}H NMR (400 MHz, chloroform-*d*): δ 7.39 (dt, J = 4.4 & 2.0 Hz, 2H), 7.18-7.15 (m, 2H), 4.27-4.14 (m, 4H), 2.35 (d, J = 2.4 Hz, 3H), 1.32 (td, J = 7.2 & 0.8 Hz, 6H); ^{13}C{H} NMR (100 MHz, chloroform-*d*): δ 139.7 (d, J = 2.0 Hz), 134.8 (d, J = 5.0 Hz), 130.1 (d, J = 2.0 Hz), 124.6 (d, J = 8.0 Hz), 64.1 (d, J = 5.0 Hz), 21.3, 15.8 (d, J = 9.0 Hz); ^{31}P NMR (162 MHz, chloroform-*d*): δ 89.28.

S-(2,4-Dimethylphenyl) O,O-diethyl phosphorodithioate (3h)

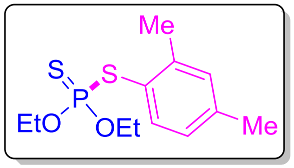

The title compound was prepared following the general procedure for Table 2, *O,O*-diethyl phosphonothioate (**1a**, 0.5 mmol, 0.077 g), 2,4-dimethylbenzenethiol (**2h**, 0.6 mmol, 0.083 g), Cs_2CO_3 (10 mol%, 0.016 g), after column chromatography (15-20% EtOAc/Hexanes) obtained **3h** as a color less oil; Yield: 0.096 g, 66% yield. ^{1}H NMR (400 MHz, chloroform-*d*): δ 7.40 (dd, J = 8.0 & 2.4 Hz, 1H), 7.08 (d, J = 2.0 Hz, 1H), 6.99 (dd, J = 8.0 & 2.0 Hz, 1H), 4.26-4.09 (m, 4H), 2.46 (d, J = 1.2 Hz, 3H), 2.31 (d, J = 2.8 Hz, 3H), 1.31 (td, J = 7.2 & 0.8 Hz, 6H); ^{13}C{H} NMR (100 MHz, chloroform-*d*): δ 142.0 (d, J = 5.0 Hz), 139.9 (d, J = 4.0 Hz), 136.4 (d, J = 4.0 Hz), 131.7 (d, J = 3.0 Hz), 127.5 (d, J = 3.0 Hz), 123.9 (d, J = 8.0 Hz), 64.2 (d, J = 7.0 Hz), 21.3, 21.2, 15.8 (d, J = 8.0 Hz); ^{31}P NMR (162 MHz, chloroform-*d*): δ 90.51. HRMS (EI), calcd for $C_{12}H_{19}O_2PS_2$ $[M]^+$ 290.0564, found 290.0560.

O,O-Diethyl S-(4-methoxyphenyl) phosphorodithioate (3i)[3]

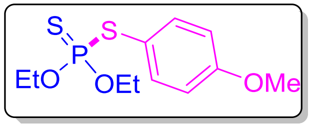

The title compound was prepared following the general procedure for Table 2, *O,O*-diethyl phosphonothioate (**1a**, 0.5 mmol, 0.077 g), 4-methoxybenzenethiol (**2i**, 0.6 mmol, 0.084 g), Cs_2CO_3 (10 mol%, 0.016 g), after column chromatography (25-30% EtOAc/Hexanes) obtained **3i** as a colorless oil; Yield: 0.084 g, 57% yield. ^{1}H NMR (400 MHz, chloroform-*d*): δ 7.43-7.40 (m, 2H), 6.89-6.87 (m, 2H), 4.27-4.13 (m, 4H), 3.81 (d, J = 1.2 Hz, 3H), 1.31 (td, J = 7.2 & 1.2 Hz, 6H); ^{13}C{H} NMR (100 MHz, chloroform-*d*): δ 160.7 (d, J = 1.0 Hz), 136.6 (d, J = 4.0 Hz), 118.6 (d, J = 9.0 Hz), 114.9 (d, J = 2.0 Hz), 64.1 (d, J = 5.0 Hz), 55.4, 15.8 (d, J = 9.0 Hz); ^{31}P NMR (162 MHz, chloroform-*d*): δ 89.67.

S-(4-Aminophenyl) O,O-diethyl phosphorodithioate (3j)[1]

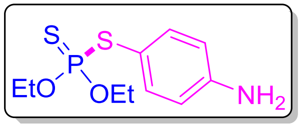

The title compound was prepared following the general procedure for Table 2, *O,O*-diethyl phosphonothioate (**1a**, 0.5 mmol, 0.077 g), 4-aminobenzenethiol (**2j**, 0.6 mmol, 0.075 g), Cs_2CO_3 (10 mol%, 0.016 g), after column chromatography (25-30% EtOAc/Hexanes) obtained **3j** as a colorless oil; Yield: 0.109 g, 79% yield. ^{1}H NMR (400 MHz, chloroform-*d*): δ 7.28-7.24 (m, 2H), 6.63 (d, J = 8.4 Hz, 2H), 4.27-4.09 (m, 4H), 3.82 (s, 2H), 1.31 (td, J = 7.2 & 0.8 Hz, 6H); ^{13}C{H} NMR (100 MHz, chloroform-*d*): δ 147.8, 136.5 (d, J = 4.0 Hz), 134.0, 115.5 (d, J = 3.0 Hz), 64.0 (d, J = 6.0 Hz), 15.8 (d, J = 9.0 Hz); ^{31}P NMR (162 MHz, chloroform-*d*): δ 90.41. HRMS (EI), calcd for $C_{10}H_{16}NO_2PS_2$ $[M]^+$ 277.0360, found 277.0356.

O,O-Diethyl S-(naphthalen-2-yl) phosphorodithioate (3k)

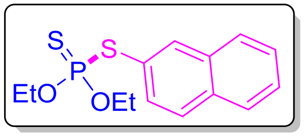

The title compound was prepared following the general procedure for Table 2, *O,O*-diethyl phosphonothioate (**1a**, 0.5 mmol, 0.077 g), naphthalene-2-thiol (**2l**, 0.6 mmol, 0.096 g), Cs_2CO_3 (10 mol%, 0.016 g), after column chromatography (20-25% EtOAc/Hexanes) obtained **3l** as a colorless oil; Yield: 0.099 g, 63% yield. ^{1}H NMR (400 MHz, chloroform-*d*): δ 8.03 (t, J = 2.8 Hz, 1H), 7.87-7.81 (m, 3H), 7.57-7.51 (m, 3H), 4.32-4.16 (m, 4H), 1.32 (td, J = 7.2 & 0.8 Hz, 6H); ^{13}C{H} NMR (100 MHz, chloroform-*d*): δ 134.8 (d, J = 6.0 Hz), 133.6 (d, J = 6.0 Hz), 133.2 (d, J = 3.0 Hz), 131.1 (d, J = 4.0 Hz), 128.9 (d, J = 3.0 Hz), 127.9 (d, J = 6.0 Hz), 127.2, 126.8, 125.5 (d, J = 8.0 Hz), 64.3 (d, J = 6.0 Hz), 15.8 (d, J = 8.0 Hz); ^{31}P NMR (162 MHz, chloroform-*d*): δ 88.64. HRMS (EI), calcd for $C_{14}H_{17}O_2PS_2$ $[M]^+$ 312.0408, found 312.0399.

***S*-Butyl *O,O*-diethyl phosphorodithioate (3l)**[1]

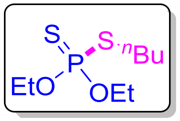

The title compound was prepared following the general procedure for Table 2, *O,O*-diethyl phosphonothioate (**1a**, 0.5 mmol, 0.077 g), butane-1-thiol (**2m**, 0.6 mmol, 0.054 g), Cs_2CO_3 (10 mol%, 0.016 g), after column chromatography (20-25% EtOAc/Hexanes) obtained **3m** as a colorless oil; Yield: 0.110 g, 91% yield. ^{1}H NMR (400 MHz, chloroform-*d*): δ 4.26-4.08 (m, 4H), 2.90-2.83 (m, 2H), 1.68-1.61 (m, 2H), 1.41-1.34 (m, 8H), 0.93 (t, J = 7.2 Hz, 3H); ^{13}C{H} NMR (100 MHz, chloroform-*d*): δ 63.8 (d, J = 6.0 Hz), 33.3 (d, J = 4.0 Hz), 32.4 (d, J = 6.0 Hz), 21.8, 15.9 (d, J = 8.0 Hz), 13.6; ^{31}P NMR (162 MHz, chloroform-*d*): δ 95.88. HRMS (EI), calcd for $C_8H_{19}O_2PS_2$ $[M]^+$ 242.0564, found .

***S*-Dodecyl *O,O*-diethyl phosphorodithioate (3m)**

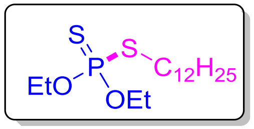

The title compound was prepared following the general procedure for Table 2, *O,O*-diethyl phosphonothioate (**1a**, 0.5 mmol, 0.077 g), dodecane-1-thiol (**2n**, 0.6 mmol, 0.121 g), Cs_2CO_3 (10 mol%, 0.016 g), after column chromatography (20-25% EtOAc/Hexanes) obtained **3n** as a color less oil; Yield: 0.142 g, 80% yield. ^{1}H NMR (400 MHz, chloroform-*d*): δ 4.26-4.08 (m, 4H), 2.89-2.81 (m, 2H), 1.70-1.62 (m, 2H), 1.39-1.34 (m, 6H), 1.25 (s, 18H), 0.88 (t, J = 7.2 Hz, 3H); ^{13}C{H} NMR (100 MHz, chloroform-*d*): δ 63.8 (d, J = 5.0 Hz), 33.6 (d, J = 4.0 Hz), 31.9, 30.4 (d, J = 5.0 Hz), 29.69, 29.62, 29.5, 29.4, 29.1, 28.7, 22.7, 15.9 (d, J = 9.0 Hz), 14.1; ^{31}P NMR (162 MHz, chloroform-*d*): δ 95.89. HRMS (EI), calcd for $C_{16}H_{35}O_2PS_2$ $[M]^+$ 354.1816, found 354.1823.

O,O-Diethyl S-hexyl phosphorodithioate (3n)

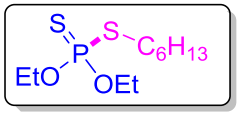

The title compound was prepared following the general procedure for Table 2, *O,O*-diethyl phosphonothioate (**1a**, 0.5 mmol, 0.077 g), hexane-1-thiol (**2o**, 0.6 mmol, 0.071 g), Cs_2CO_3 (10 mol%, 0.016 g), after column chromatography (20-25% EtOAc/Hexanes) obtained **3o** as a color less oil; Yield: 0.105 g, 78% yield. ^{1}H NMR (400 MHz, chloroform-*d*): δ 4.24-4.09 (m, 4H), 2.89-2.81 (m, 2H), 1.68-1.62 (m, 2H), 1.38-1.34 (m, 8H), 1.31-1.25 (m, 4H), 0.90-0.87 (m, 3H); ^{13}C{H} NMR (100 MHz, chloroform-*d*): δ 63.9 (*d*, J = 6.0 Hz), 33.7 (d, J = 4.0 Hz), 31.3, 30.4 (d, J = 5.0 Hz), 28.4, 22.6, 15.9 (d, J = 8.0 Hz), 14.1; ^{31}P NMR (162 MHz, chloroform-*d*): δ 95.85. HRMS (EI), calcd for $C_{10}H_{23}O_2PS_2$ $[M+1]^+$ 271.0911, found 271.0945.

S-Benzyl O,O-diethyl phosphorodithioate (3o)[4]

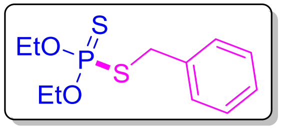

The title compound was prepared following the general procedure for Table 2, *O,O*-diethyl phosphonothioate (**1a**, 0.5 mmol, 0.077 g), phenyl methanethiol (**2p**, 0.6 mmol, 0.075 g), Cs_2CO_3 (10 mol%, 0.016 g), after column chromatography (10-15% EtOAc/Hexanes) obtained **3p** as a colorless oil; Yield: 0.076 g, 55% yield. ^{1}H NMR (400 MHz, chloroform-*d*): δ 7.36-7.29 (m, 3H), 7.28-7.25 (m, 2H), 4.18-3.96 (m, 6H), 1.28 (t, J = 7.2 Hz, 6H); ^{13}C{H} NMR (100 MHz, chloroform-*d*): δ 137.2, 129.0, 128.6, 127.6, 63.9 (d, J = 6.0 Hz), 37.6 (d, J = 4.0 Hz), 15.8 (d, J = 8.0 Hz); ^{31}P NMR (162 MHz, chloroform-*d*): δ 93.90 HRMS (EI), calcd for $C_{11}H_{17}O_2PS_2$ $[M]^+$ 276.0408, found 276.0399.

_O,O_-Diisopropyl _S_-phenyl phosphorodithioate (3p)[1]

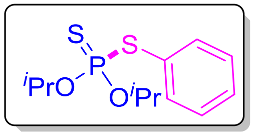

The title compound was prepared following the general procedure for Table 2, *O,O*-diisopropyl phosphonothioate (**1b**, 0.5 mmol, 0.091 g), benzenethiol (**2a**, 0.6 mmol, 0.066 g), and Cs_2CO_3 (10 mol%, 0.016 g), after column chromatography (10-15% EtOAc/Hexanes) obtained **3q** as a colorless oil; Yield: 0.137 g, 94% yield. ^{1}H NMR (400 MHz, chloroform-*d*): δ 7.59-7.55 (m, 2H), 7.36-7.26 (m, 3H), 4.91-4.81 (m, 2H), 1.32 (dd, J = 6.4 & 2.0 Hz, 6H), 1.27 (dd, J = 6.0 & 2.0 Hz, 6H); ^{13}C{H} NMR (100 MHz, chloroform-*d*): δ 134.6 (d, J = 5.0 Hz), 129.1 (d, J = 3.0 Hz), 129.0 (d, J = 3.0 Hz), 74.0 (d, J = 7.0 Hz), 23.8 (d, J = 4.0 Hz), 23.4 (d, J = 6.0 Hz); ^{31}P NMR (162 MHz, chloroform-*d*): δ 86.56.

_S_-(4-Fluorophenyl) _O,O_-diisopropyl phosphorodithioate (3q)

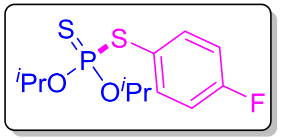

The title compound was prepared following the general procedure for Table 2, *O,O*-diisopropyl phosphonothioate (**1b**, 0.5 mmol, 0.091 g), 4-fluorobenzenethiol (**2b**, 0.6 mmol, 0.077 g), and Cs_2CO_3 (10 mol%, 0.016 g), after column chromatography (10-15% EtOAc/Hexanes) obtained **3r** as a colorless oil; Yield: 0.136 g, 88% yield. ^{1}H NMR (400 MHz, chloroform-*d*): δ 7.57-7.53 (m, 2H), 7.08-7.03 (m, 2H), 4.91-4.79 (m, 2H), 1.32 (d, J = 6.0 Hz, 6H), 1.26 (d, J = 6.0 Hz, 6H); ^{13}C{H} NMR (100 MHz, chloroform-*d*): δ 163.6 (d, J = 254 Hz), 136.9 (dd, J = 8.0 & 5.0 Hz), 124.1 (dd, J = 8.0 & 4.0 Hz), 116.2 (dd, J = 22.0 & 3.0 Hz), 74.1 (d, J = 8.0 Hz), 23.7 (d, J = 4.0 Hz), 23.4 (d, J = 4.0 Hz); ^{31}P NMR (162 MHz, chloroform-*d*): δ 86.82(d, J = 6.1Hz); ^{19}F NMR (376 MHz, chloroform-*d*): δ -111.47.... HRMS (EI), calcd for $C_{12}H_{18}FO_2PS_2$ $[M]^+$ 308.0470, found 308.0465

***S*-(4-Chlorophenyl) *O,O*-diisopropyl phosphorodithioate (3r)**[3]

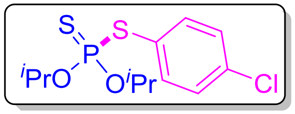

The title compound was prepared following the general procedure for Table 2, *O,O*-diisopropyl phosphonothioate (**1b**, 0.5 mmol, 0.091 g), 4-chlorobenzenethiol (**2c**, 0.6 mmol, 0.087 g), and Cs_2CO_3 (10 mol%, 0.016 g), after column chromatography (10-15% EtOAc/Hexanes) obtained **3s** as a color less oil; Yield: 0.157 g, 97% yield. ^{1}H NMR (400 MHz, chloroform-*d*): δ 7.52-7.48 (m, 2H), 7.34-7.30 (m, 2H), 4.92-4.81 (m, 2H), 1.30 (dd, J = 14.0 & 6.0 Hz, 12H); ^{13}C{H} NMR (100 MHz, chloroform-*d*): δ 135.9 (d, J = 5.0 Hz), 135.5, 129.3, 127.6 (d, J = 8.0 Hz), 74.2 (d, J = 7.0 Hz), 23.7 (d, J = 5.0 Hz), 23.4 (d, J = 5.0 Hz); ^{31}P NMR (162 MHz, chloroform-*d*): δ 85.94.

***S*-Benzyl *O,O*-diisopropyl phosphorodithioate (3s)**[5]

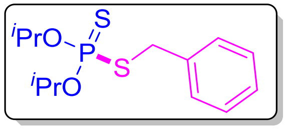

The title compound was prepared following the general procedure for Table 2, *O,O*-diisopropyl phosphonothioate (**1b**, 0.5 mmol, 0.091 g), phenyl methanethiol (**2p**, 0.6 mmol, 0.075 g), and Cs_2CO_3 (10 mol%, 0.016 g), after column chromatography (10-15% EtOAc/Hexanes) obtained **3t** as a colorless oil; Yield: 0.078 g, 51% yield. ^{1}H NMR (400 MHz, chloroform-*d*): δ 7.36-7.32 (m, 2H), 7.31-7.25 (m, 2H), 7.24-7.22 (m, 1H), 4.86-4.74 (m, 2H), 4.10 (d, J = 14.4 Hz, 2H), 1.30 (dd, J = 12.8 & 6.4 Hz, 12H); ^{13}C{H} NMR (100 MHz, chloroform-*d*): δ 137.1 (d, J = 7.0 Hz), 129.0, 128.7, 127.6, 73.5 (d, J = 7.0 Hz), 38.0 (d, J = 4.0 Hz), 23.7 (d, J = 5.0 Hz), 23.4 (d, J = 6.0 Hz); ^{31}P NMR (162 MHz, chloroform-*d*): δ 91.27.

_O,O_-Dibutyl _S_-(4-chlorophenyl) phosphorodithioate (3t)[1]

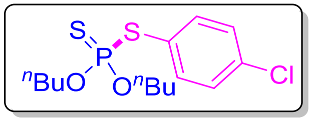

The title compound was prepared following the general procedure for Table 2, *O,O*-dibutyl phosphonothioate (**1c**, 0.5 mmol, 0.105 g), 4-chlorobenzene thiol (**2c**, 0.6 mmol, 0.077 g), and Cs_2CO_3 (10 mol%, 0.016 g), after column chromatography (10-15% EtOAc/Hexanes) obtained **3u** as a color less oil; Yield: 0.132 g, 75% yield. ^{1}H NMR (400 MHz, chloroform-*d*): δ 7.44 (dd, J = 8.8 & 2.0 Hz, 2H), 7.33 (dd, J = 8.8 &1.2 Hz, 2H), 4.21-4.04 (m, 4H), 1.67-1.60 (m, 4H), 1.41-1.31 (m, 4H), 0.92 (t, J = 7.6 Hz, 6H); ^{13}C{H} NMR (100 MHz, chloroform-*d*): δ 136.0(d, J = 5.0 Hz), 135.8, 129.5 (d, J = 2.0 Hz), 126.9, 68.1 (d, J = 7.0 Hz), 31.9 (d, J = 8.0 Hz), 18.8, 13.7; ^{31}P NMR (162 MHz, chloroform-*d*): δ 88.43.

_S_-(4-Bromophenyl) _O,O_-dibutyl phosphorodithioate (3u)

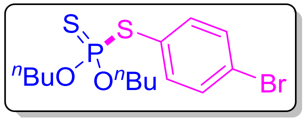

The title compound was prepared following the general procedure for Table 2, *O,O*-dibutyl phosphonothioate (**1c**, 0.5 mmol, 0.105 g), 4-bromobenzenethiol (**2d**, 0.6 mmol, 0.113 g), and Cs_2CO_3 (10 mol%, 0.016 g), after column chromatography (10-15% EtOAc/Hexanes) obtained **3v** as a color less oil; Yield: 0.107 g, 54% yield. ^{1}H NMR (400 MHz, chloroform-*d*): δ 7.49-7.47 (m, 2H), 7.37 (d, J = 8.8 & 2.0 Hz, 2H), 4.21-4.04 (m, 4H), 1.67-1.60 (m, 4H), 1.40-1.31 (m, 4H), 0.91 (t, J = 7.6 Hz, 6H); ^{13}C{H} NMR (100 MHz, chloroform-*d*): δ 136.2 (d, J = 5.0 Hz), 132.4 (d, J = 2.0 Hz), 127.6 (d, J = 8.0 Hz), 123.9 (d, J = 3.0 Hz), 68.1 (d, J = 7.0 Hz), 31.9 (d, J = 8.0 Hz), 18.8, 13.6; ^{31}P NMR (162 MHz, chloroform-*d*): δ 88.16. HRMS (EI), calcd for $C_{14}H_{22}BrO_2PS_2$ $[M]^+$ 395.9982, found 395.9986.

_O,O_-Dibutyl _S_-(_p_-tolyl) phosphorodithioate (3v)

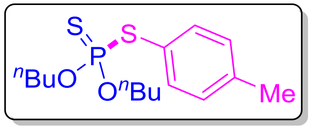

The title compound was prepared following the general procedure for Table 2, _O,O_-dibutyl phosphonothioate (**1c**, 0.5 mmol, 0.105 g), 4-methylbenzenethiol (**2g**, 0.6 mmol, 0.075 g), and Cs_2CO_3 (10 mol%, 0.016 g), after column chromatography (10-15% EtOAc/Hexanes) obtained **3w** as a color less oil; Yield: 0.095 g, 57% yield. ^{1}H NMR (400 MHz, chloroform-_d_): δ 7.39 (d, J = 8.4 Hz, 2H), 7.15 (dd, J = 8.4 & 0.8 Hz, 2H), 4.20-4.04 (m, 4H), 2.35 (d, J = 2.0 Hz, 3H), 1.67-1.58 (m, 4H), 1.40-1.31 (m, 4H), 0.91 (t, J = 7.6 Hz, 6H); ^{13}C{H} NMR (100 MHz, chloroform-_d_): δ 139.5 (d, J = 3.0 Hz), 134.8 (d, J = 5.0 Hz), 130.0 (d, J = 3.0 Hz), 124.7 (d, J = 8.0 Hz), 67.9 (d, J = 6.0 Hz), 31.9 (d, J = 8.0 Hz), 21.3, 18.8, 13.6; ^{31}P NMR (162 MHz, chloroform-_d_): δ 89.64. HRMS (EI), calcd for $C_{15}H_{25}O_2PS_2$ $[M]^+$ 332.1034, found 332.1039.

_O,O_-Diisobutyl _S_-phenyl phosphorodithioate (3w)[1]

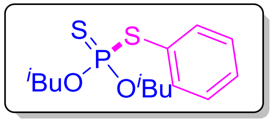

The title compound was prepared following the general procedure for Table 2, _O,O_-diisobutyl phosphonothioate (**1d**, 0.5 mmol, 0.105 g), benzenethiol (**2a**, 0.6 mmol, 0.066 g), and Cs_2CO_3 (10 mol%, 0.016 g), after column chromatography (10-15% EtOAc/Hexanes) obtained **3x** as a color less oil; Yield: 0.126 g, 79% yield. ^{1}H NMR (400 MHz, chloroform-_d_): δ 7.55-7.51 (m, 2H), 7.36– 7.34 (m, 3H), 3.97-3.91 (m, 2H), 3.87-3.81 (m, 2H), 1.98-1.88 (m, 2H), 0.89 (dd, J = 6.8 & 2.8 Hz, 12H); ^{13}C{H} NMR (100 MHz, chloroform-_d_): δ 134.8 (d, J = 5.0 Hz), 129.27 (d, J = 2.0 Hz), 129.24, 128.3 (d, J = 7.0 Hz), 74.0 (d, J = 8.0 Hz), 28. 9 (d, J = 8.0 Hz), 18.9; ^{31}P NMR (162 MHz, chloroform-_d_): δ 88.87.

_S_-(4-Chlorophenyl) _O,O_-diisobutyl phosphorodithioate (3x)

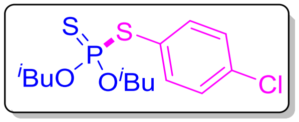

The title compound was prepared following the general procedure for Table 2, *O,O*-diisobutyl phosphonothioate (**1d**, 0.5 mmol, 0.105 g), 4-chlorobenzene thiol (**2c**, 0.6 mmol, 0.077 g), and Cs_2CO_3 (10 mol%, 0.016 g), after column chromatography (10-15% EtOAc/Hexanes) obtained **3y** as a colorless oil; Yield: 0.141 g, 80%. ^{1}H NMR (400 MHz, chloroform-*d*): δ 7.45-7.42 (m, 2H), 7.32-7.24 (m, 2H), 3.95-3.89 (m, 2H), 3.84-3.79 (m, 2H), 1.95-1.87 (m, 2H), 0.89 (dd, J = 6.8 & 2.0 Hz, 12H); ^{13}C{H} NMR (100 MHz, chloroform-*d*): δ 136.1 (d, J = 5.0 Hz), 135.7 (d, J = 3.0 Hz), 129.4 (d, J = 2.0 Hz), 127.1 (d, J = 7.0 Hz), 74.2 (d, J = 7.0 Hz), 28.9 (d, J = 8.0 Hz), 18.9; ^{31}P NMR (162 MHz, chloroform-*d*): δ 88.37. HRMS (EI), calcd for $C_{14}H_{22}ClO_2PS_2$ $[M]^+$ 352.0487, found 352.0481.

_S_-(4-Bromophenyl) _O,O_-diisobutyl phosphorodithioate (3y)

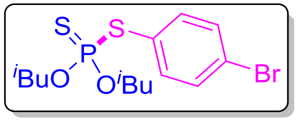

The title compound was prepared following the general procedure for Table 2, *O,O*-diisobutyl phosphonothioate (**1d**, 0.5 mmol, 0.105 g), 4-bromobenzene thiol (**2d**, 0.6 mmol, 0.113 g), and Cs_2CO_3 (10 mol%, 0.016 g), after column chromatography (10-15% EtOAc/Hexanes) obtained **27** as a colorless oil. Yield: 0.151 g, 76%. ^{1}H NMR (400 MHz, chloroform-*d*): δ 7.49-7.46 (m, 2H), 7.40-7.37 (m, 2H), 3.96-3.90 (m, 2H), 3.86-3.80 (m, 2H), 1.99-1.89 (m, 2H), 0.91 (dd, J = 6.8 & 2.0 Hz, 12H); ^{13}C{H} NMR (100 MHz, chloroform-*d*): δ 136.3 (d, J = 5.0 Hz), 132.4 (d, J = 3.0 Hz), 127.5 (d, J = 7.0 Hz), 123.9 (d, J = 5.0 Hz), 74.2 (d, J = 7.0 Hz), 28.9 (d, J = 9.0 Hz), 18.8; 31P NMR (162 MHz, chloroform-*d*): δ 88.12. HRMS (EI), calcd for $C_{14}H_{22}BrO_2PS_2$ $[M]^+$ 395.9982, found 395.9974.

5. ^{1}H NMR, ^{13}C NMR, ^{31}P NMR & ^{19}F NMR spectra of compounds 3a-3y

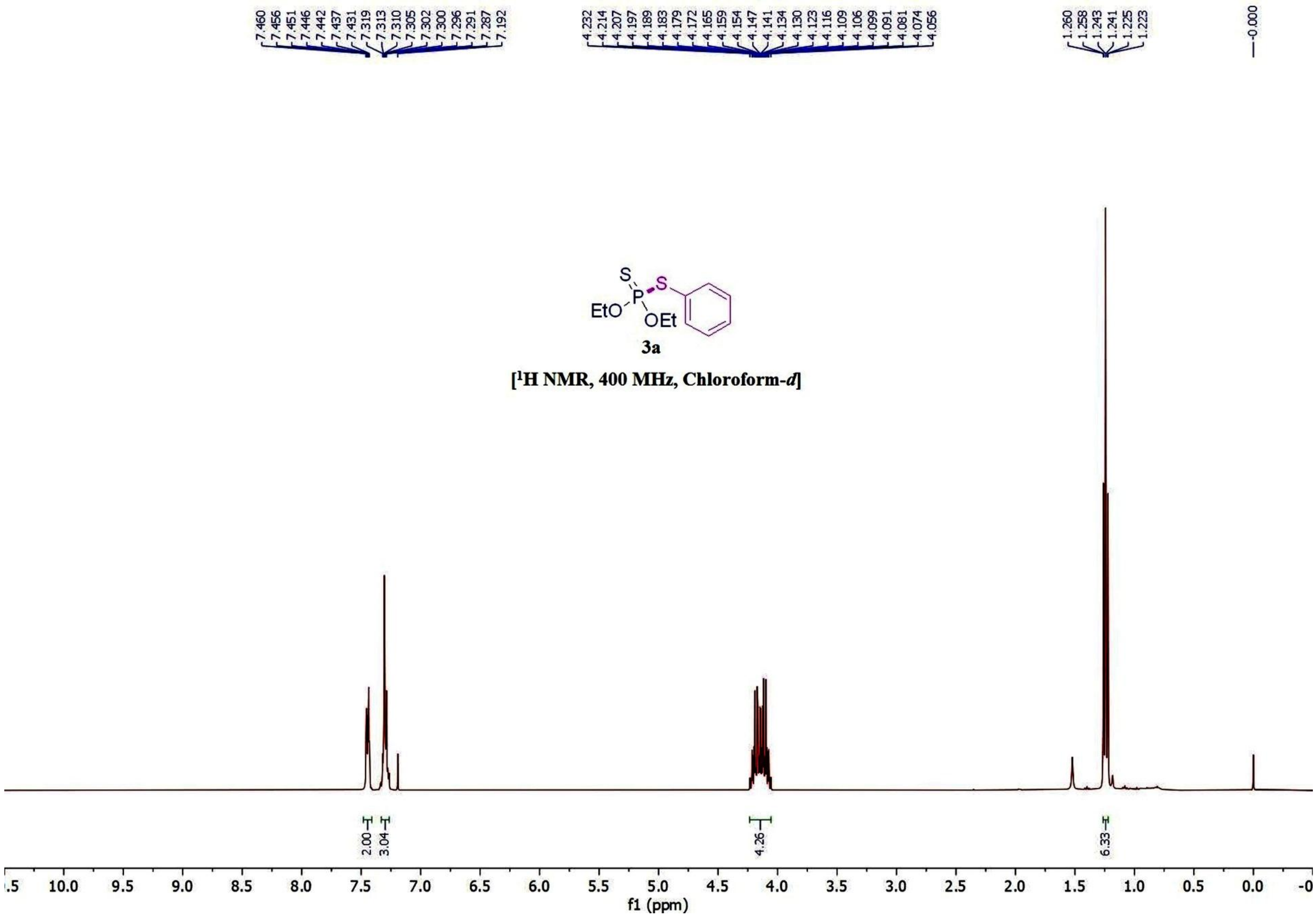

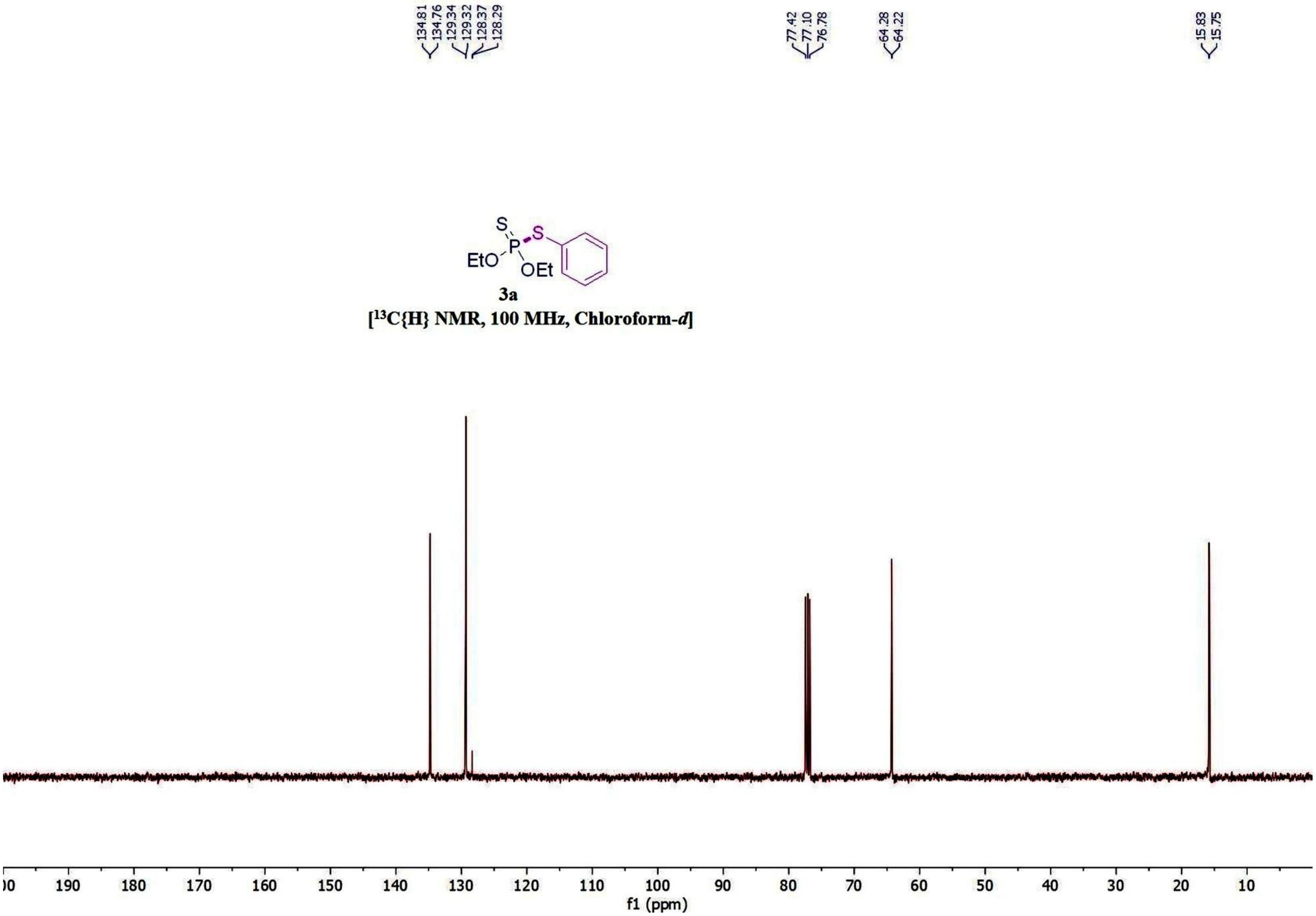

3a
[13C{H} NMR, 100 MHz, Chloroform-d]

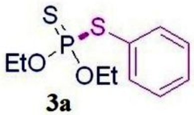

3a

[^{31}P NMR, 162 MHz, Chloroform-*d*]

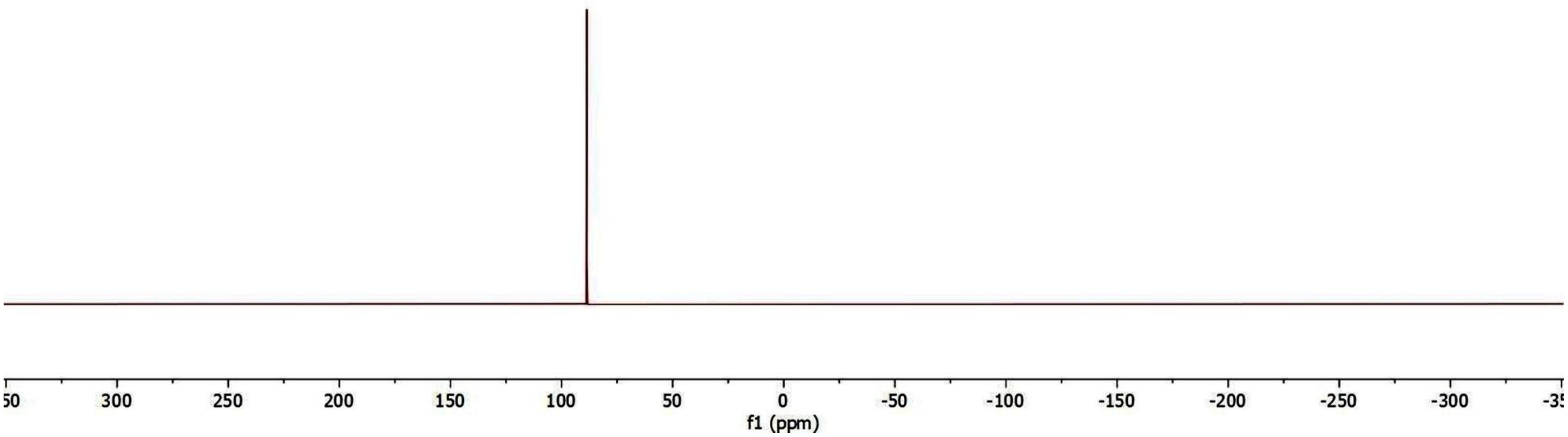

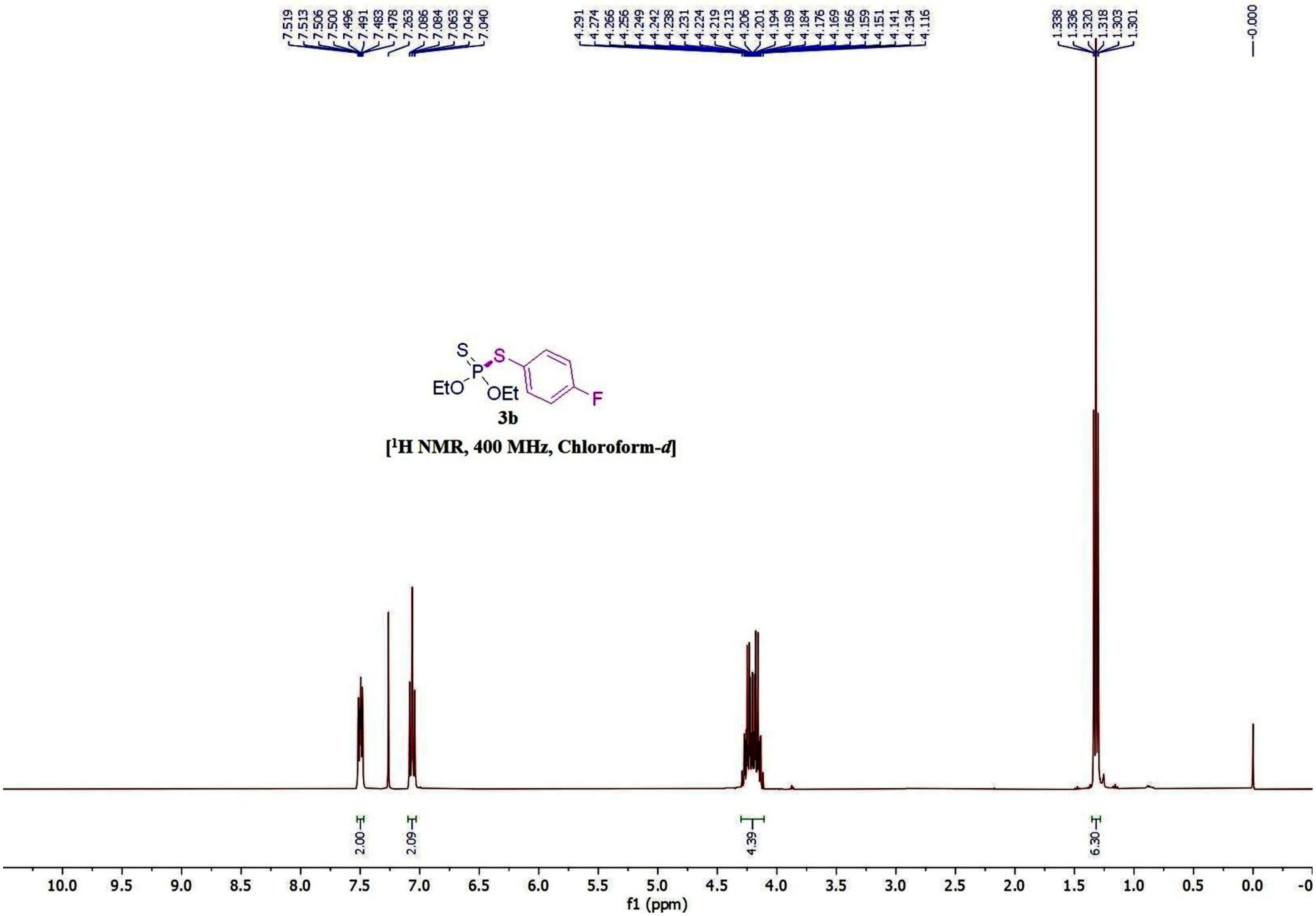

3b

[^{1}H NMR, 400 MHz, Chloroform-*d*]

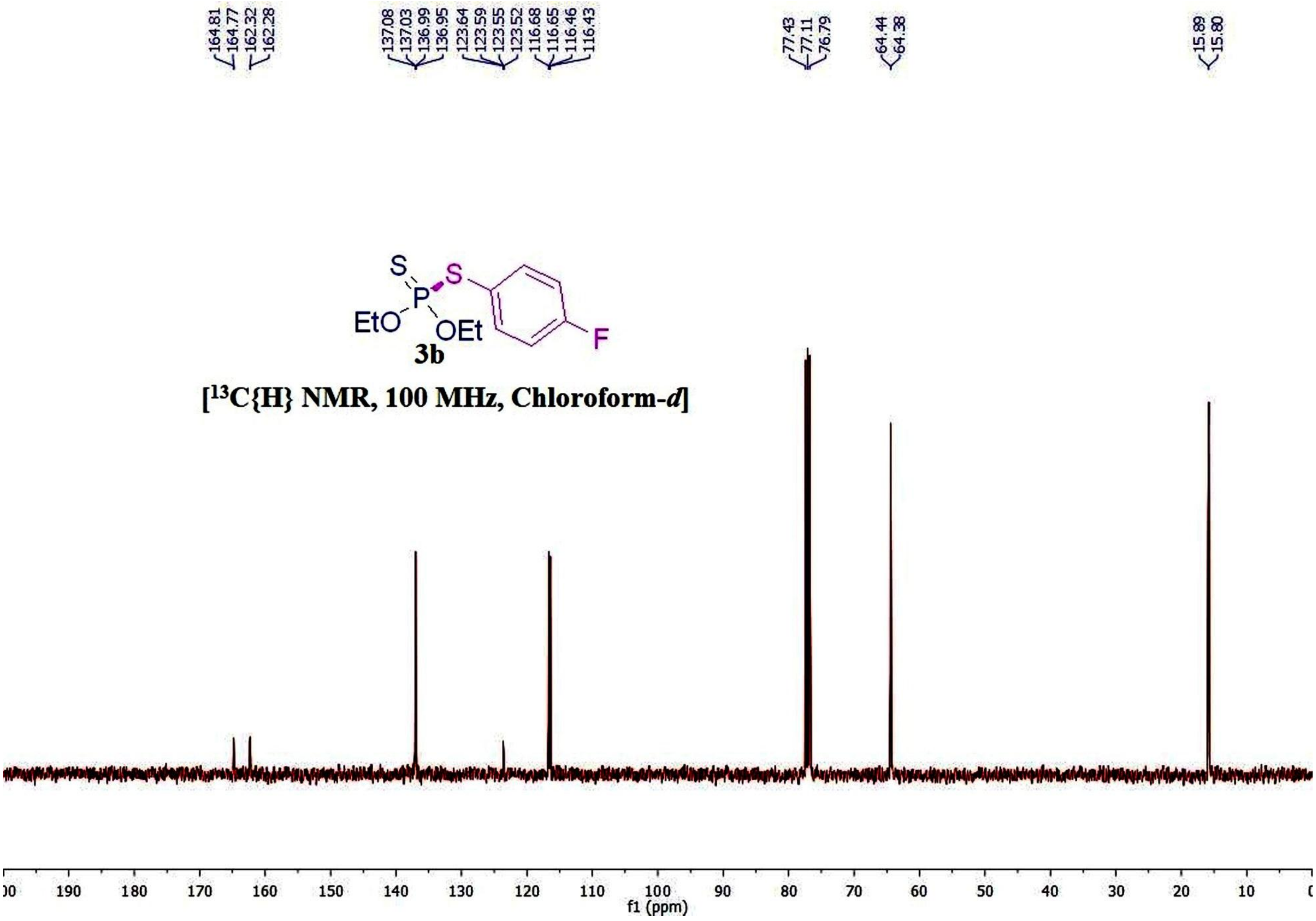

164.81
164.77
162.32
162.28
137.08
137.03
136.99
136.95
123.64
123.59
123.55
123.52
116.68
116.65
116.46
116.43
77.43
77.11
76.79
64.44
64.38
15.89
15.80
S
S
P
EtO
OEt
F
3b
[^{13}C{H} NMR, 100 MHz, Chloroform-*d*]
190
180
170
160
150
140
130
120
110
100
90
80
70
60
50
40
30
20
10
f1 (ppm)

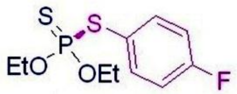

3b

[^{31}P NMR, 162 MHz, Chloroform-*d*]

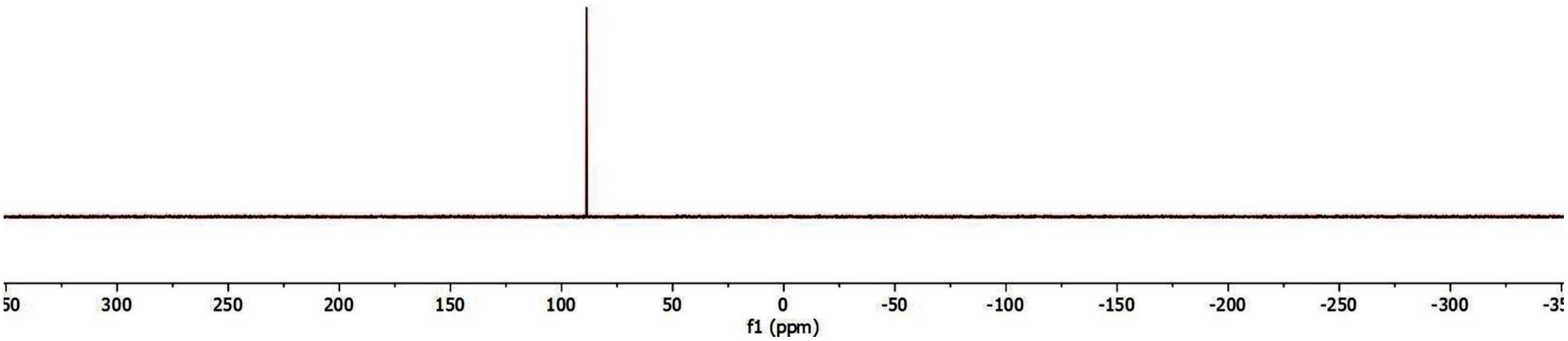

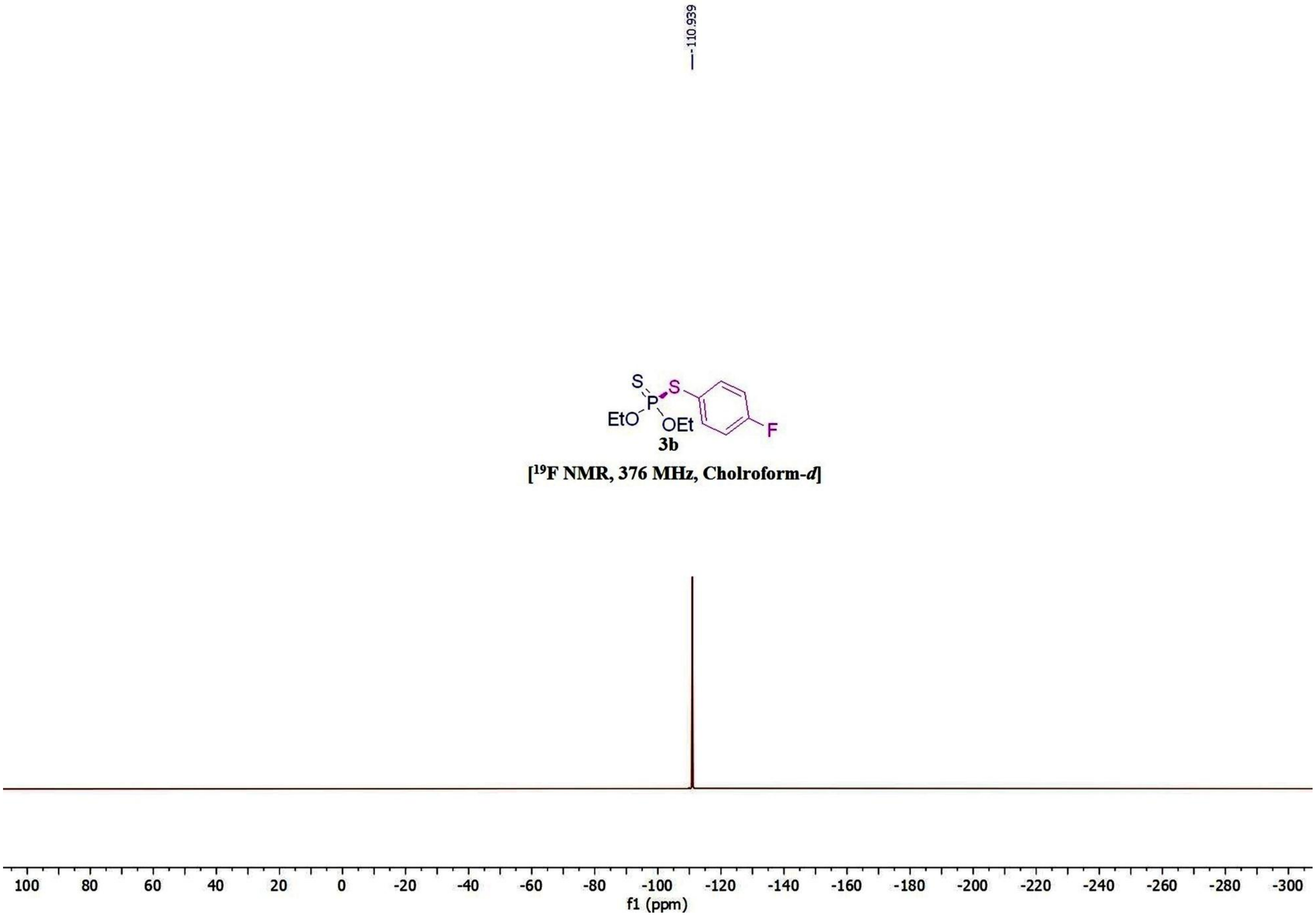

3b

[^{19}F NMR, 376 MHz, Cholroform-*d*]

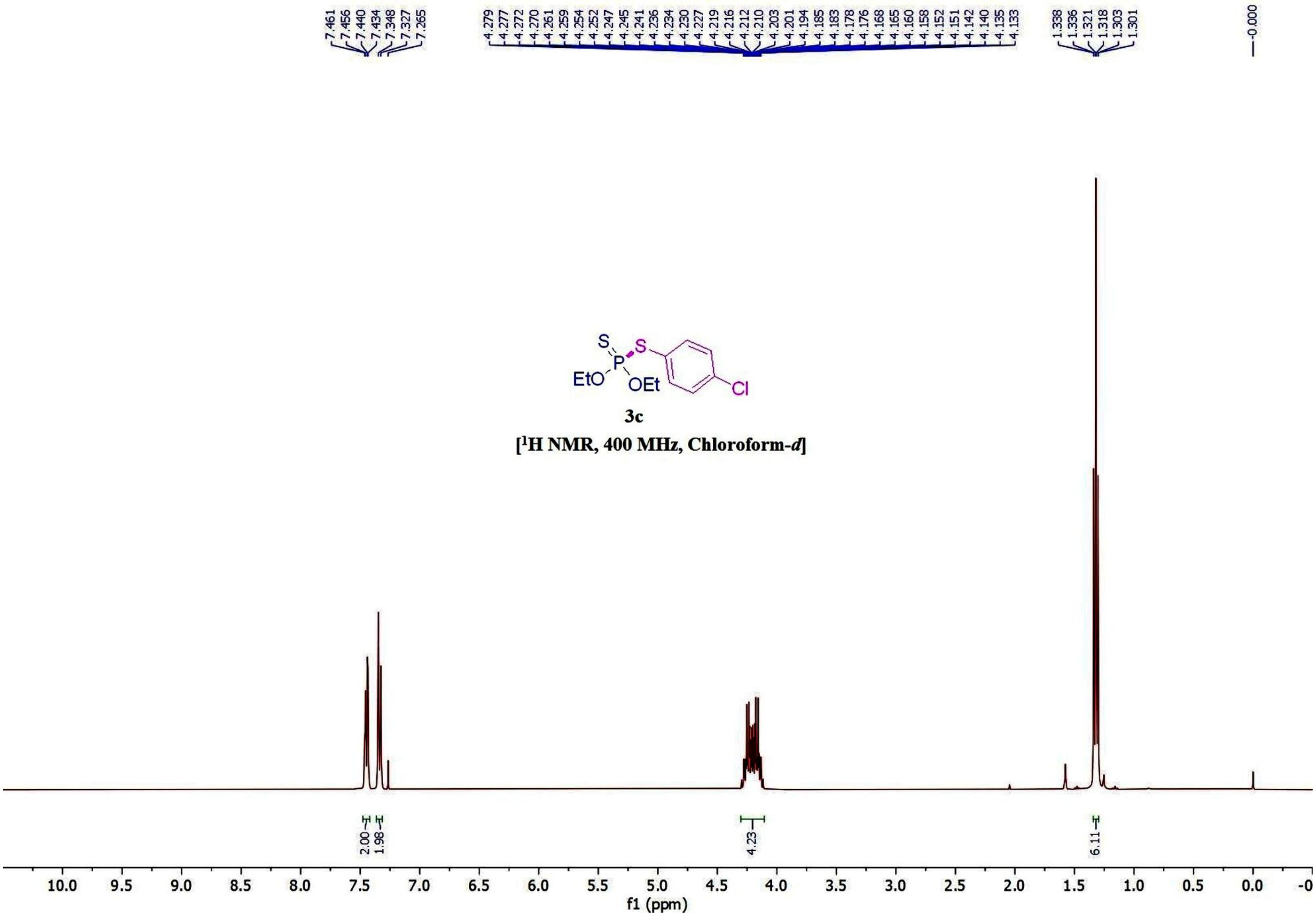

3c

[^{1}H NMR, 400 MHz, Chloroform-*d*]

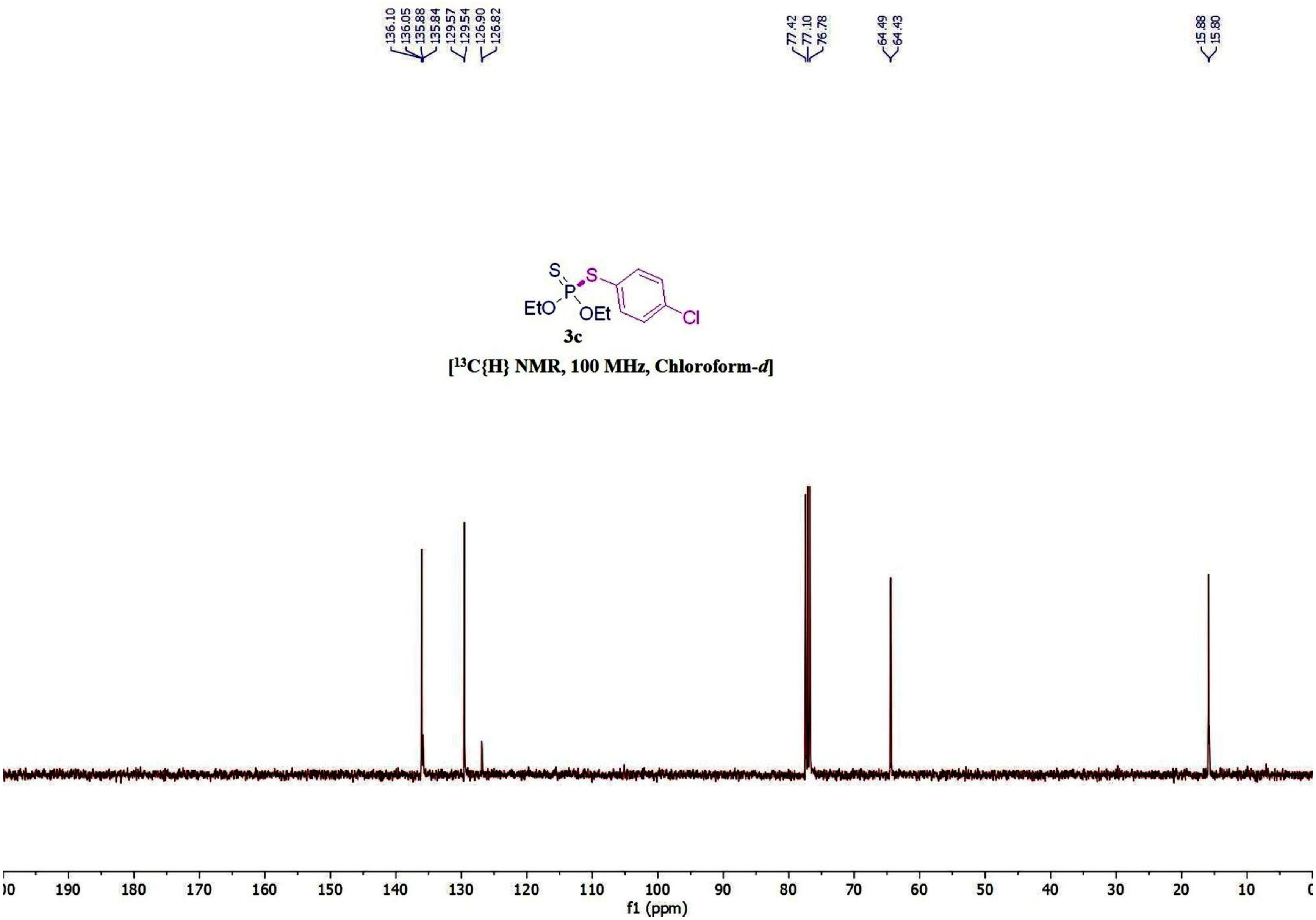

3c

[^{13}C{H} NMR, 100 MHz, Chloroform-*d*]

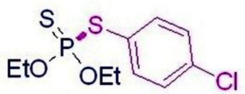

3c

[^{31}P NMR, 162 MHz, Chloroform-*d*]

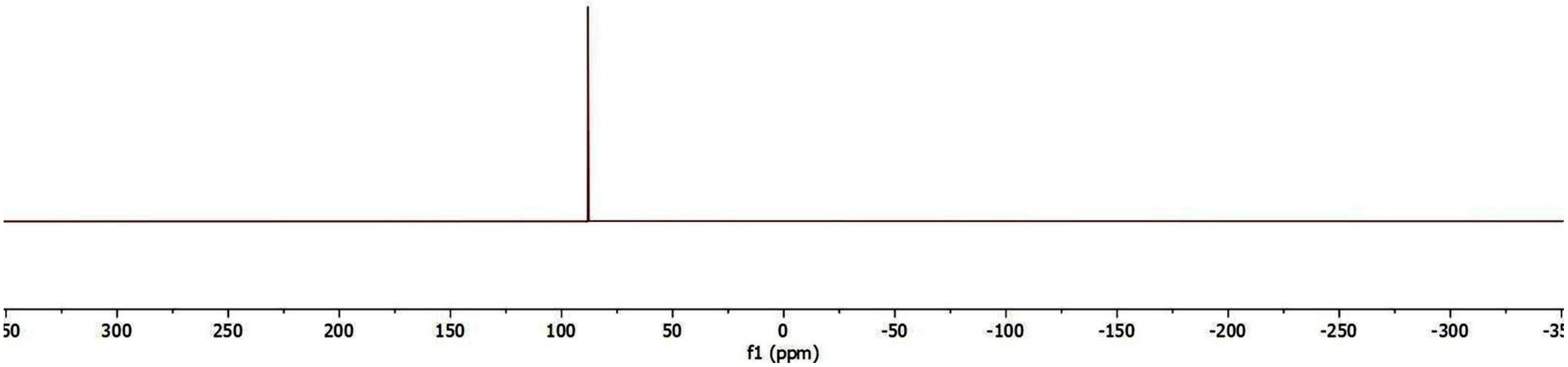

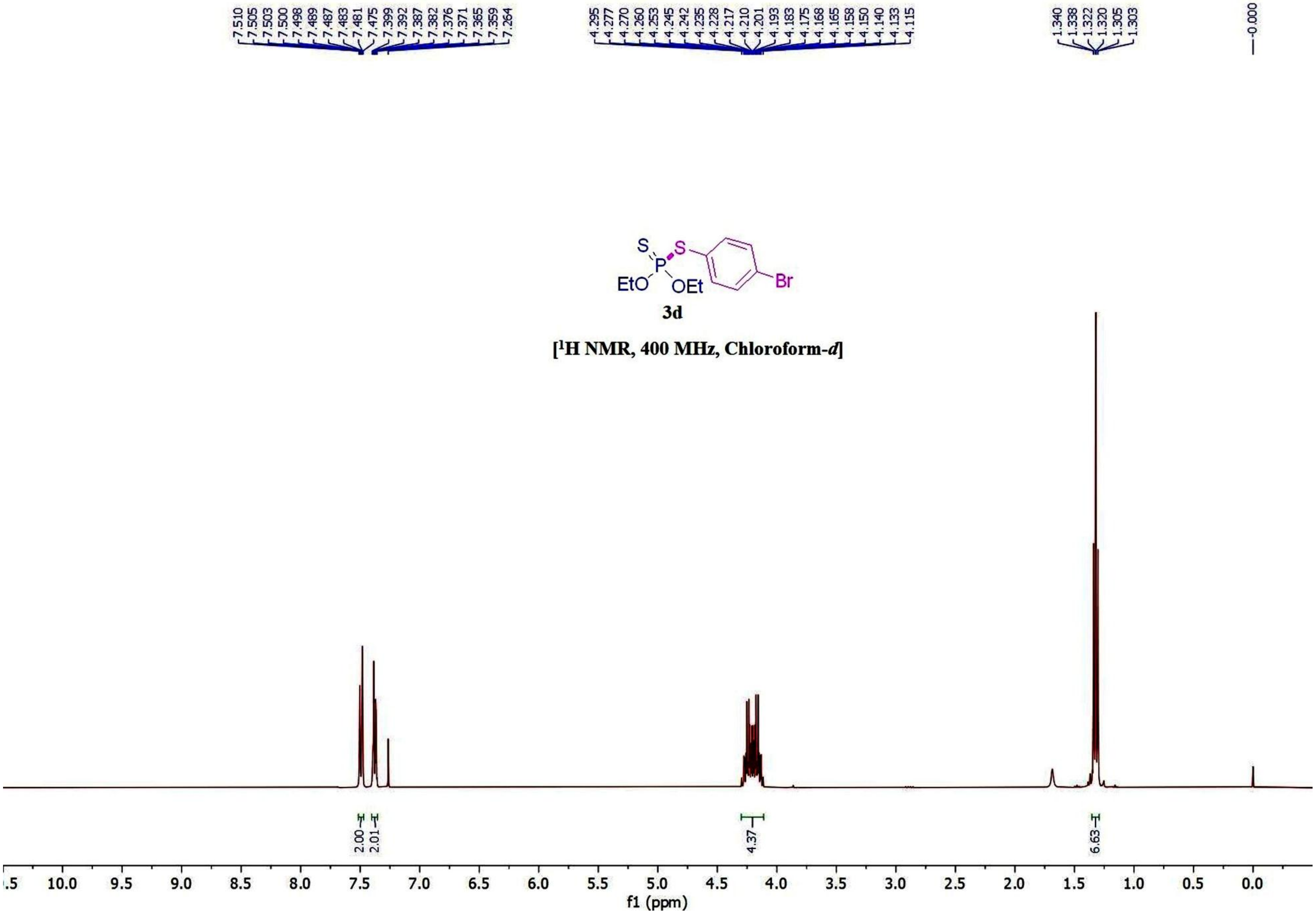

3d

[^{1}H NMR, 400 MHz, Chloroform-*d*]

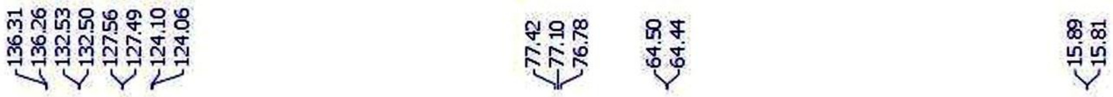

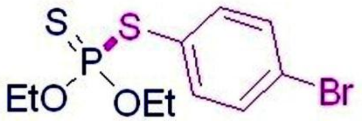

3d

[^{13}C{H} NMR, 100 MHz, Chloroform-*d*]

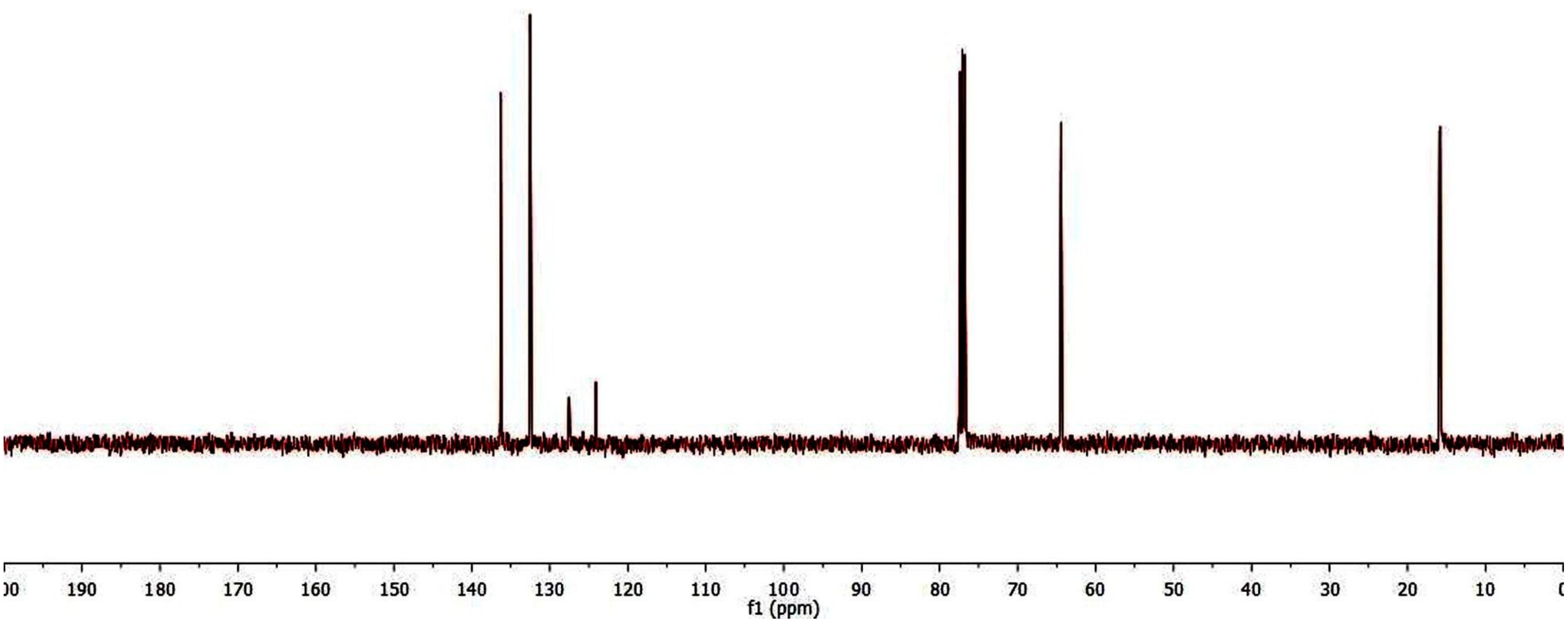

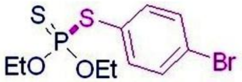

3d

[^{31}P NMR, 162 MHz, Chloroform-*d*]

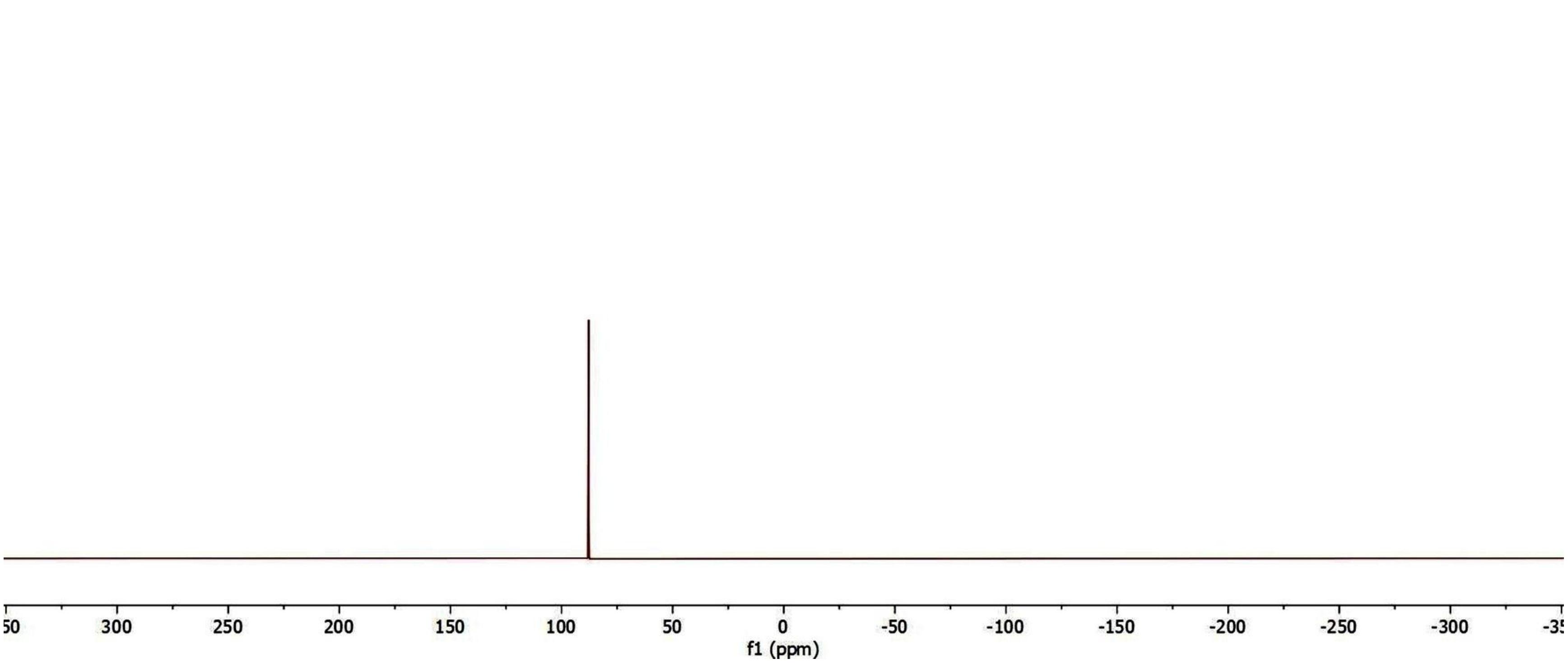

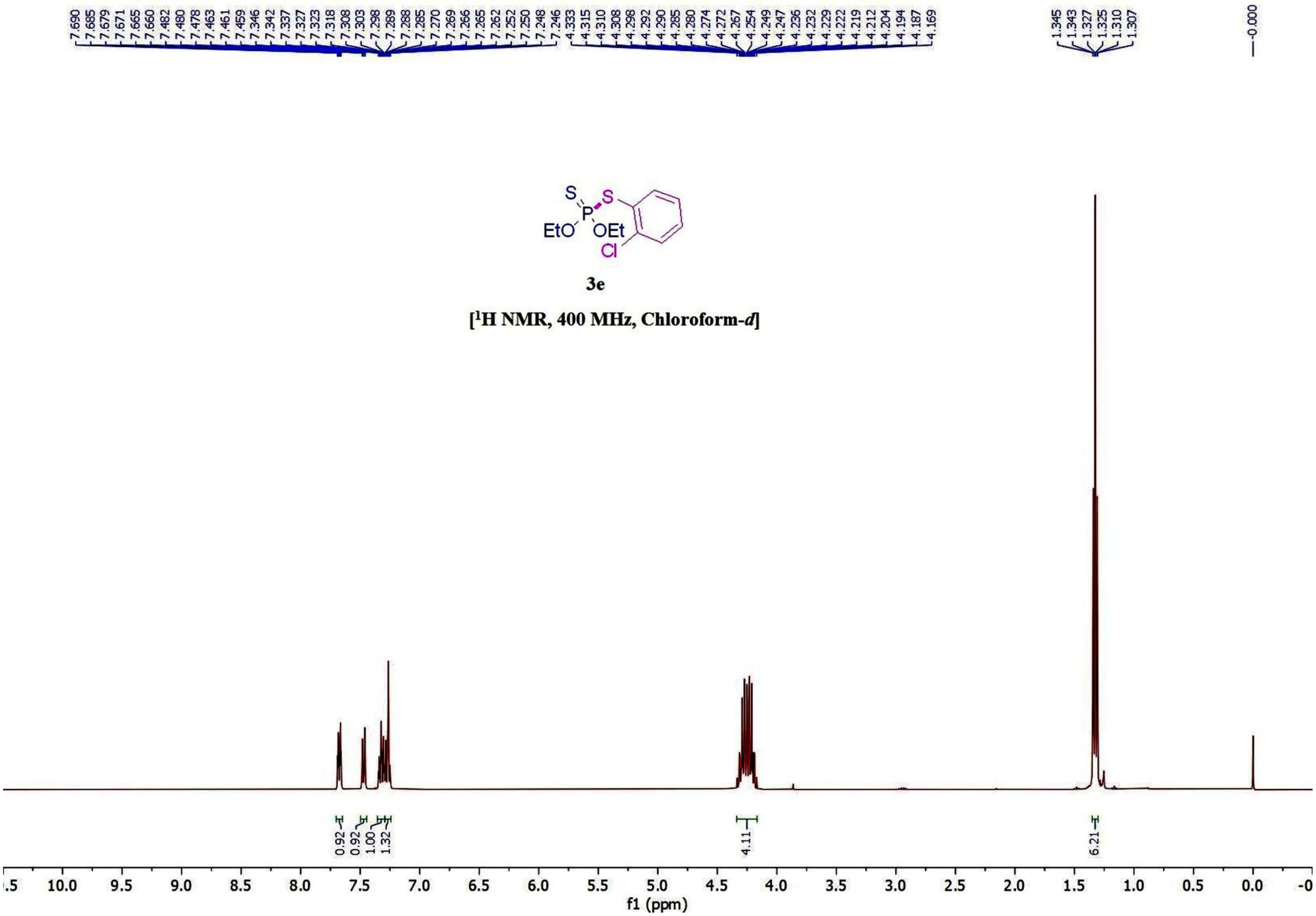

3e

[¹H NMR, 400 MHz, Chloroform-*d*]

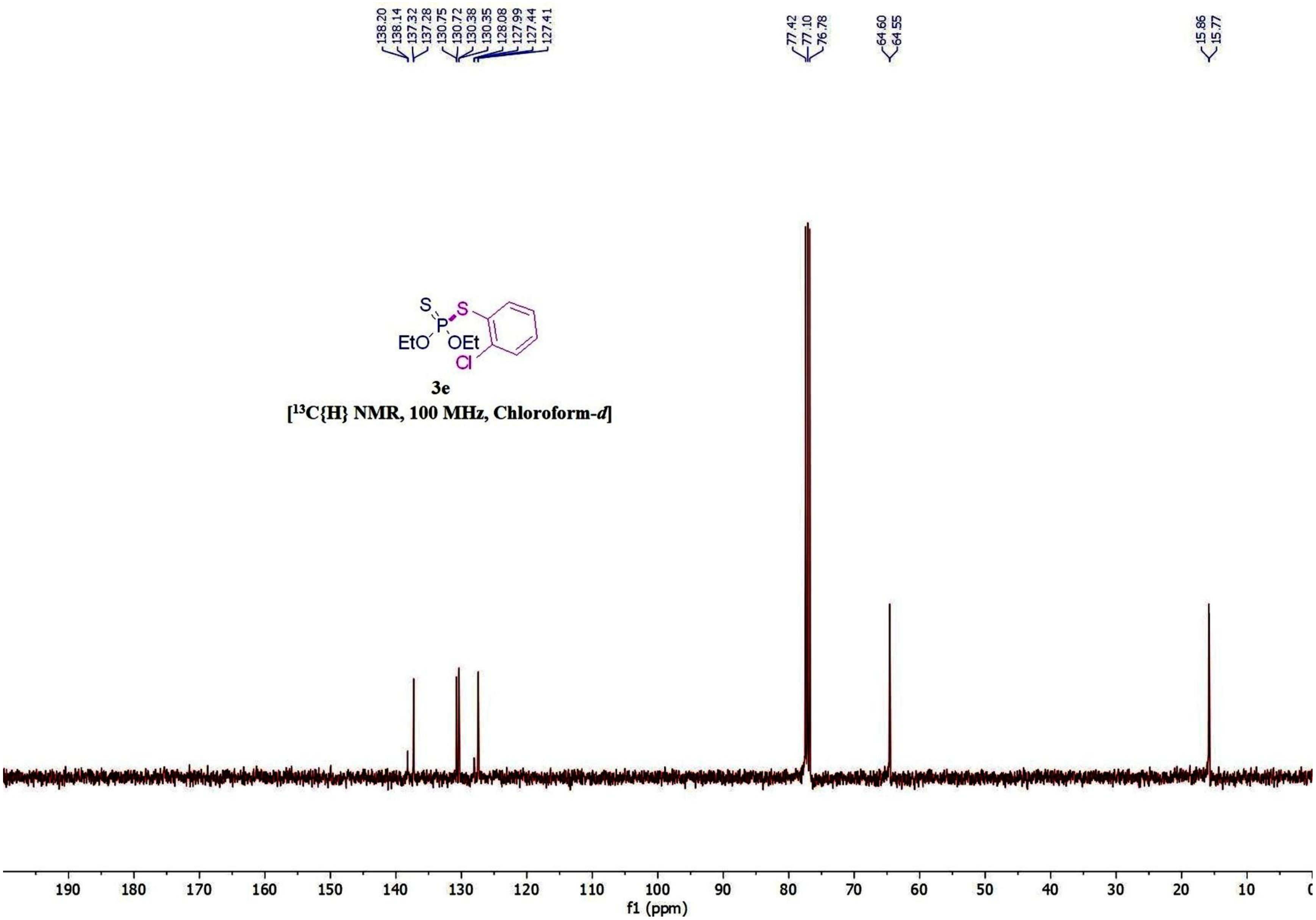
138.20
138.14
137.32
137.28
130.75
130.72
130.38
130.35
128.08
127.99
127.44
127.41
77.42
77.10
76.78
64.60
64.55
15.86
15.77
S
S
P
EtO
OEt
Cl
3e
[13C{H} NMR, 100 MHz, Chloroform-d]
190
180
170
160
150
140
130
120
110
100
90
80
70
60
50
40
30
20
10
f1 (ppm)

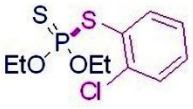

3e

[^{31}P NMR, 162 MHz, Chloroform-*d*]

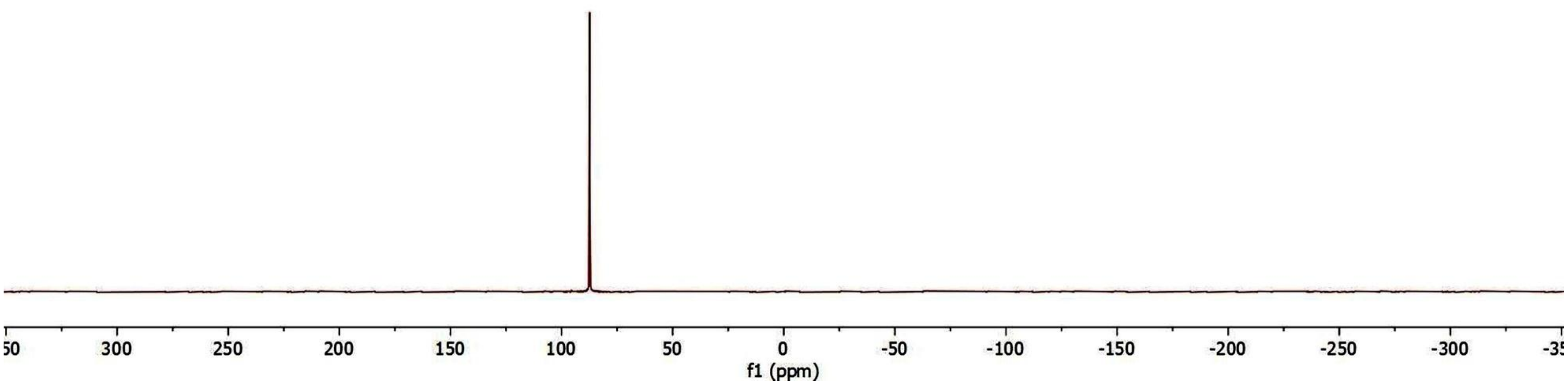

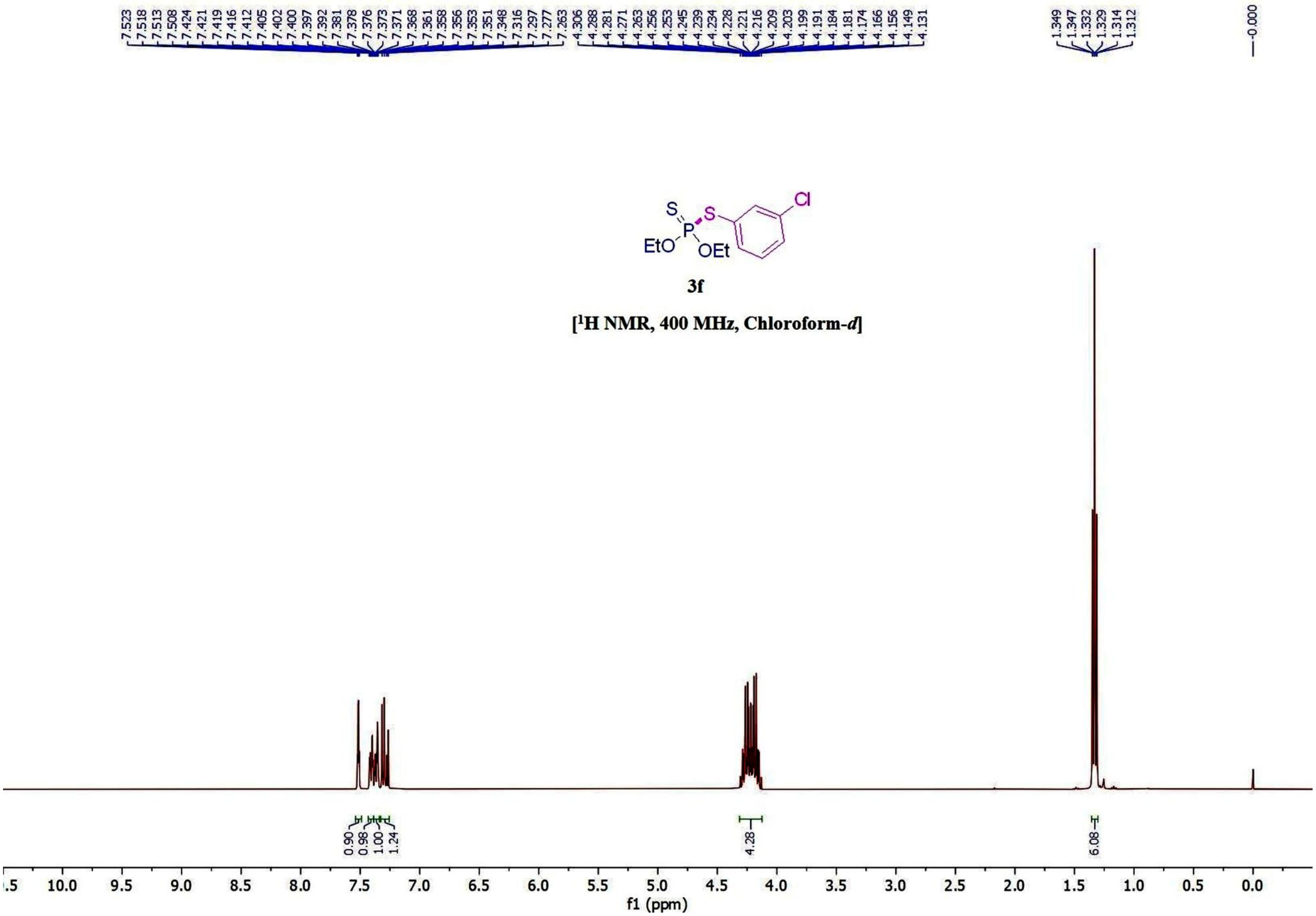
7.523 7.518 7.513 7.508 7.424 7.421 7.419 7.416 7.412 7.405 7.402 7.400 7.397 7.392 7.381 7.378 7.376 7.373 7.371 7.368 7.361 7.358 7.356 7.353 7.351 7.348 7.316 7.297 7.277 7.263 4.306 4.288 4.281 4.271 4.263 4.256 4.253 4.245 4.239 4.234 4.228 4.221 4.216 4.209 4.203 4.199 4.191 4.184 4.181 4.174 4.166 4.156 4.149 4.131
1.349 1.347 1.332 1.329 1.314 1.312
0.000
S
S
Cl
P
EtO
OEt
3f
[1H NMR, 400 MHz, Chloroform-d]
0.90 0.98 1.00 1.24
4.28
6.08
.5 10.0 9.5 9.0 8.5 8.0 7.5 7.0 6.5 6.0 5.5 5.0 4.5 4.0 3.5 3.0 2.5 2.0 1.5 1.0 0.5 0.0
f1 (ppm)

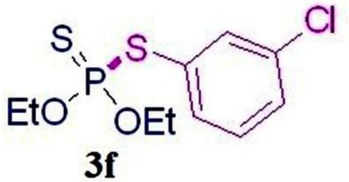

3f

[^{13}C{H} NMR, 100 MHz, Chloroform-*d*]

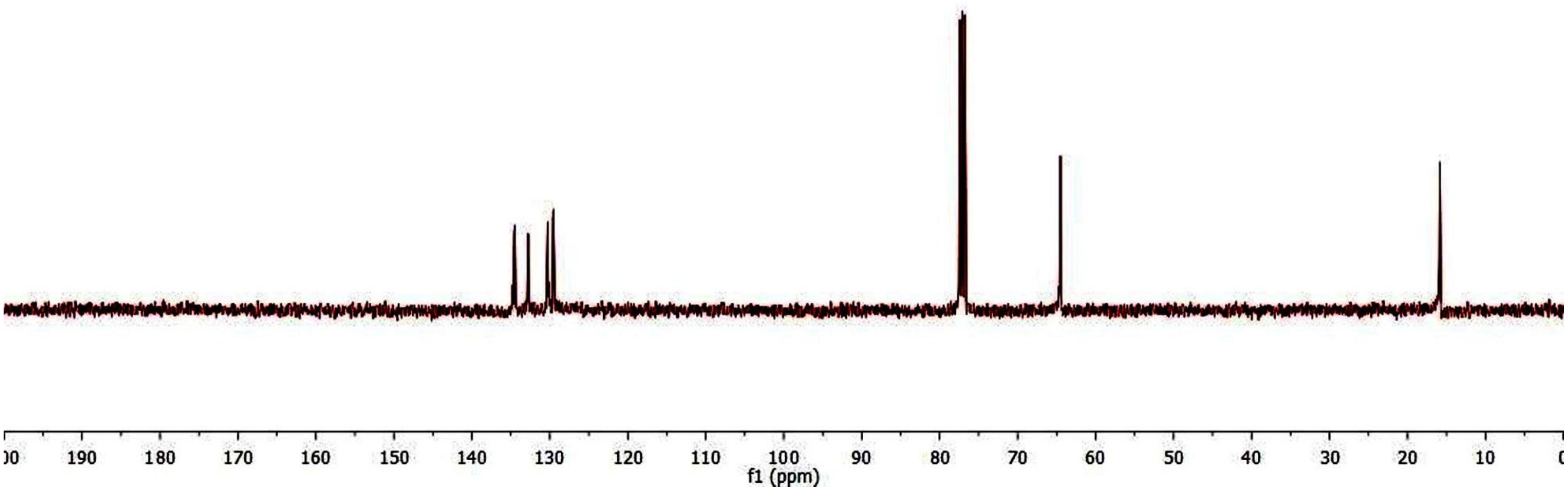

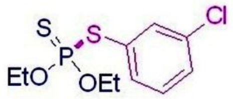

3f

[^{31}P NMR, 162 MHz, Chloroform-*d*]

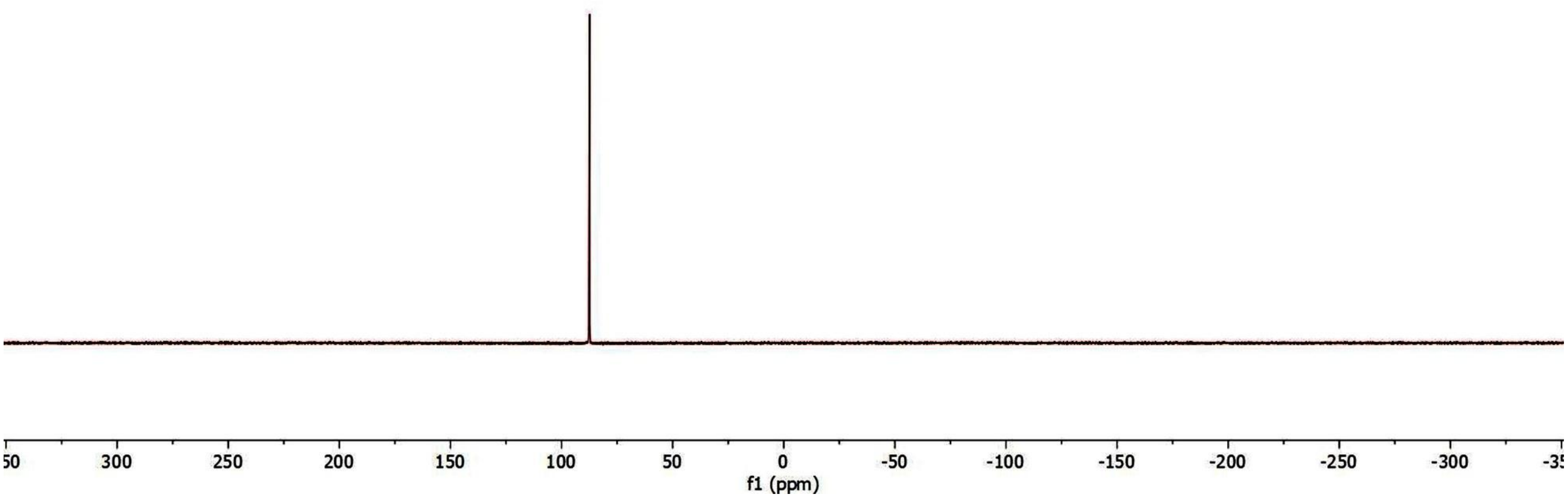

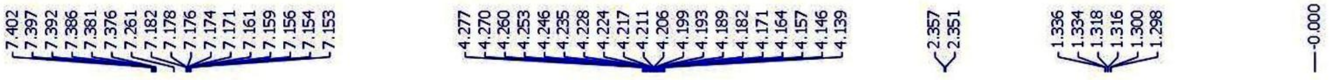

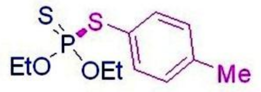

3g

[¹H NMR, 400 MHz, Chloroform-*d*]

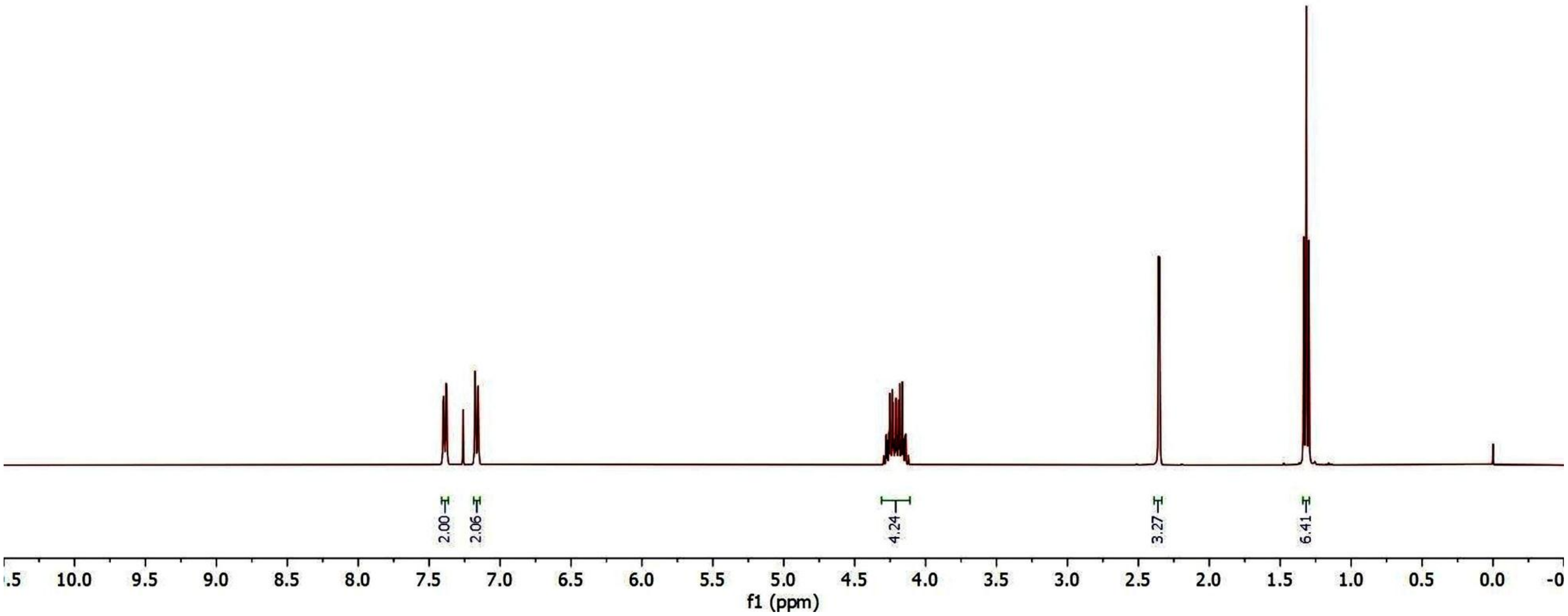

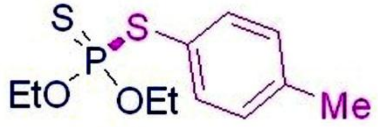

3g

[^{13}C{H} NMR, 100 MHz, Chloroform-*d*]

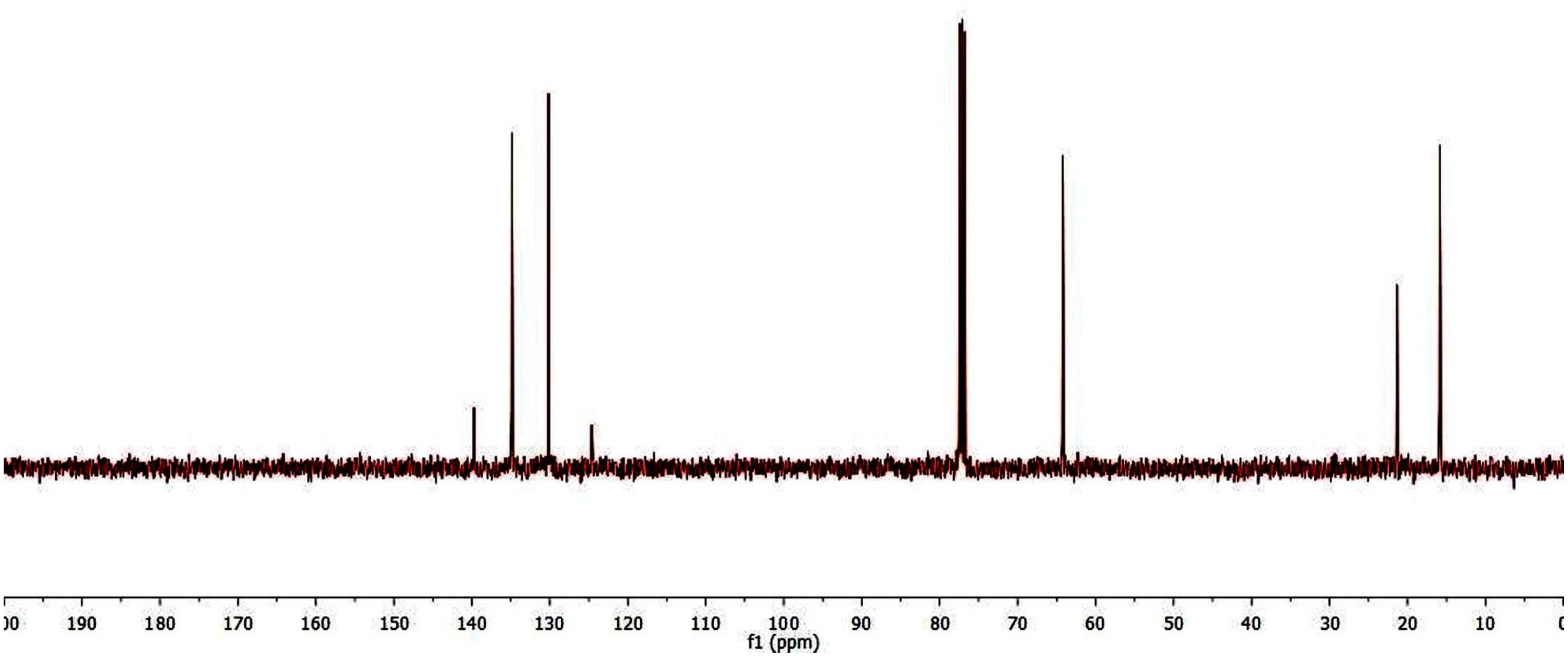

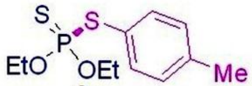

3g

[^{31}P NMR, 162 MHz, Chloroform-*d*]

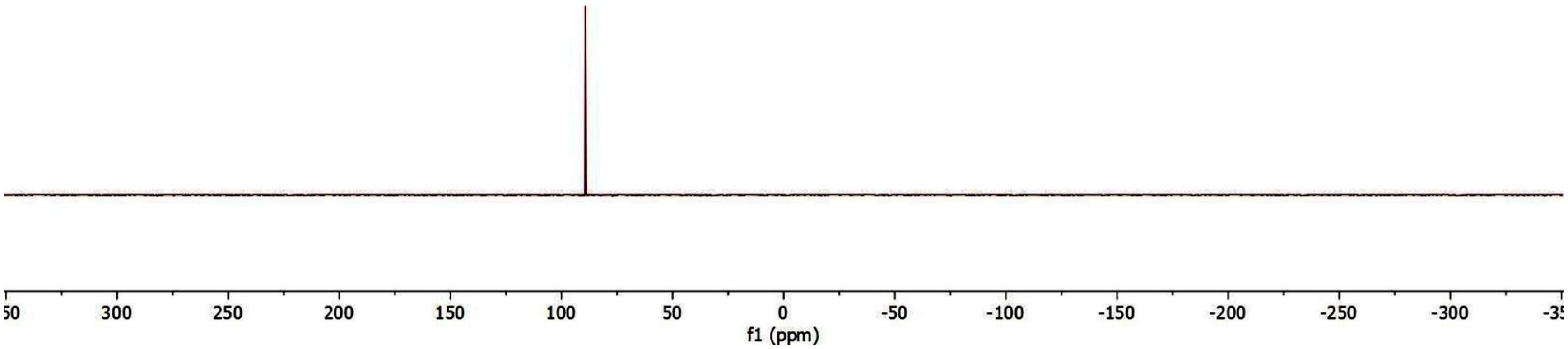

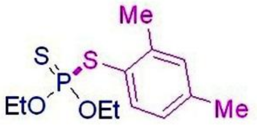

3h

[^{1}H NMR, 400 MHz, Chloroform-*d*]

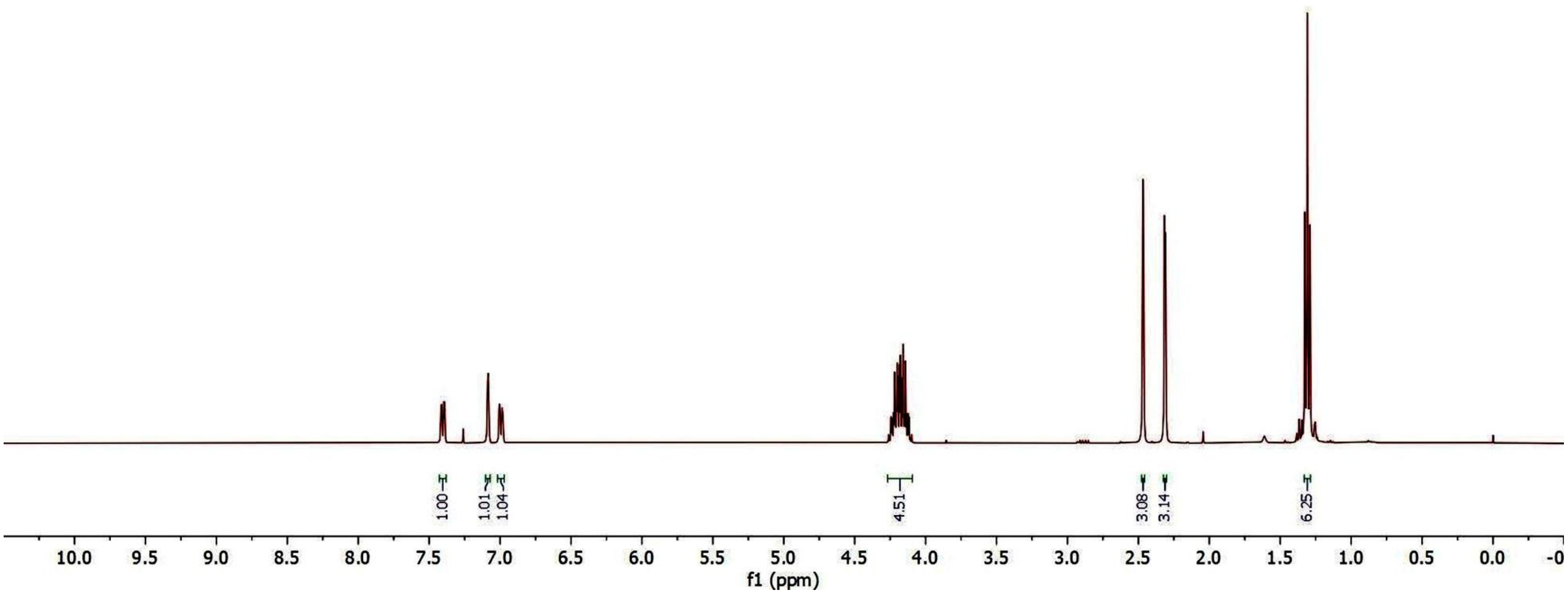

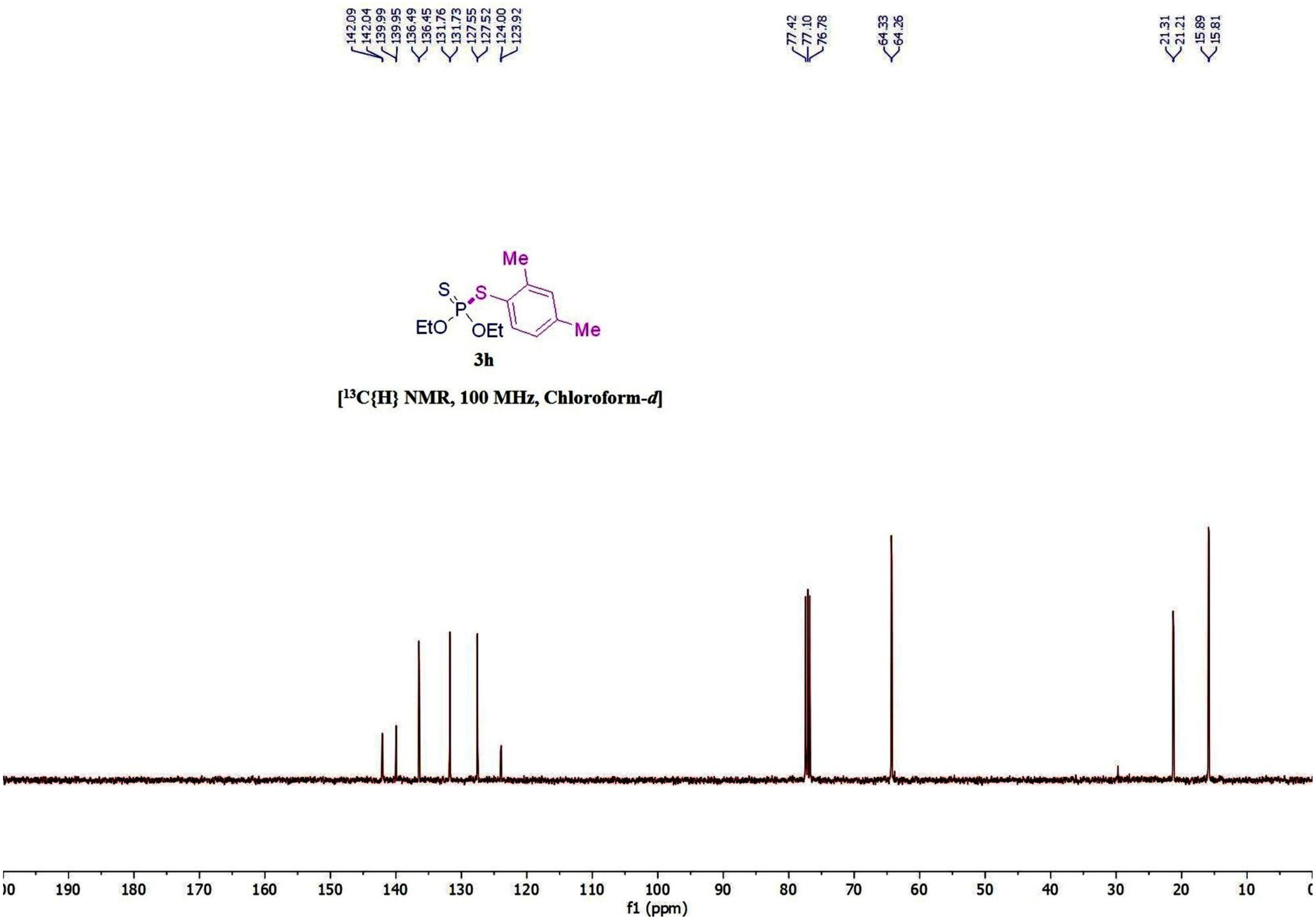

3h

[^{13}C{H} NMR, 100 MHz, Chloroform-*d*]

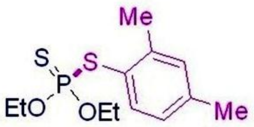

3h

[^{31}P NMR, 162 MHz, Chloroform-*d*]

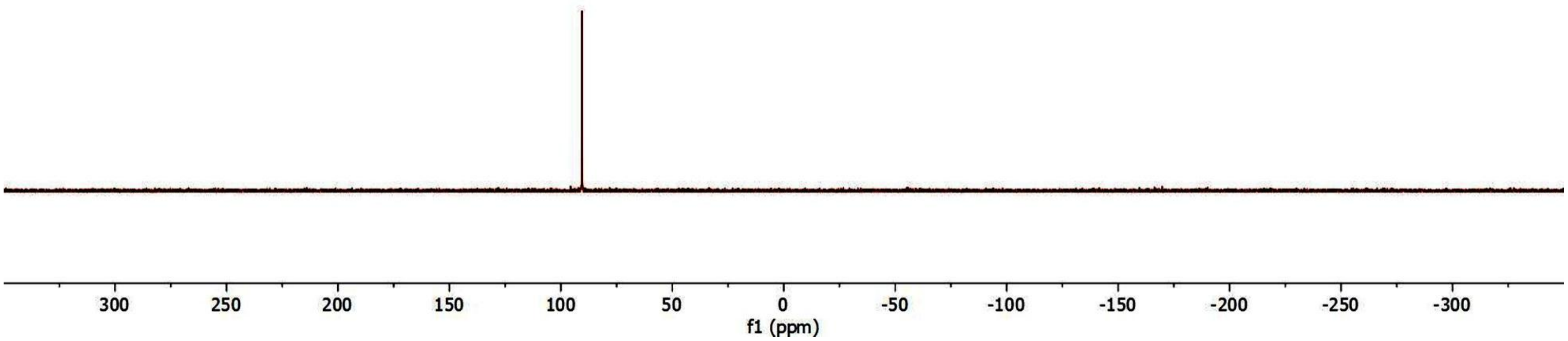

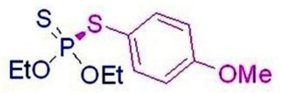

3i

[^{1}H NMR, 400 MHz, Chloroform-*d*]

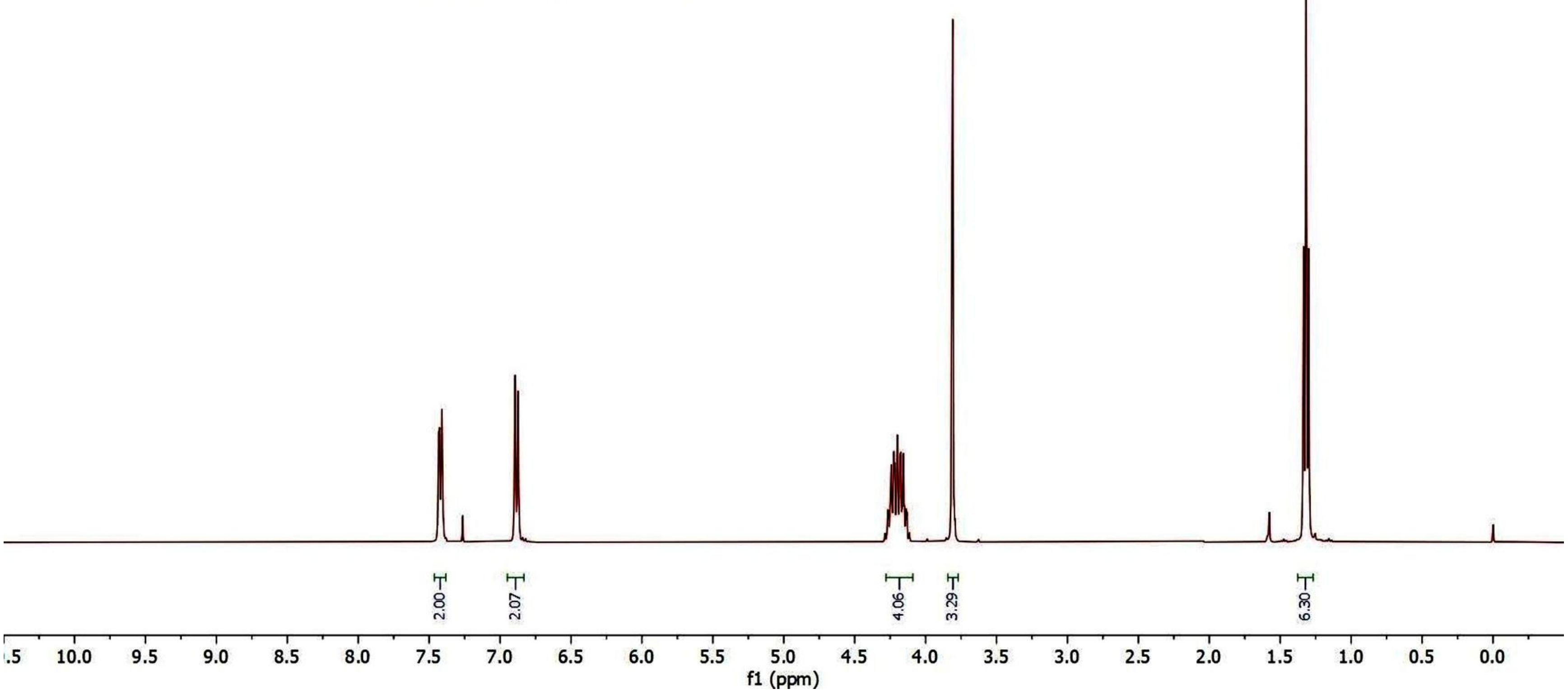

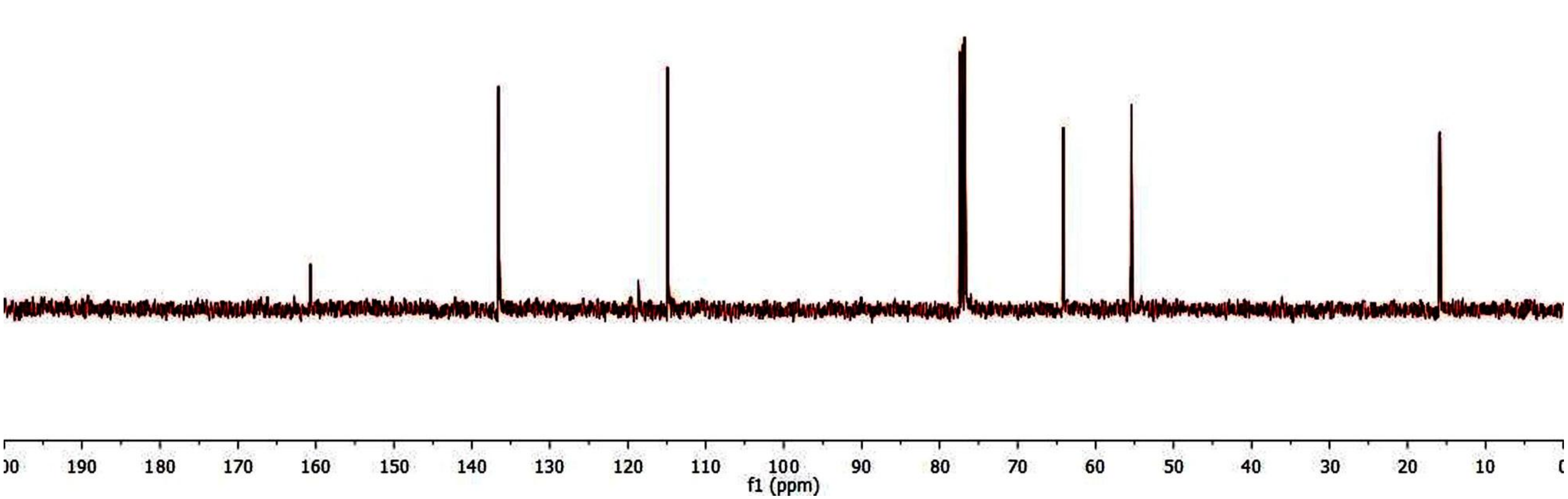

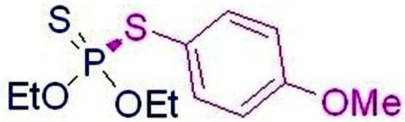

3i

[^{13}C{H} NMR, 100 MHz, Chloroform-*d*]

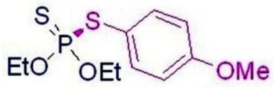

3i

[^{31}P NMR, 162 MHz, Chloroform-*d*]

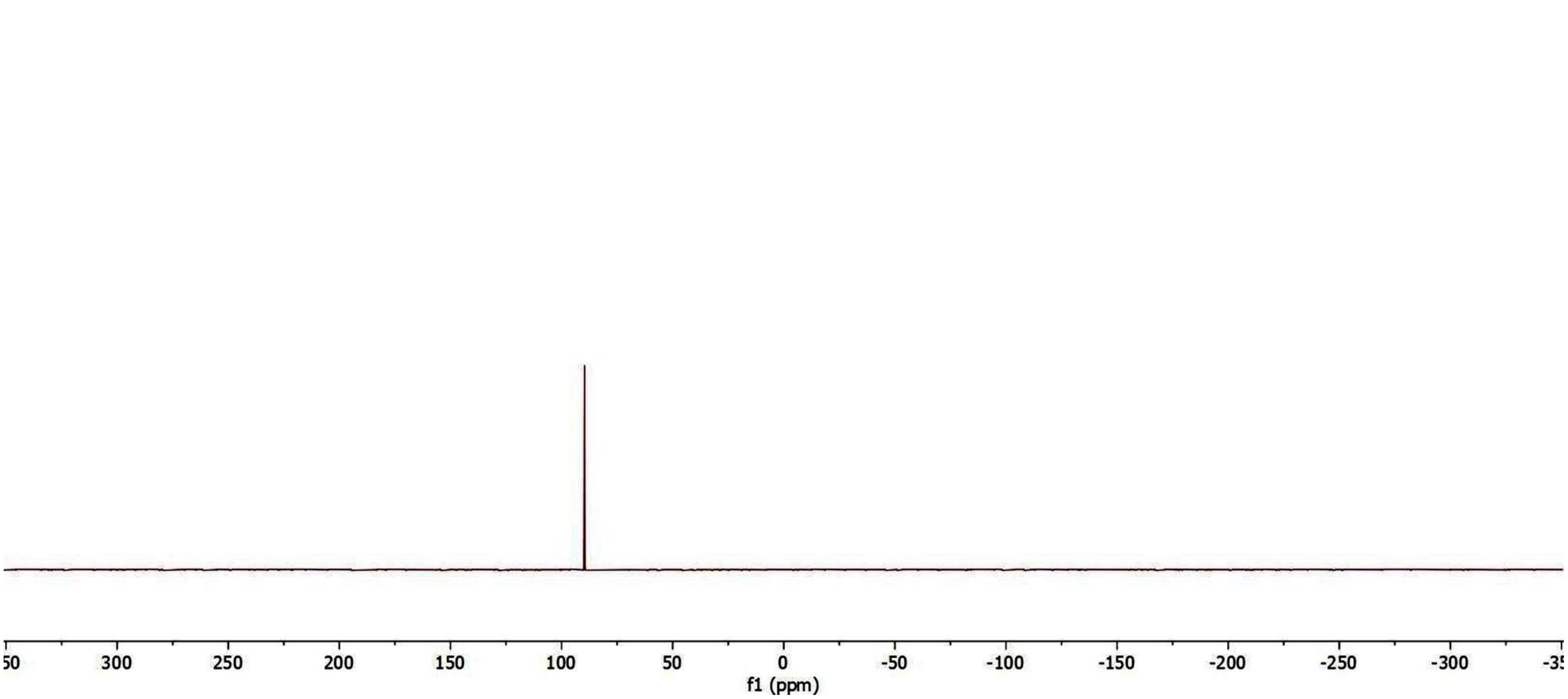

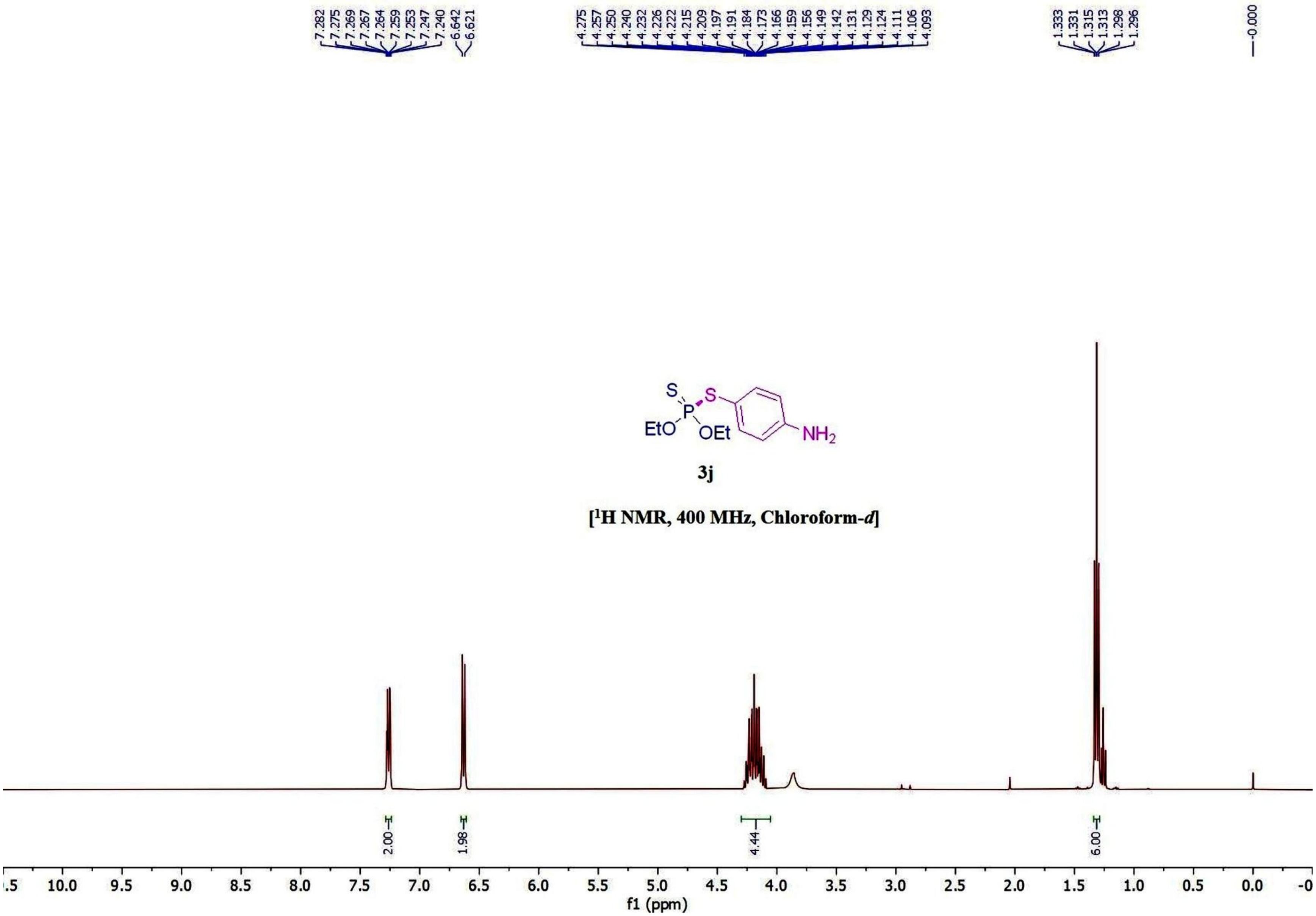

7.282
7.275
7.269
7.267
7.264
7.259
7.253
7.247
7.240
6.642
6.621
4.275
4.257
4.250
4.240
4.232
4.226
4.222
4.215
4.209
4.197
4.191
4.184
4.173
4.166
4.159
4.156
4.149
4.142
4.131
4.129
4.124
4.111
4.106
4.093
1.333
1.331
1.315
1.313
1.298
1.296
0.000
S
S
P
EtO
OEt
NH2
3j
[1H NMR, 400 MHz, Chloroform-d]
2.00
1.98
4.44
6.00
f1 (ppm)

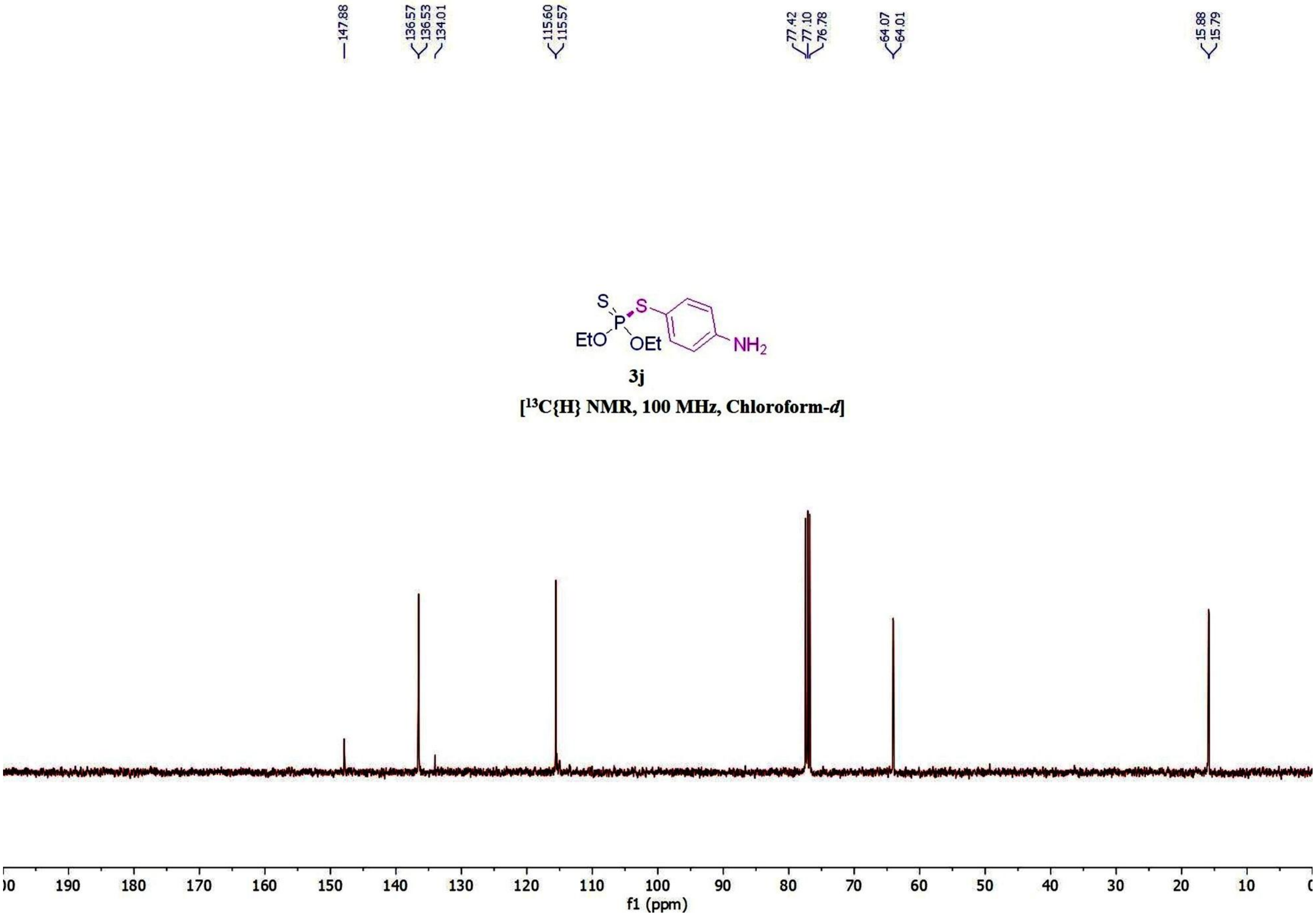

3j

[^{13}C{H} NMR, 100 MHz, Chloroform-*d*]

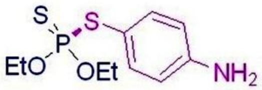

3j

[^{31}P NMR, 162 MHz, Chloroform-*d*]

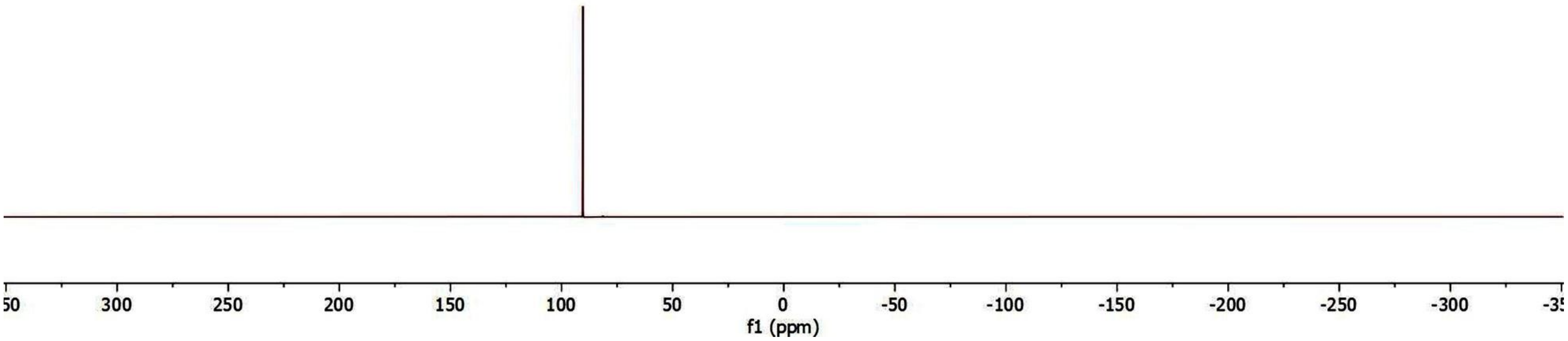

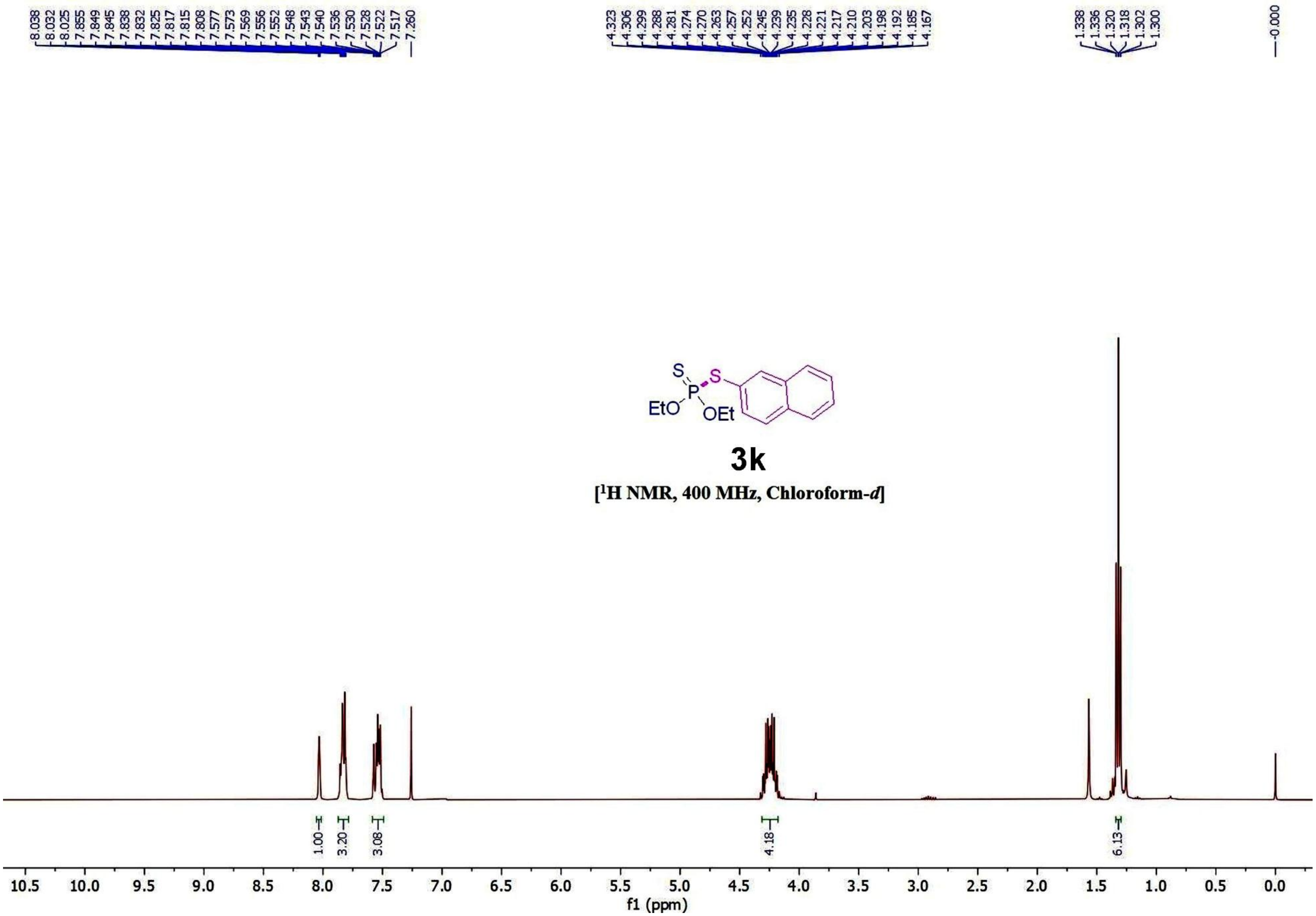

3k

[^{1}H NMR, 400 MHz, Chloroform-*d*]

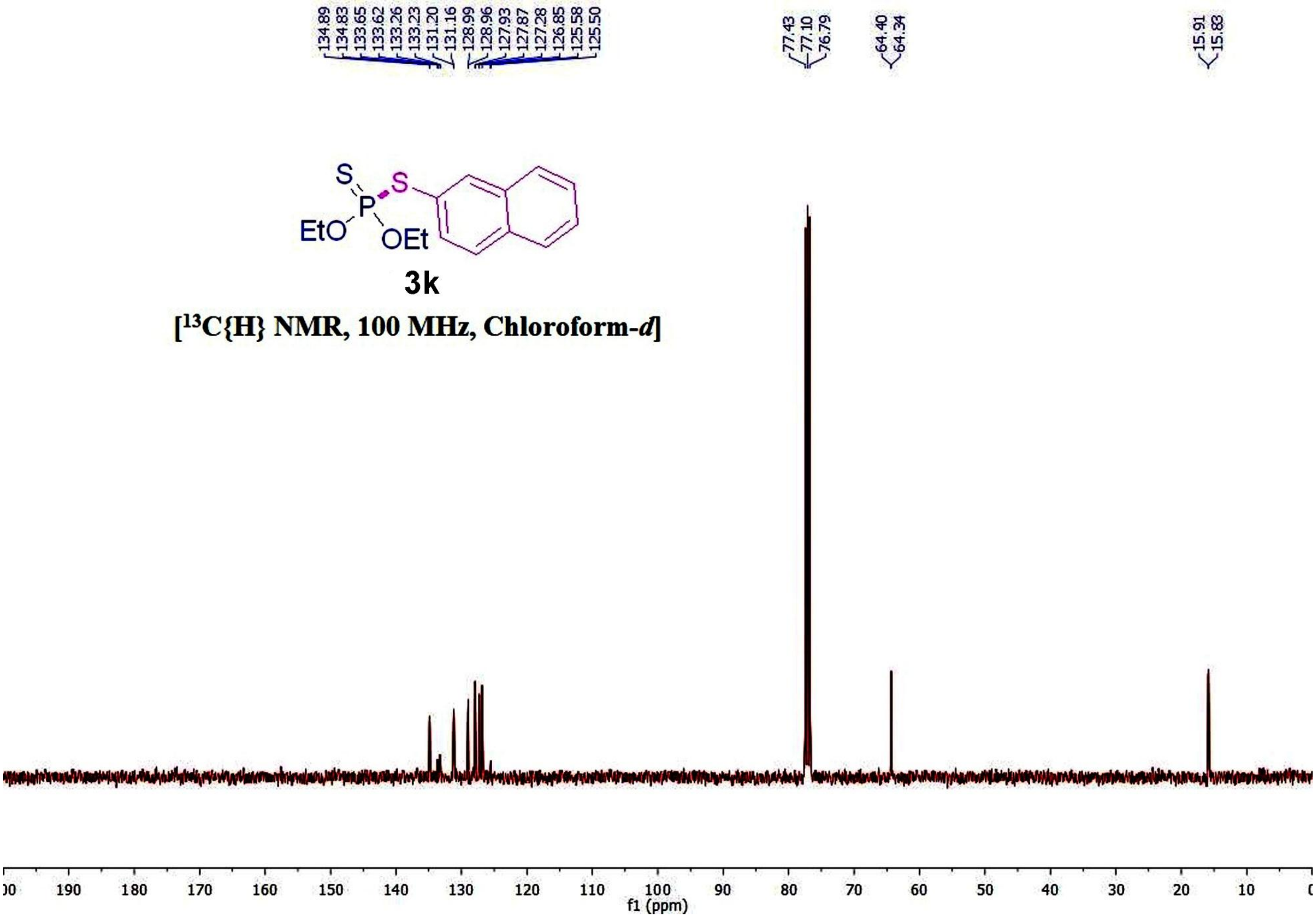

134.89
134.83
133.65
133.62
133.26
133.23
131.20
131.16
128.99
128.96
127.93
127.87
127.28
126.85
125.58
125.50
77.43
77.10
76.79
64.40
64.34
15.91
15.83
S
S
P
EtO
OEt
3k
[13C{H} NMR, 100 MHz, Chloroform-d]
190
180
170
160
150
140
130
120
110
100
90
80
70
60
50
40
30
20
10
f1 (ppm)

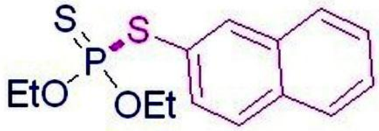

3k

[^{31}P NMR, 162 MHz, Chloroform-*d*]

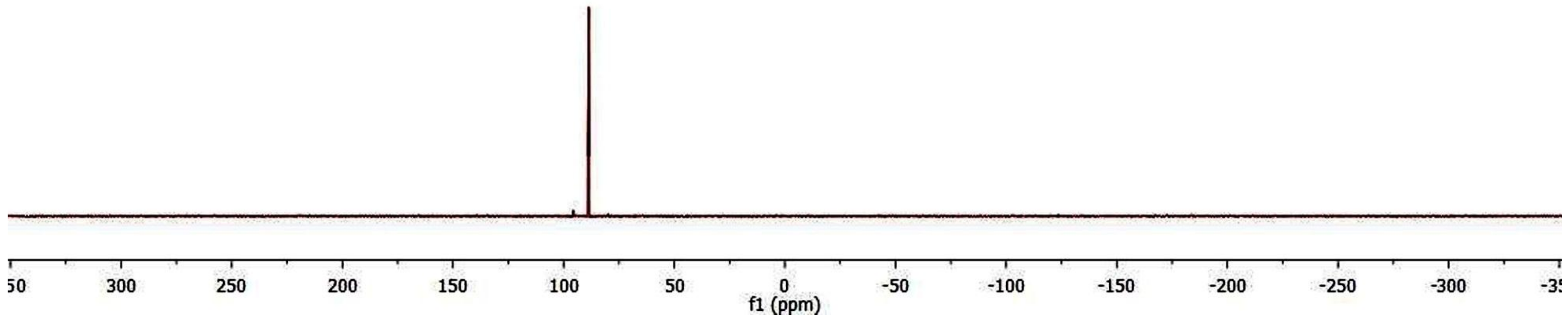

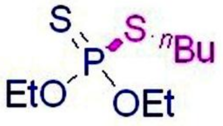

3l

[¹H NMR, 400 MHz, Chloroform-*d*]

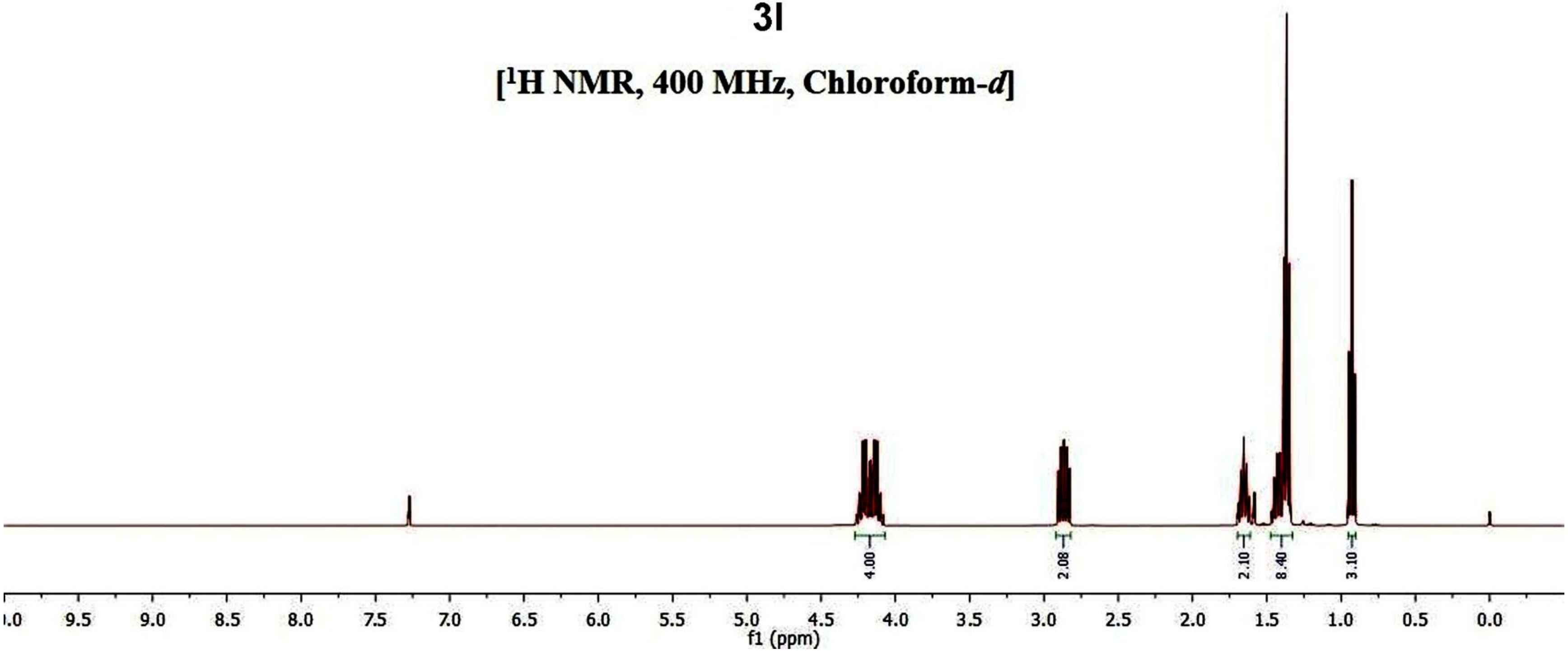

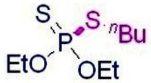

3l

[^{13}C{H} NMR, 100 MHz, Chloroform-*d*]

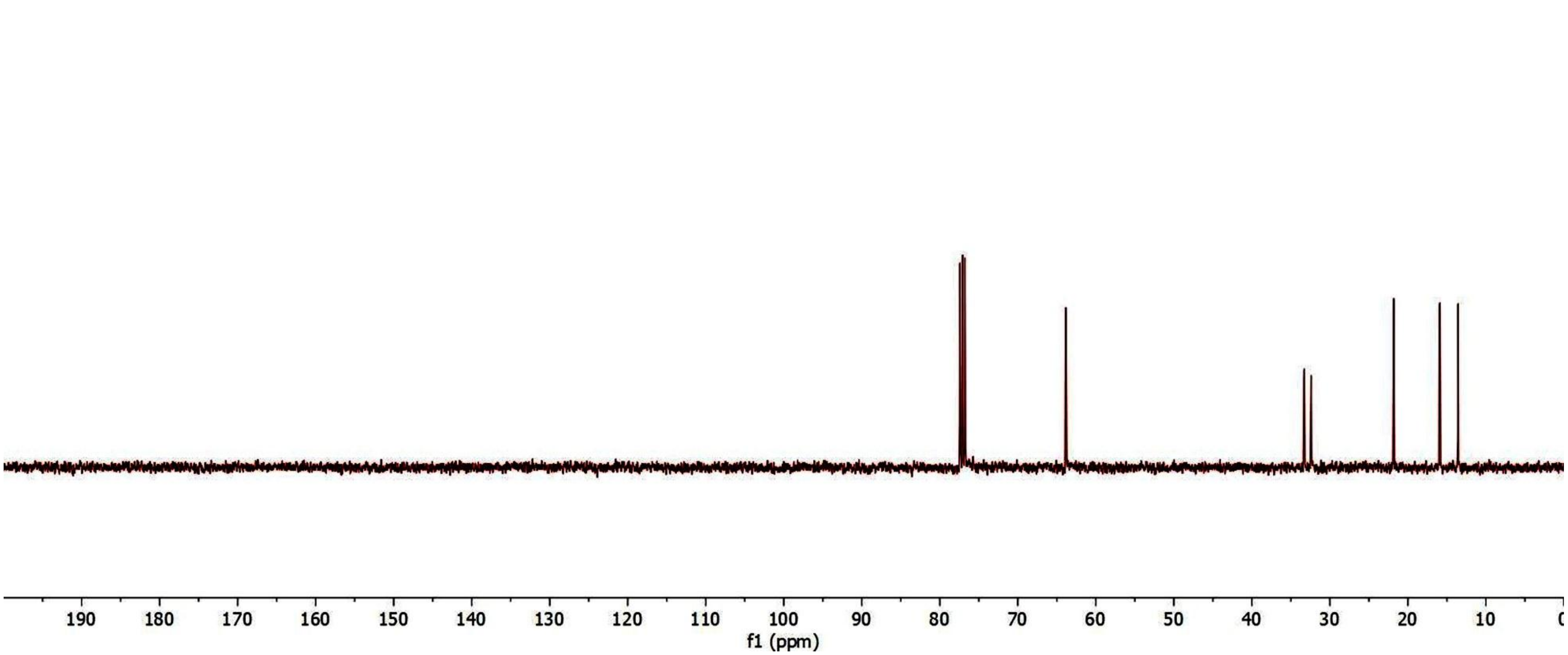

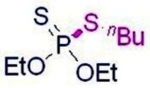

3l

[^{31}P NMR, 162 MHz, Chloroform-*d*]

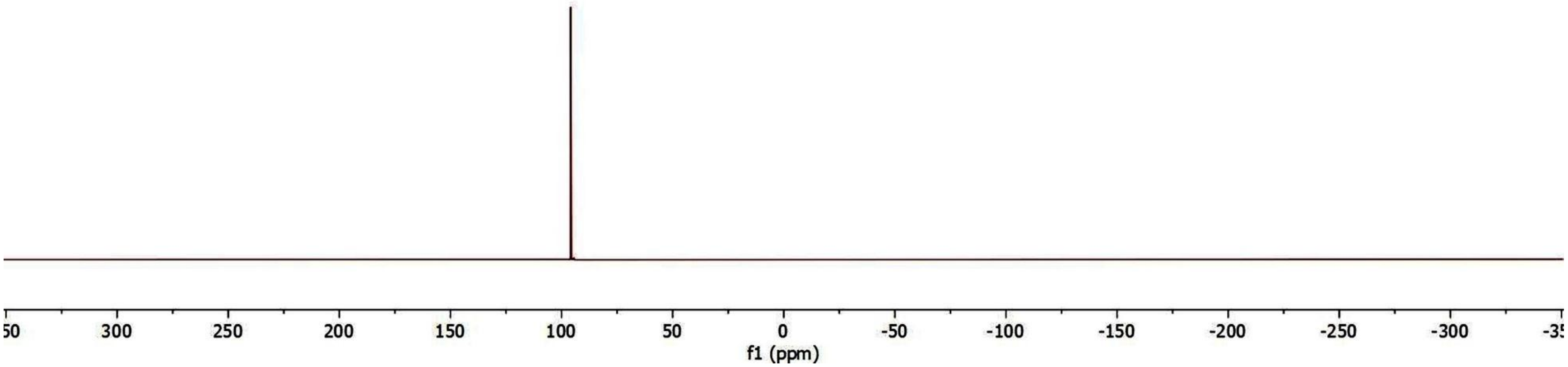

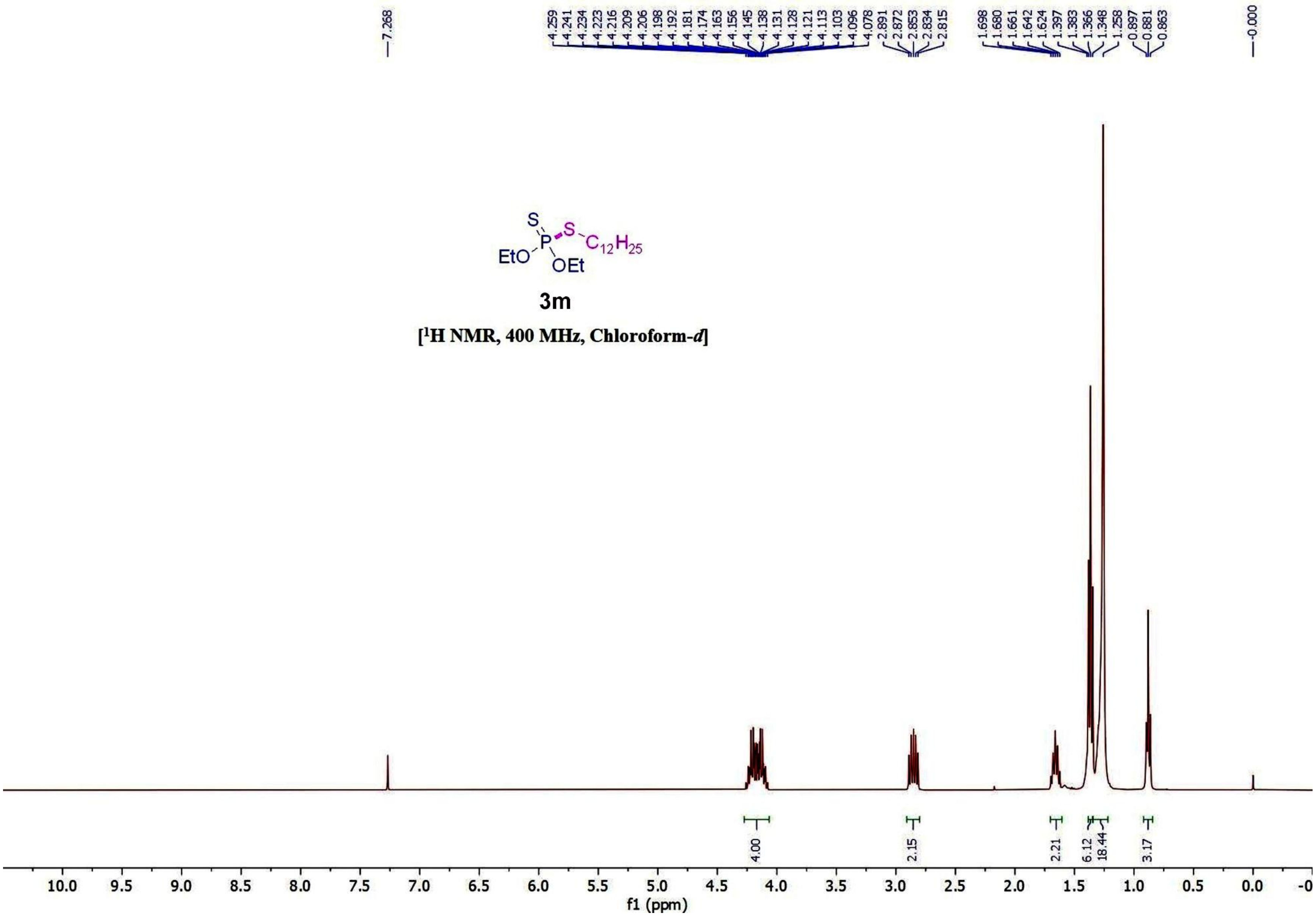

3m

[1H NMR, 400 MHz, Chloroform-*d*]

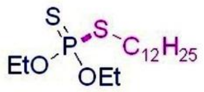

3m

[^{13}C{H} NMR, 100 MHz, Chloroform-*d*]

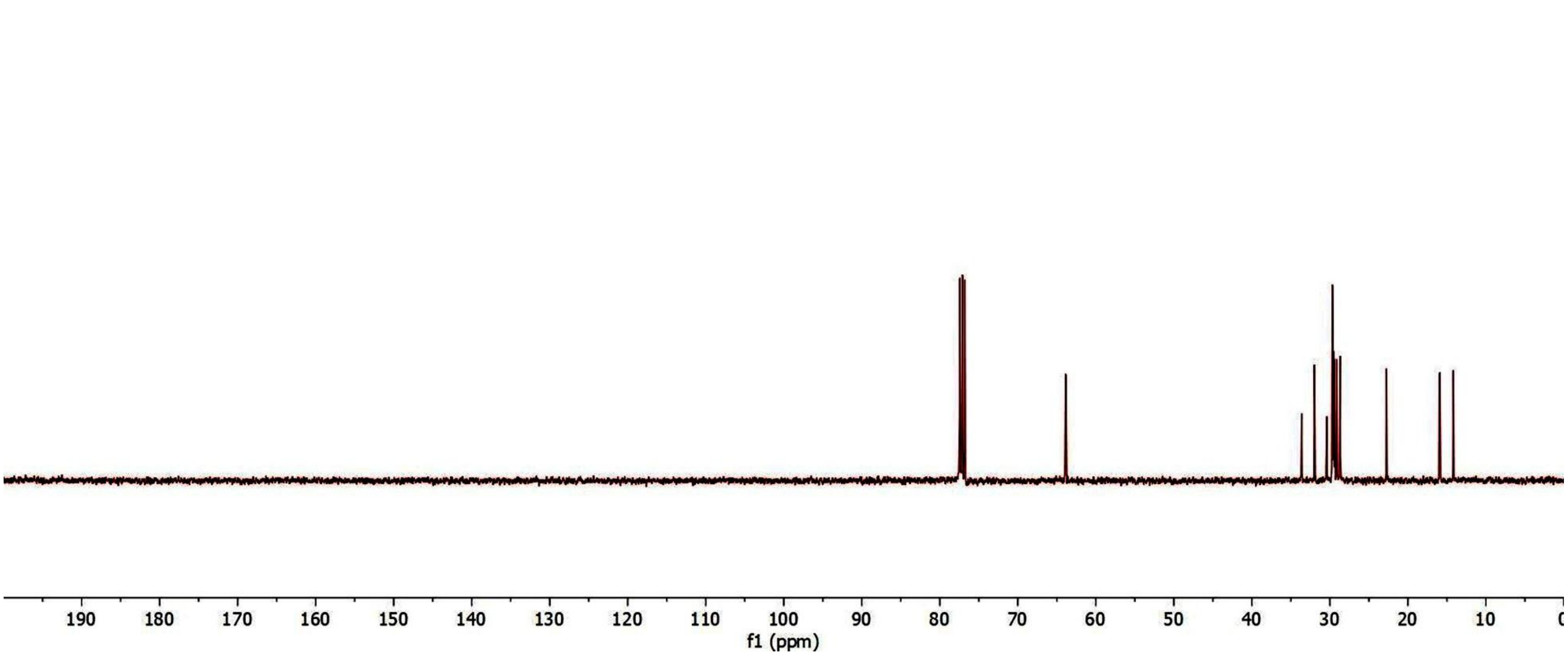

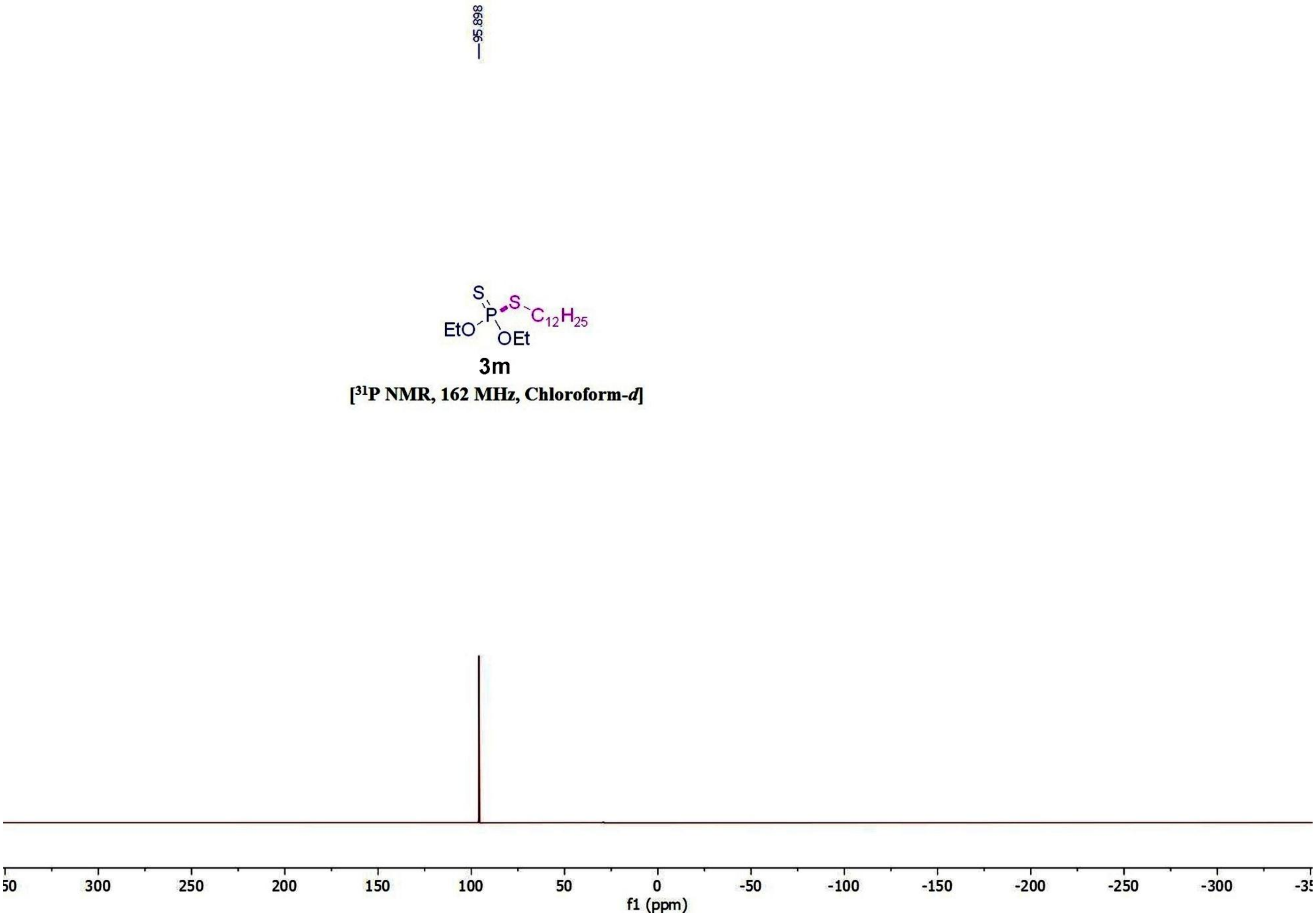

[^{31}P NMR, 162 MHz, Chloroform-*d*]

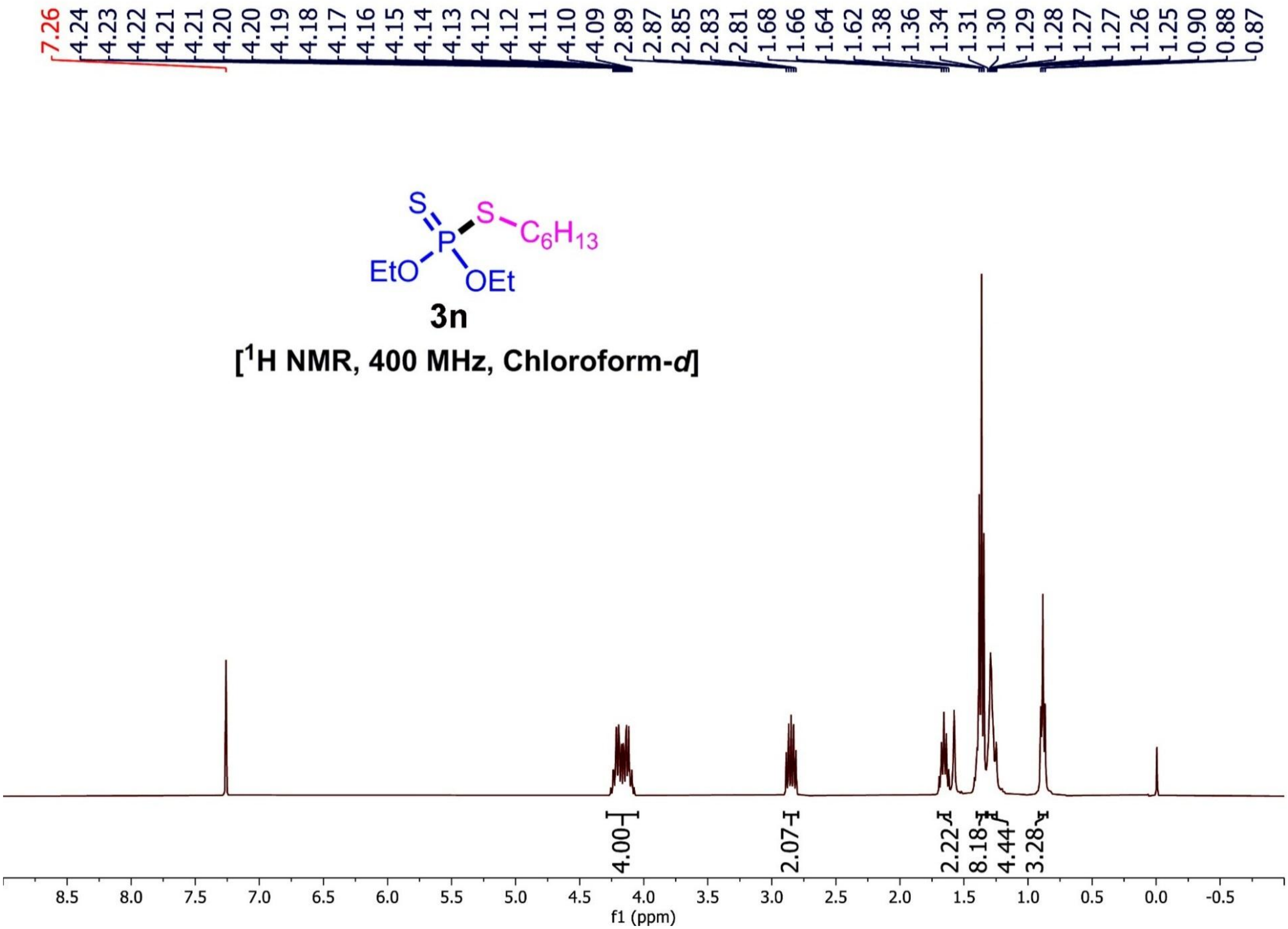

7.26
4.24
4.23
4.22
4.21
4.21
4.20
4.20
4.19
4.18
4.17
4.16
4.15
4.14
4.13
4.12
4.12
4.11
4.10
4.09
2.89
2.87
2.85
2.83
2.81
1.68
1.66
1.64
1.62
1.38
1.36
1.34
1.31
1.30
1.29
1.28
1.27
1.27
1.26
1.25
0.90
0.88
0.87
S
S
P
C6H13
EtO
OEt
3n
[1H NMR, 400 MHz, Chloroform-d]
4.00
2.07
2.22
8.18
4.44
3.28
8.5
8.0
7.5
7.0
6.5
6.0
5.5
5.0
4.5
4.0
3.5
3.0
2.5
2.0
1.5
1.0
0.5
0.0
-0.5
f1 (ppm)

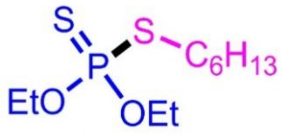

3n

[$^{13}C\{^{1}H\}$ NMR, 100 MHz, Chloroform-*d*]

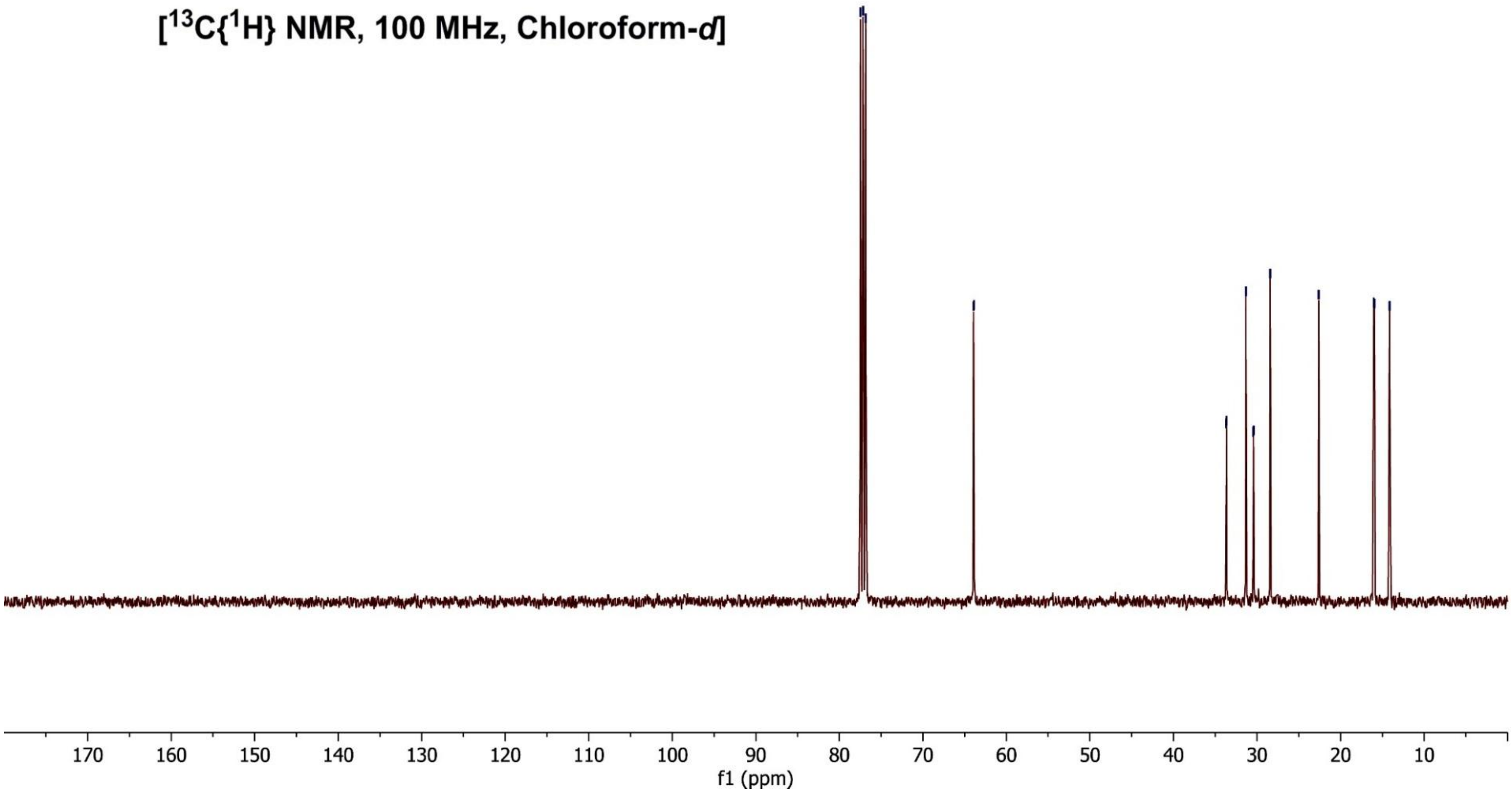

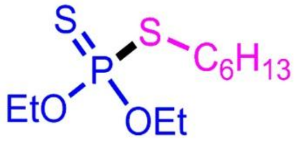

3n

[^{31}P NMR, 162 MHz, Chloroform-*d*]

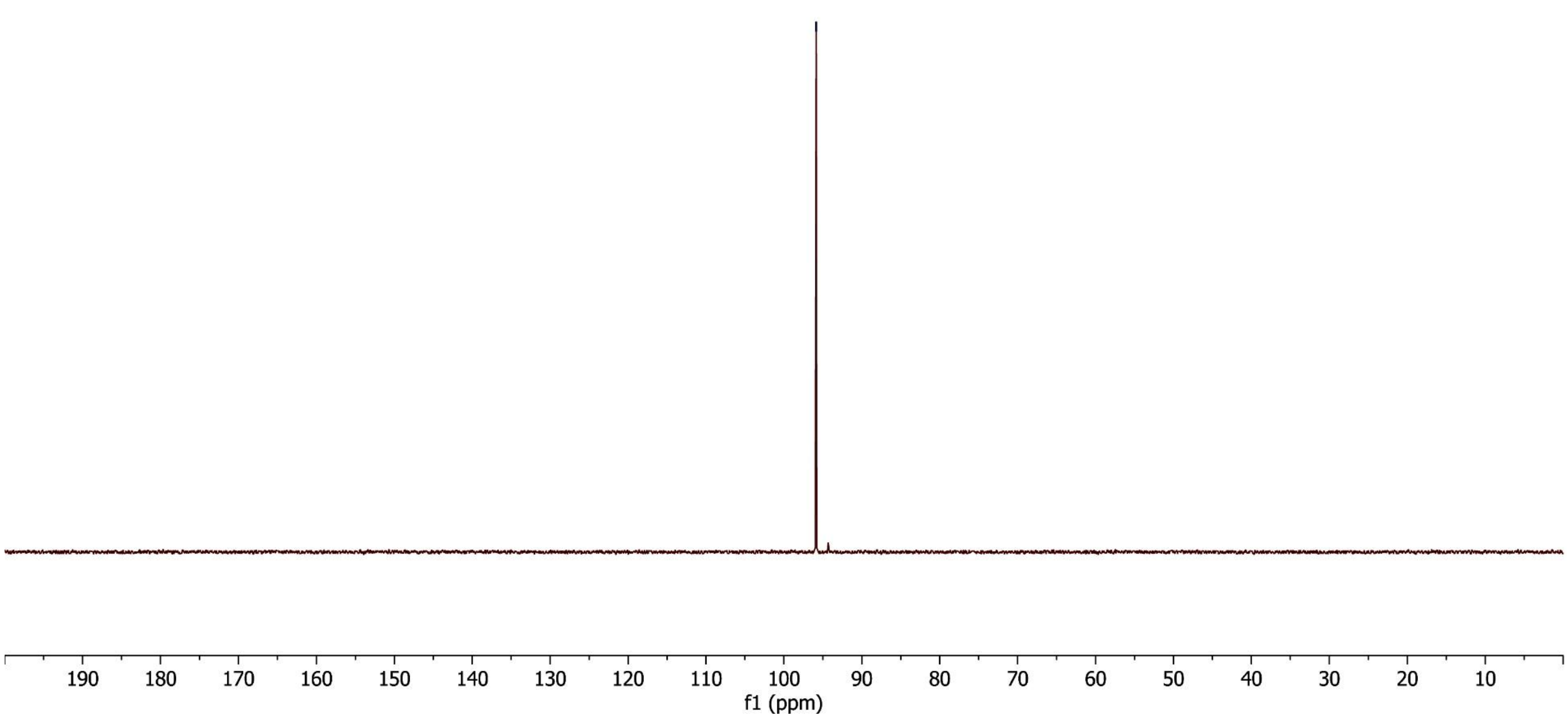

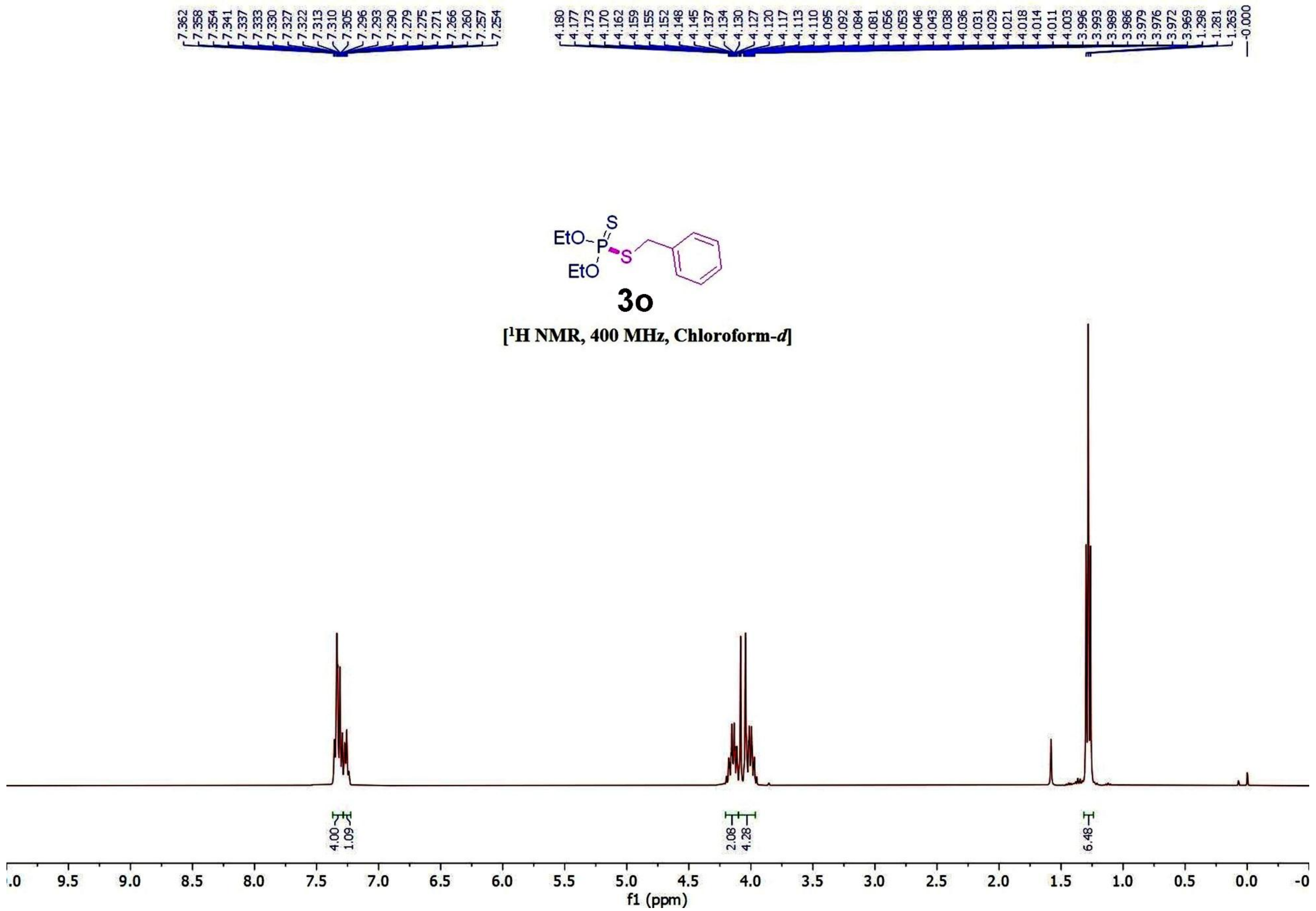
7.362 7.358 7.354 7.341 7.337 7.333 7.330 7.327 7.322 7.313 7.310 7.305 7.296 7.293 7.290 7.279 7.275 7.271 7.266 7.260 7.257 7.254
4.180 4.177 4.173 4.170 4.162 4.159 4.155 4.152 4.148 4.145 4.137 4.134 4.130 4.127 4.120 4.117 4.113 4.110 4.095 4.092 4.084 4.081 4.056 4.053 4.046 4.043 4.038 4.036 4.031 4.029 4.021 4.018 4.014 4.011 4.003 3.996 3.993 3.989 3.986 3.979 3.976 3.972 3.969 1.298 1.281 1.263 0.000
EtO
EtO
P
S
S
3o
[1H NMR, 400 MHz, Chloroform-d]
4.00
1.09
2.08
4.28
6.48
9.5 9.0 8.5 8.0 7.5 7.0 6.5 6.0 5.5 5.0 4.5 4.0 3.5 3.0 2.5 2.0 1.5 1.0 0.5 0.0
f1 (ppm)

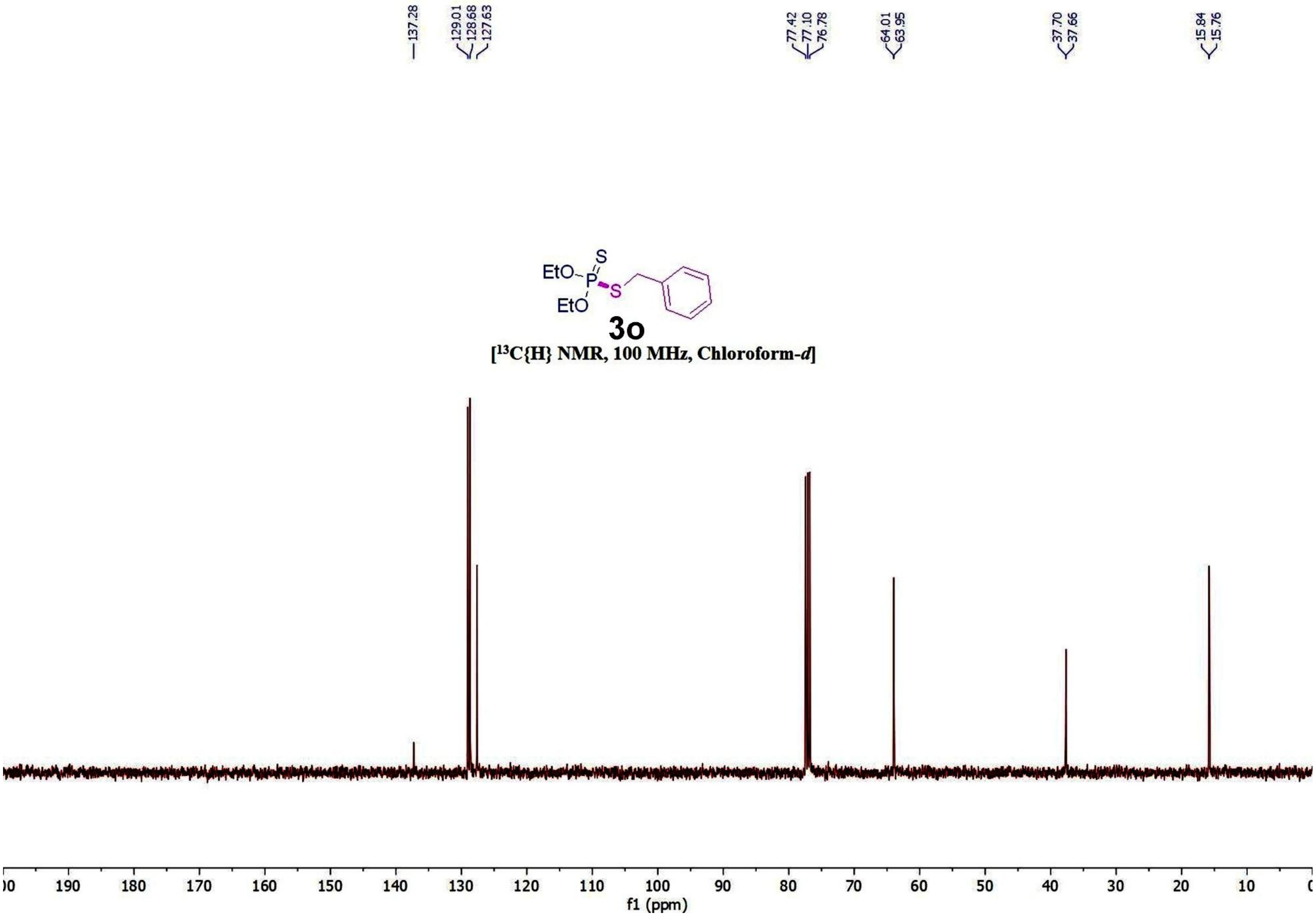
137.28
129.01
128.68
127.63
77.42
77.10
76.78
64.01
63.95
37.70
37.66
15.84
15.76
EtO
P
S
S
EtO
3o
[13C{H} NMR, 100 MHz, Chloroform-d]
190
180
170
160
150
140
130
120
110
100
90
80
70
60
50
40
30
20
10
f1 (ppm)

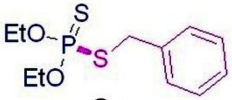

3o

[^{31}P NMR, 162 MHz, Chloroform-*d*]

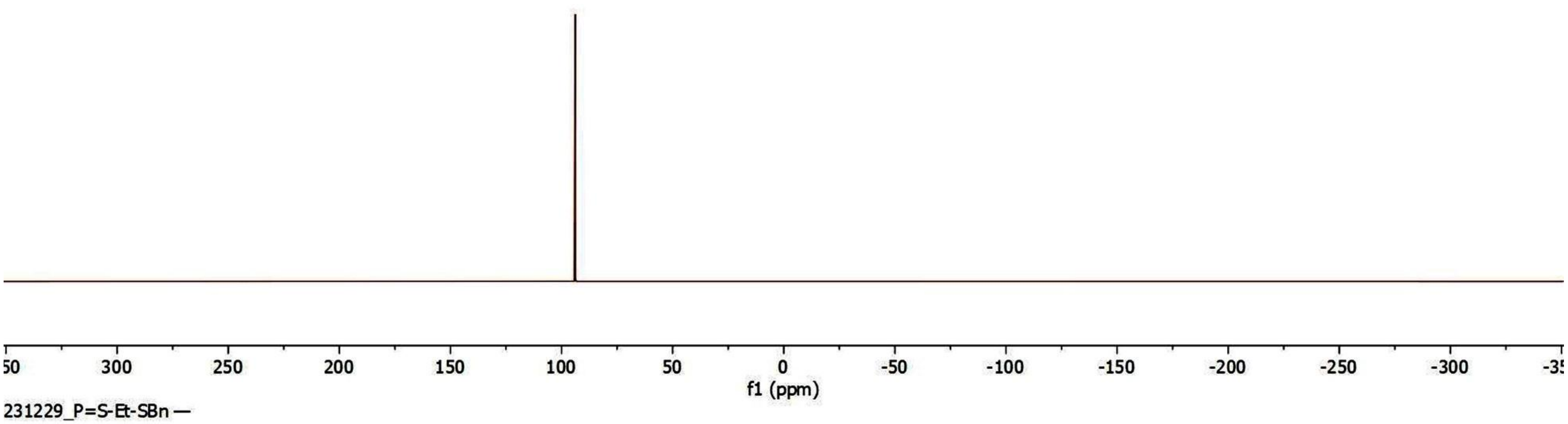

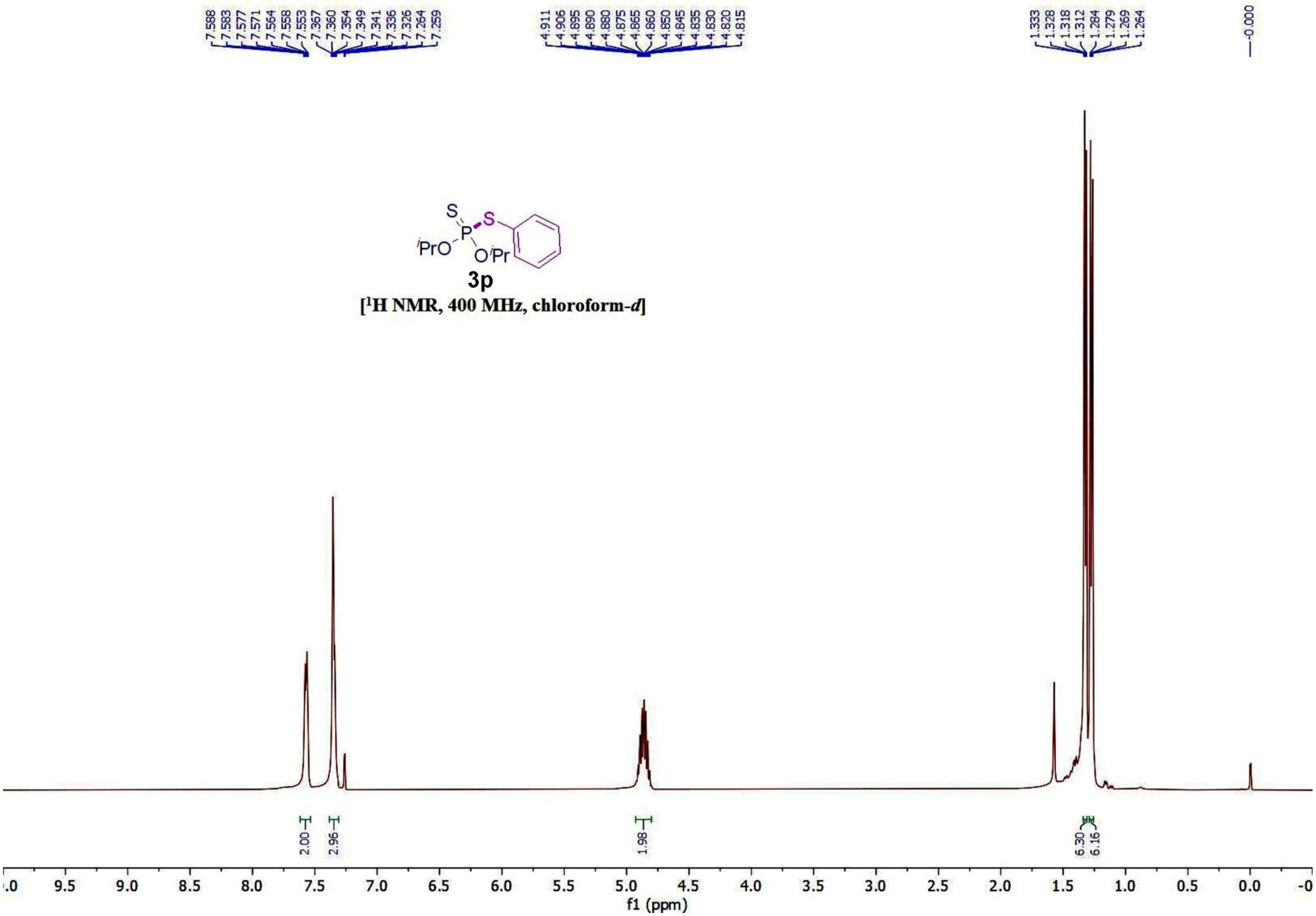

7.588
7.583
7.577
7.571
7.564
7.558
7.553
7.367
7.360
7.354
7.349
7.341
7.336
7.326
7.264
7.259
4.911
4.906
4.895
4.890
4.880
4.875
4.865
4.860
4.850
4.845
4.835
4.830
4.820
4.815
1.333
1.328
1.318
1.312
1.284
1.279
1.269
1.264
-0.000
S
S
P
iPrO
OiPr
3p
[1H NMR, 400 MHz, chloroform-d]
2.00
2.96
1.98
6.30
6.16
9.5
9.0
8.5
8.0
7.5
7.0
6.5
6.0
5.5
5.0
4.5
4.0
3.5
3.0
2.5
2.0
1.5
1.0
0.5
0.0
f1 (ppm)

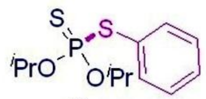

3p

[^{13}C{H} NMR, 100 MHz, Chloroform-*d*]

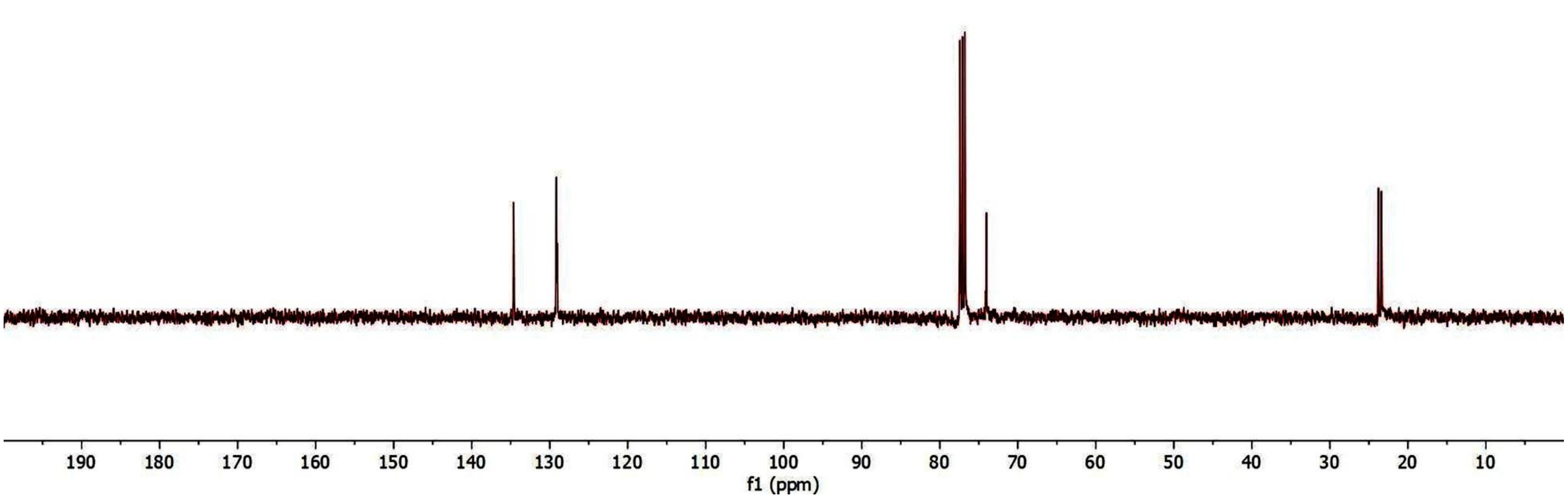

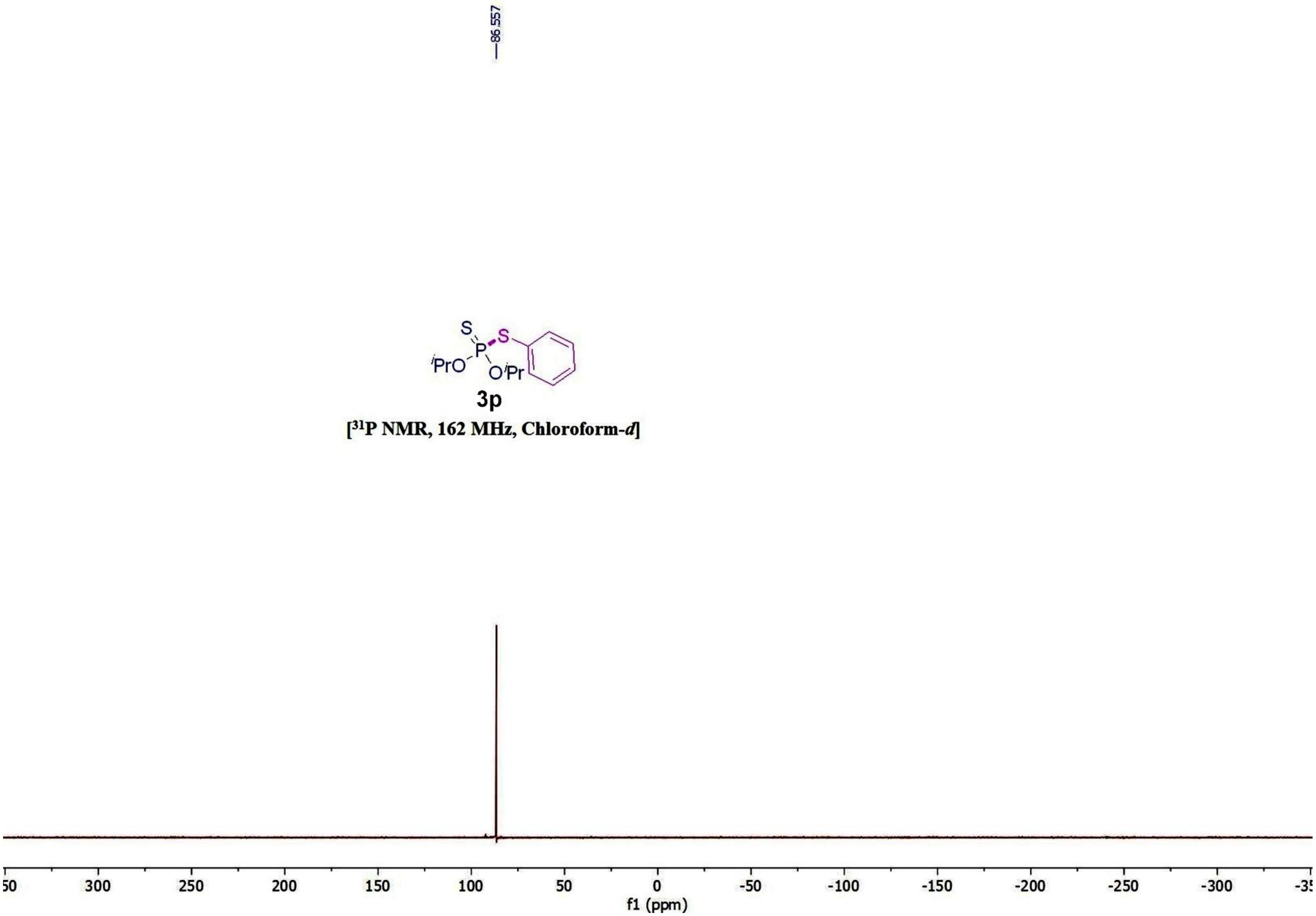

86.557
S
S
P
ⁱPrO
OⁱPr
3p
[31P NMR, 162 MHz, Chloroform-d]
50
300
250
200
150
100
50
0
-50
-100
-150
-200
-250
-300
-35
f1 (ppm)

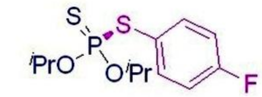

3q

[^{1}H NMR, 400 MHz, Chloroform-*d*]

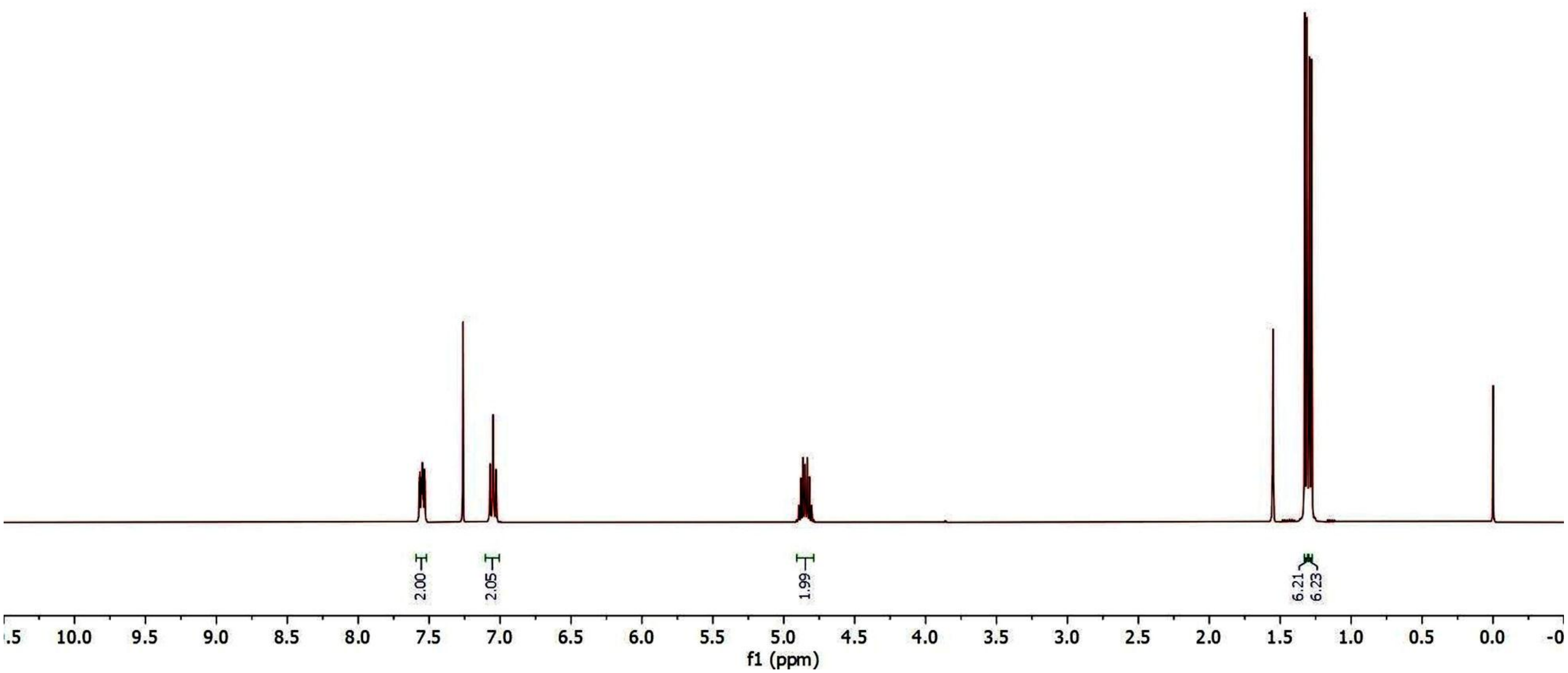

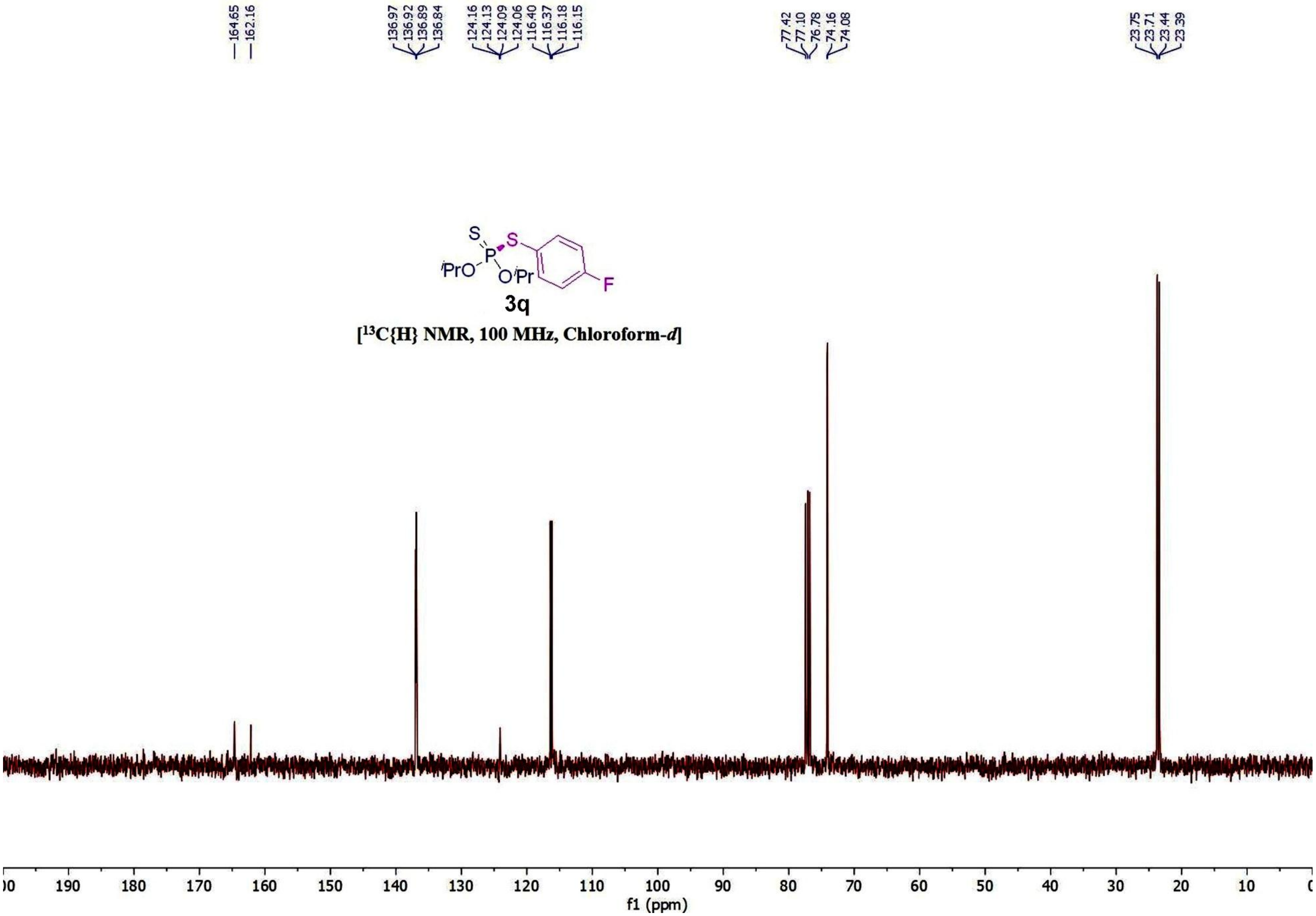
164.65
162.16
136.97
136.92
136.89
136.84
124.16
124.13
124.09
124.06
116.40
116.37
116.18
116.15
77.42
77.10
76.78
74.16
74.08
23.75
23.71
23.44
23.39
S
S
P
iPrO
OiPr
F
3q
[13C{H} NMR, 100 MHz, Chloroform-d]
190
180
170
160
150
140
130
120
110
100
90
80
70
60
50
40
30
20
10
f1 (ppm)

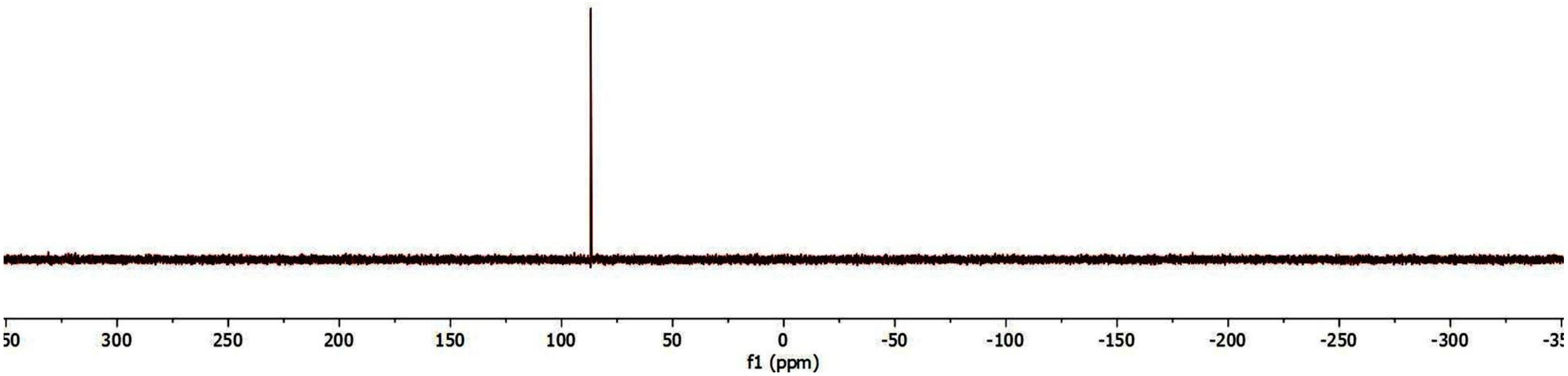

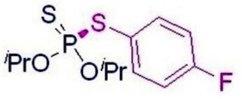

3q

[^{31}P NMR, 162 MHz, Chloroform-*d*]

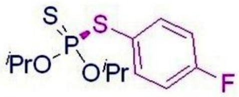

3q

[^{19}F NMR, 376 MHz, Cholroform-*d*]

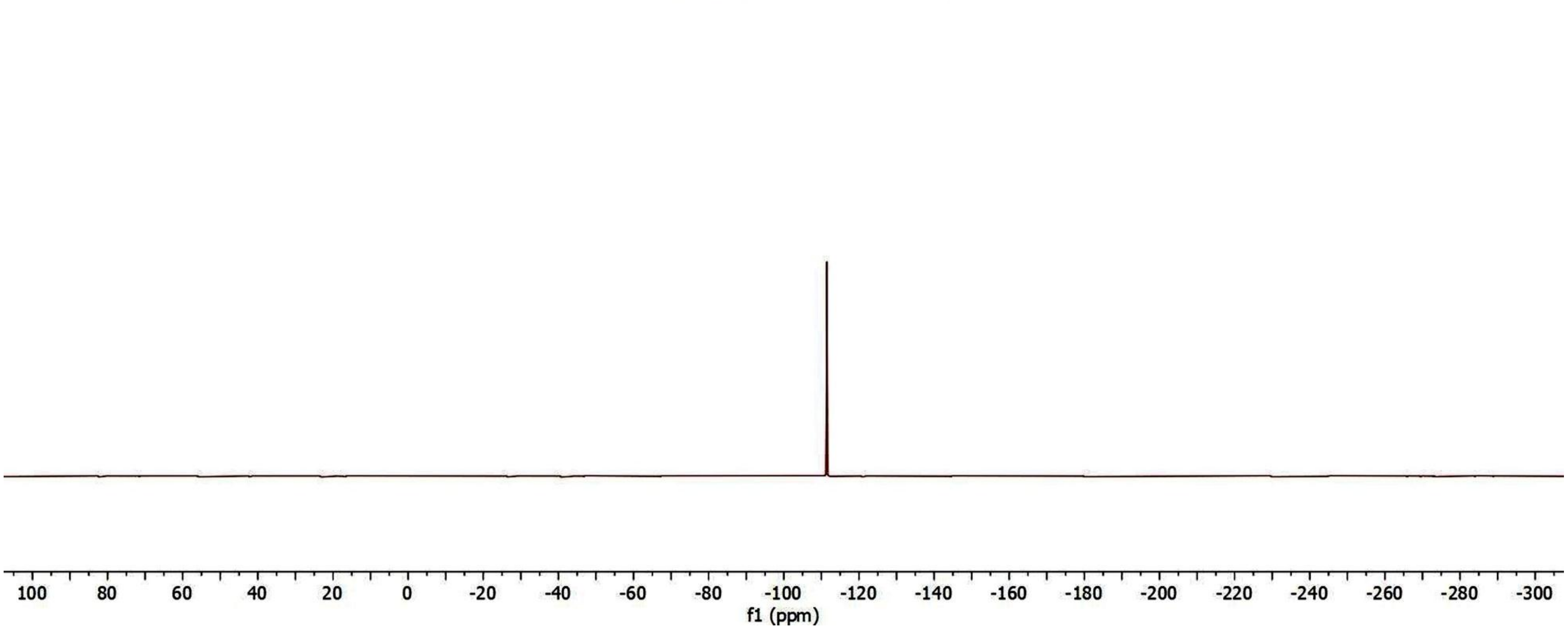

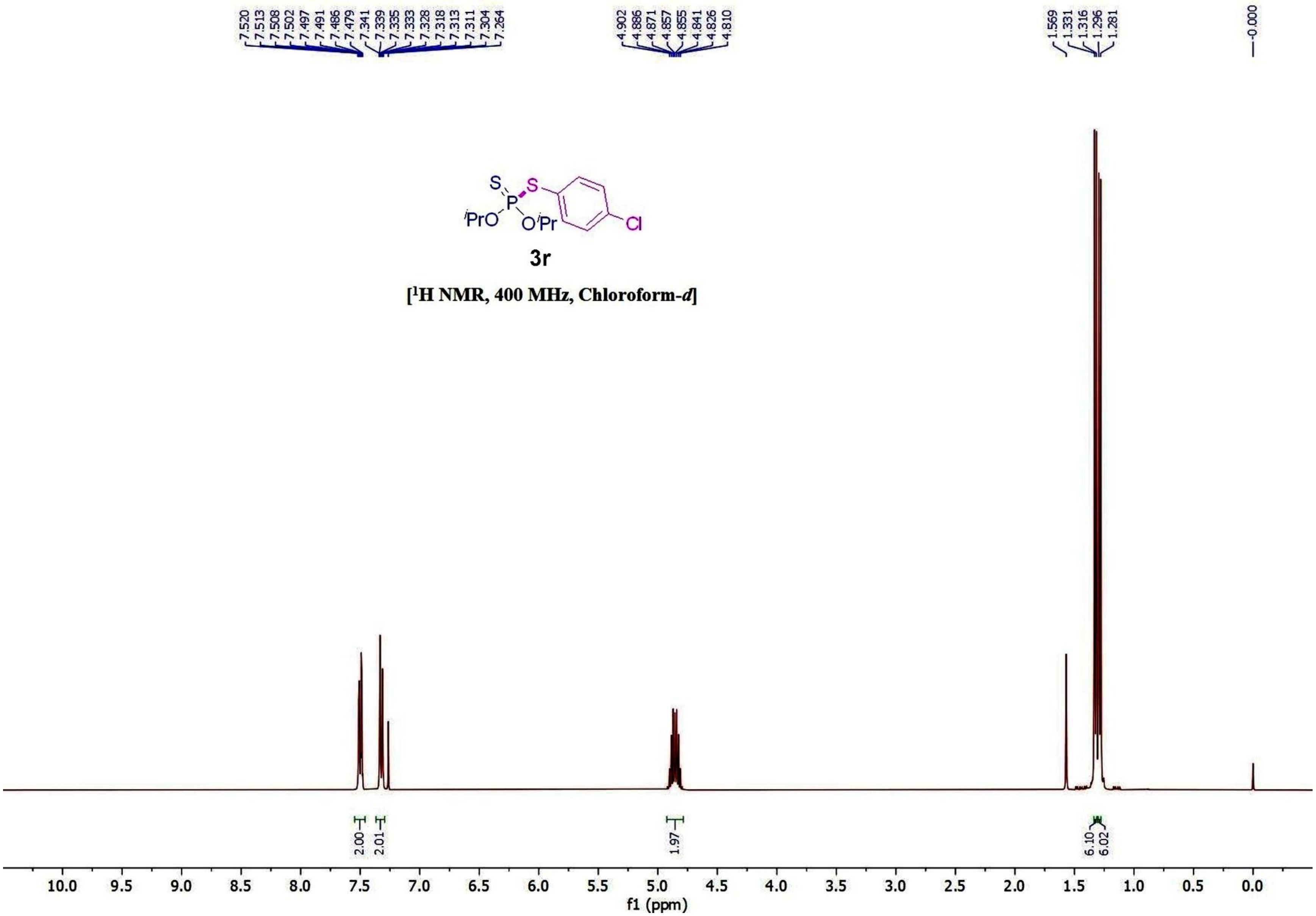

3r

[^{1}H NMR, 400 MHz, Chloroform-*d*]

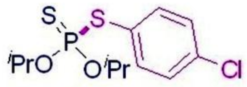

3r

[^{13}C{H} NMR, 100 MHz, Chloroform-*d*]

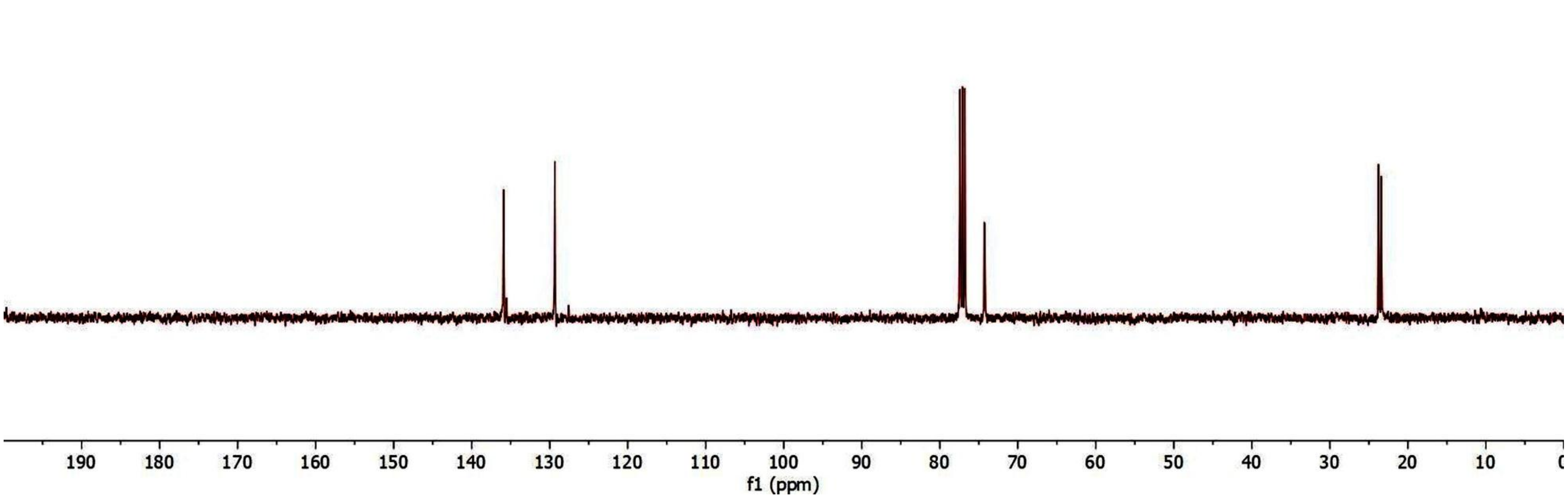

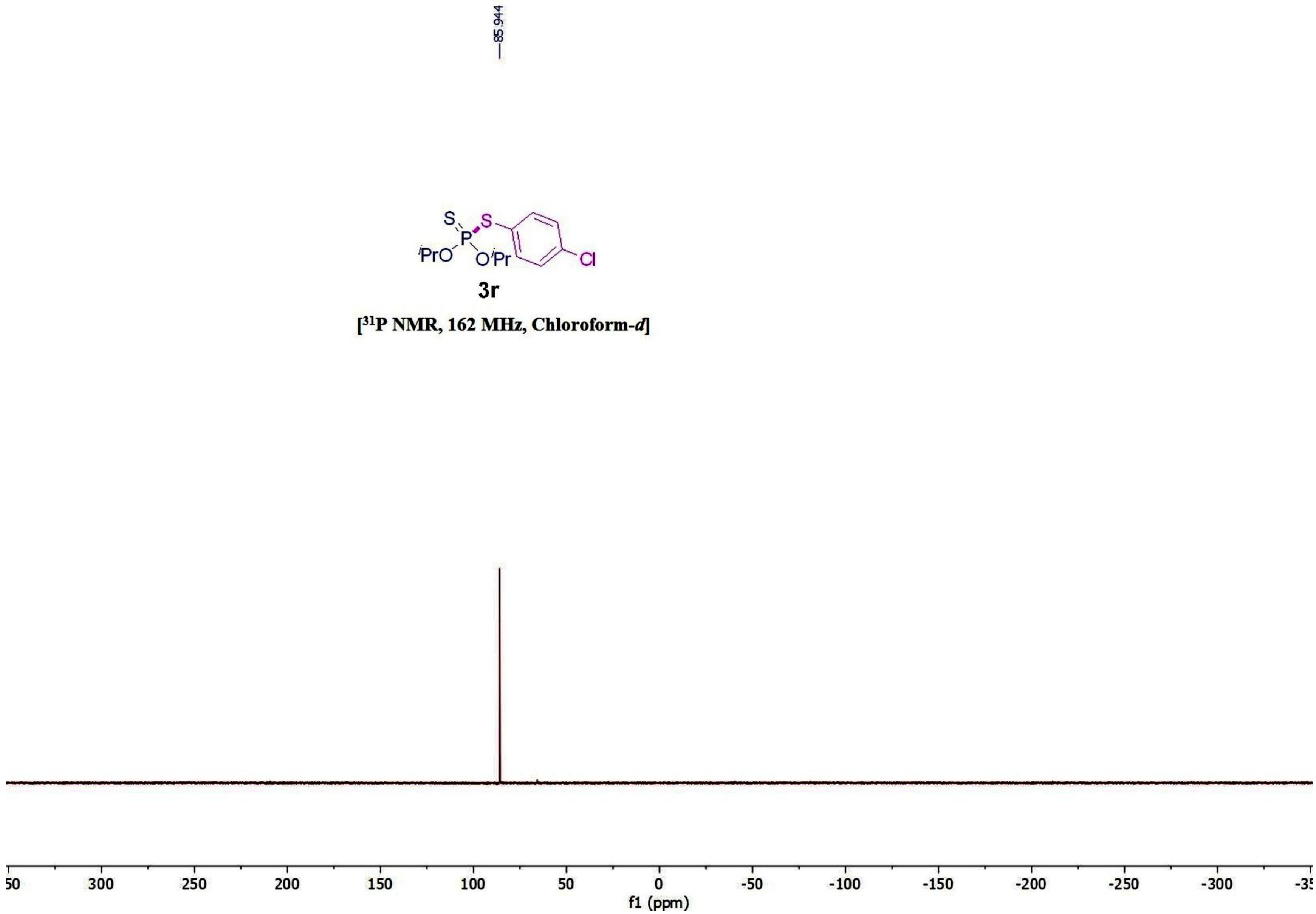
85.944
S
S
P
iPrO
OiPr
Cl
3r
[31P NMR, 162 MHz, Chloroform-d]
50
300
250
200
150
100
50
0
-50
-100
-150
-200
-250
-300
-35
f1 (ppm)

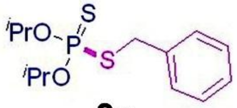

3s

[^{1}H NMR, 400 MHz, Chloroform-*d*]

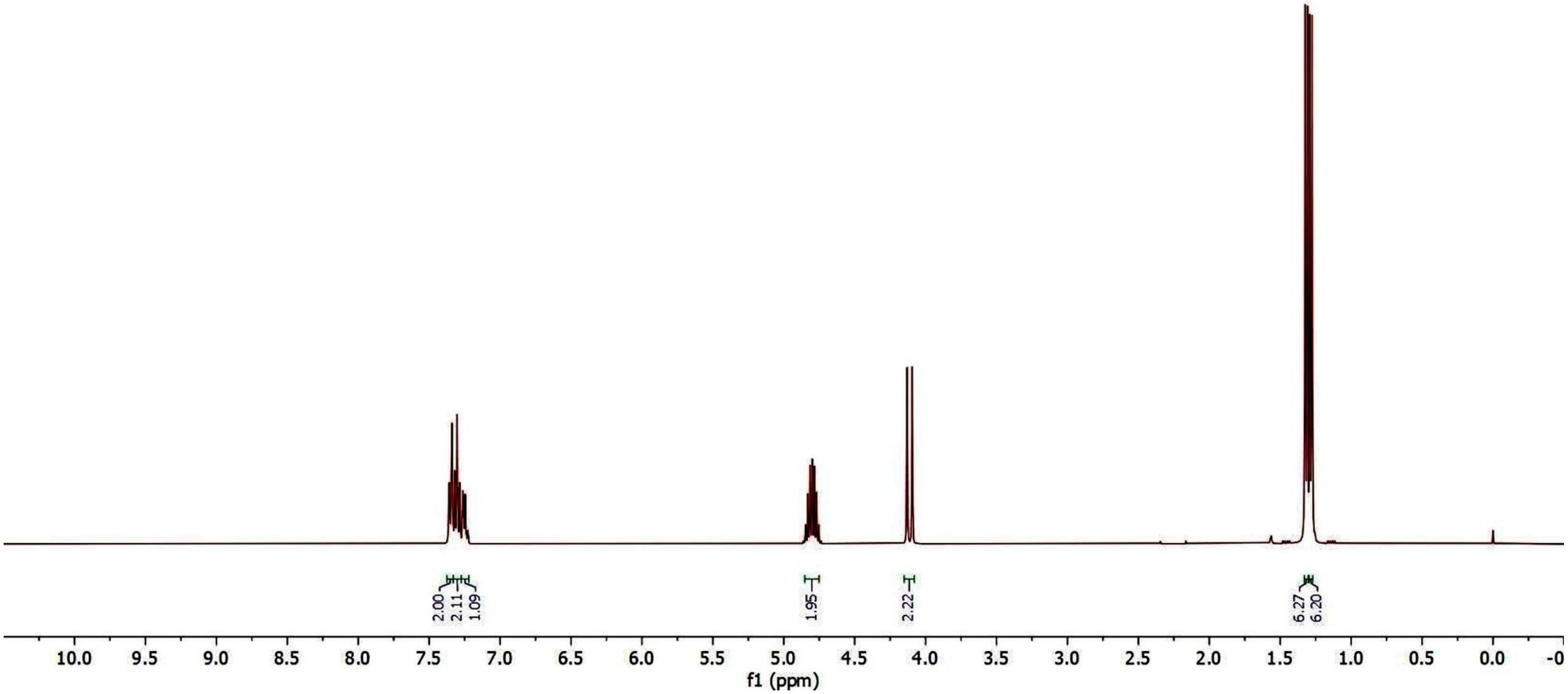

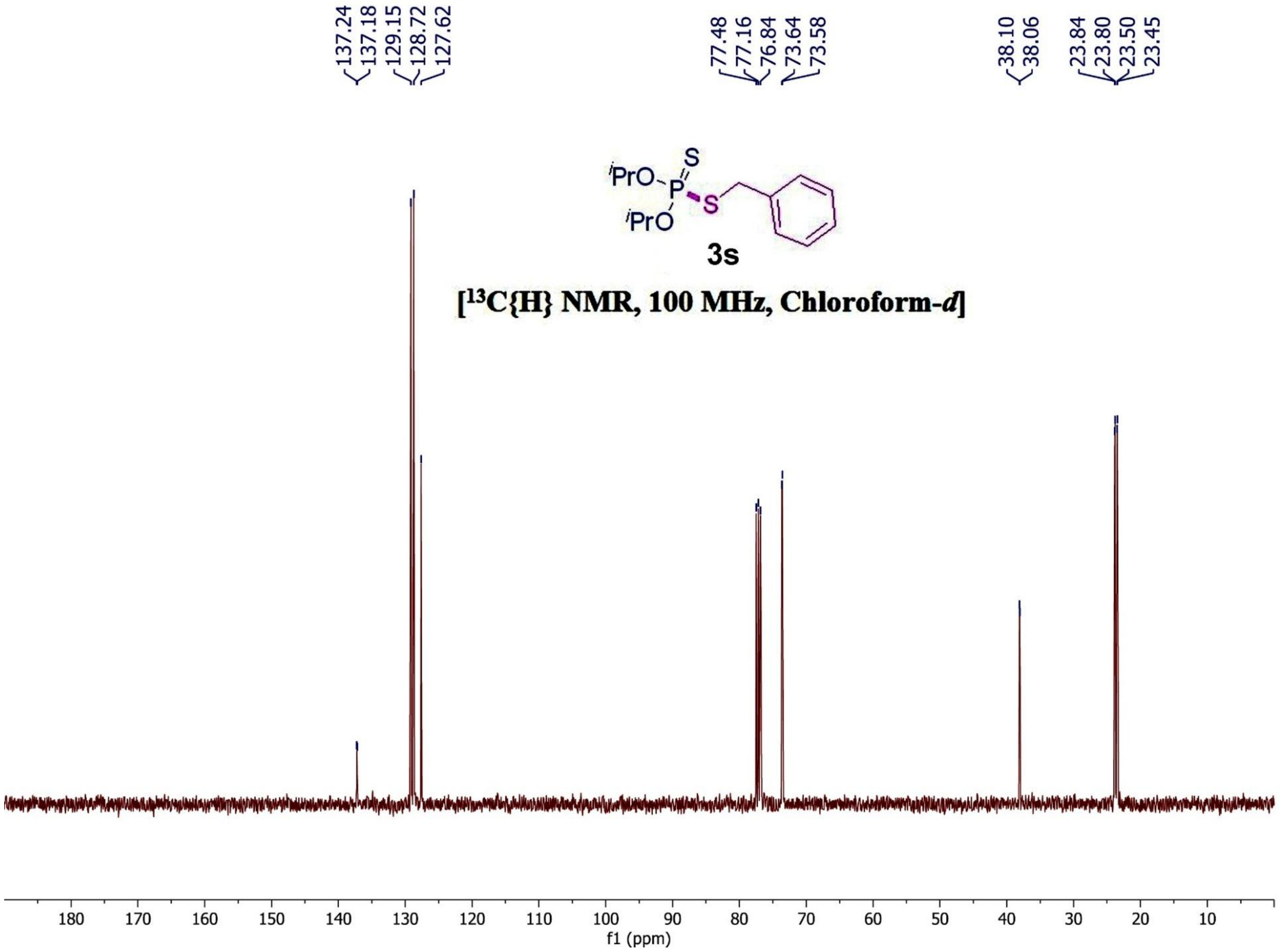

[^{13}C{H} NMR, 100 MHz, Chloroform-*d*]

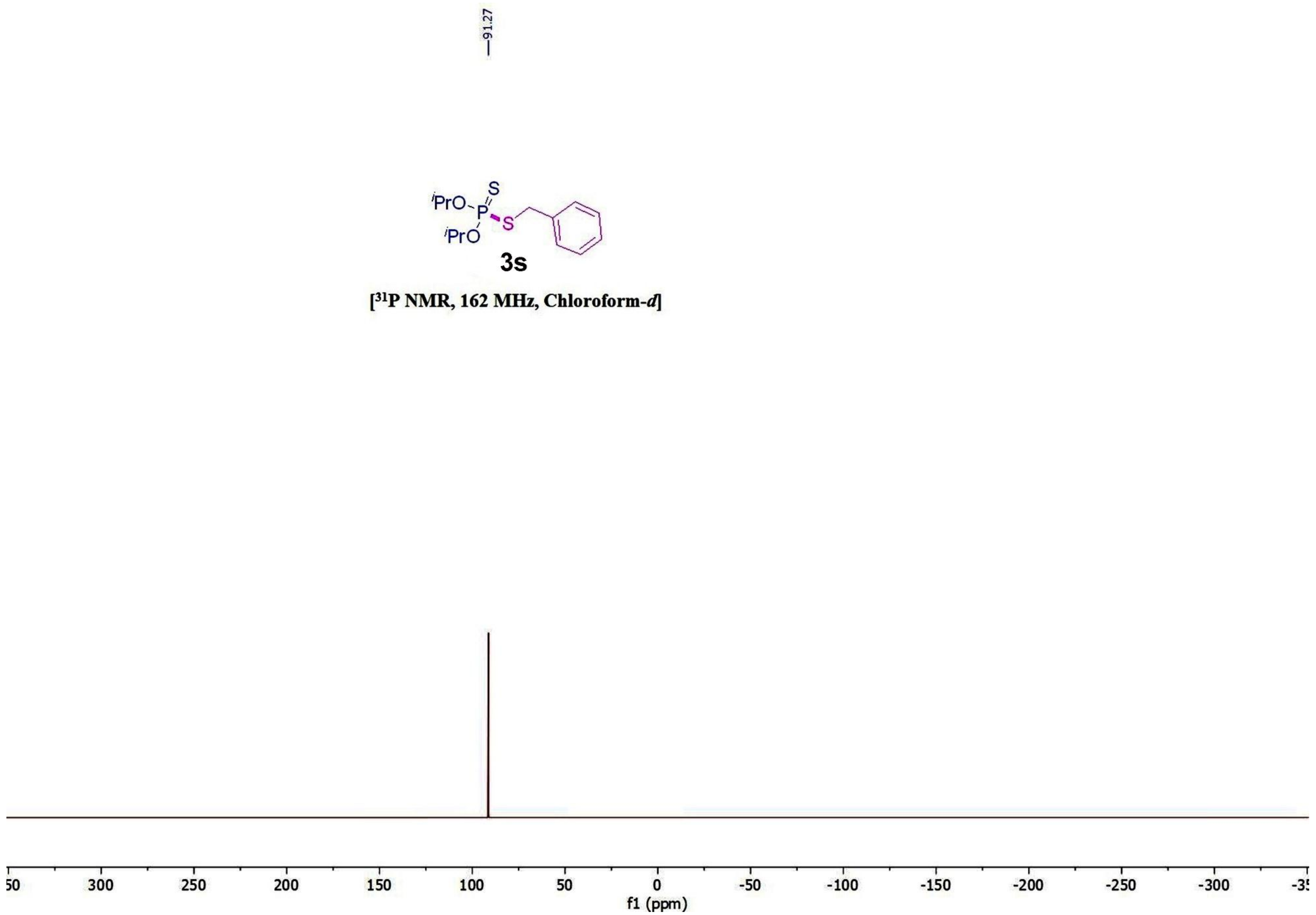

91.27
iPrO
iPrO
P
S
S
3s
[^{31}P NMR, 162 MHz, Chloroform-*d*]
50 300 250 200 150 100 50 0 -50 -100 -150 -200 -250 -300 -35
f1 (ppm)

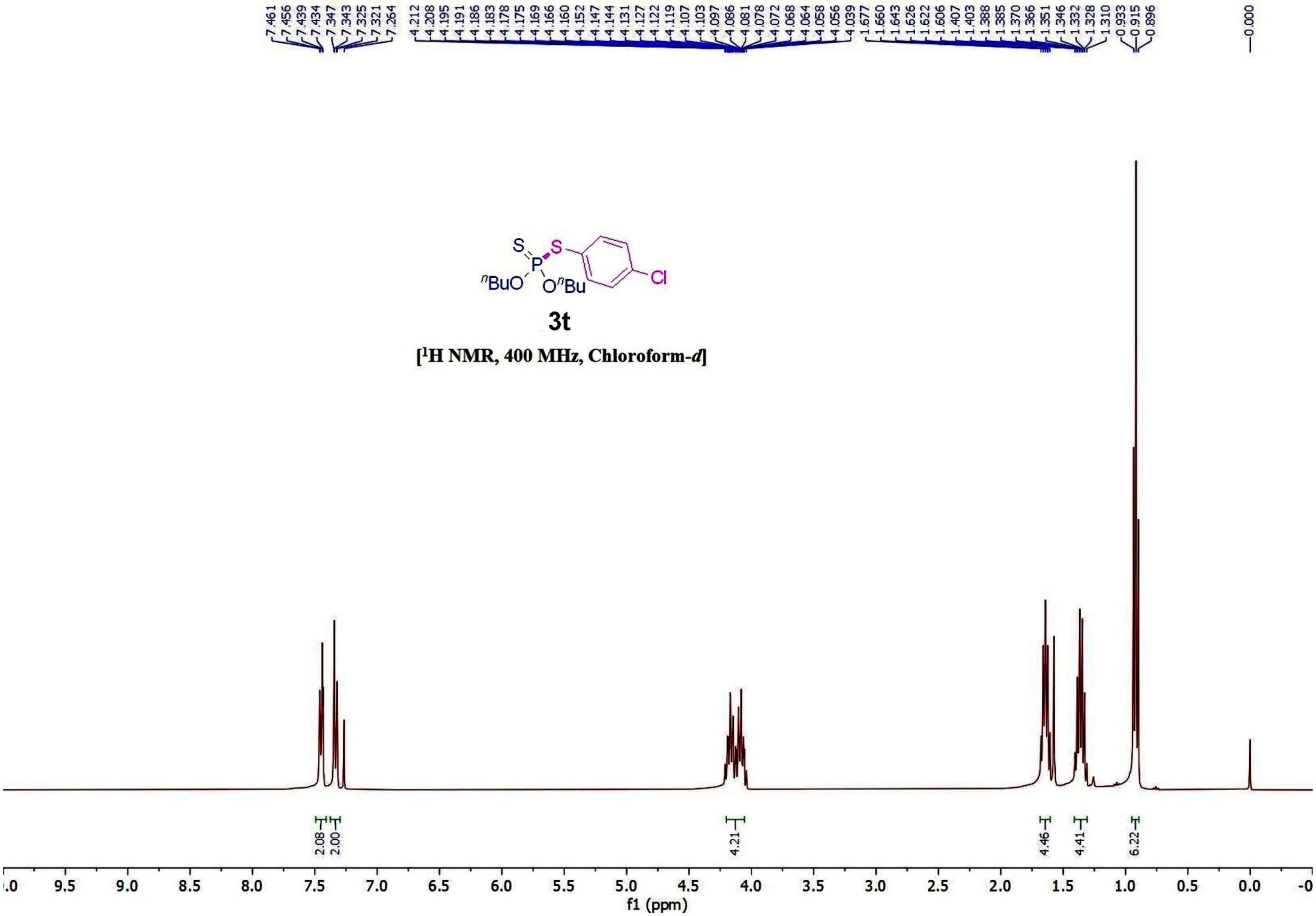

3t

[^{1}H NMR, 400 MHz, Chloroform-*d*]

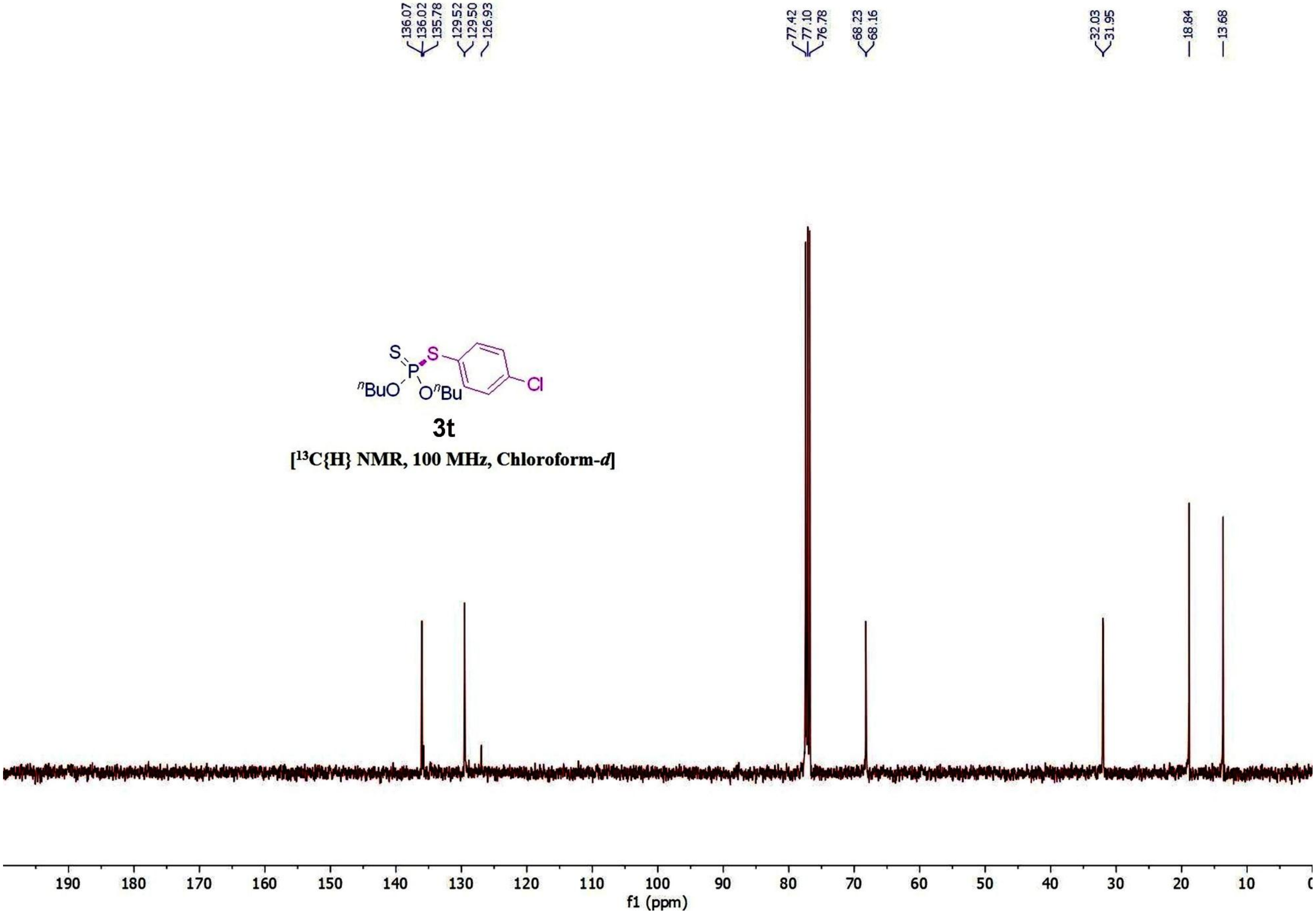

3t

[$^{13}C\{H\}$ NMR, 100 MHz, Chloroform-*d*]

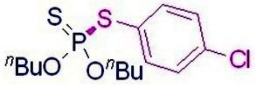

3t

[^{31}P NMR, 162 MHz, Chloroform-*d*]

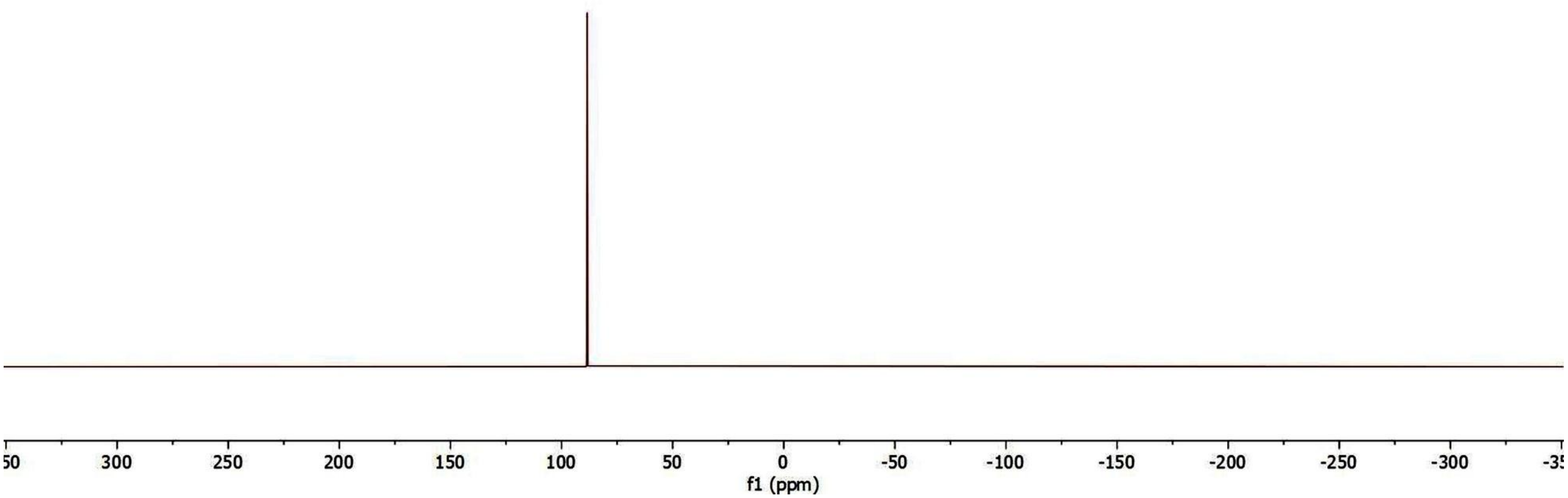

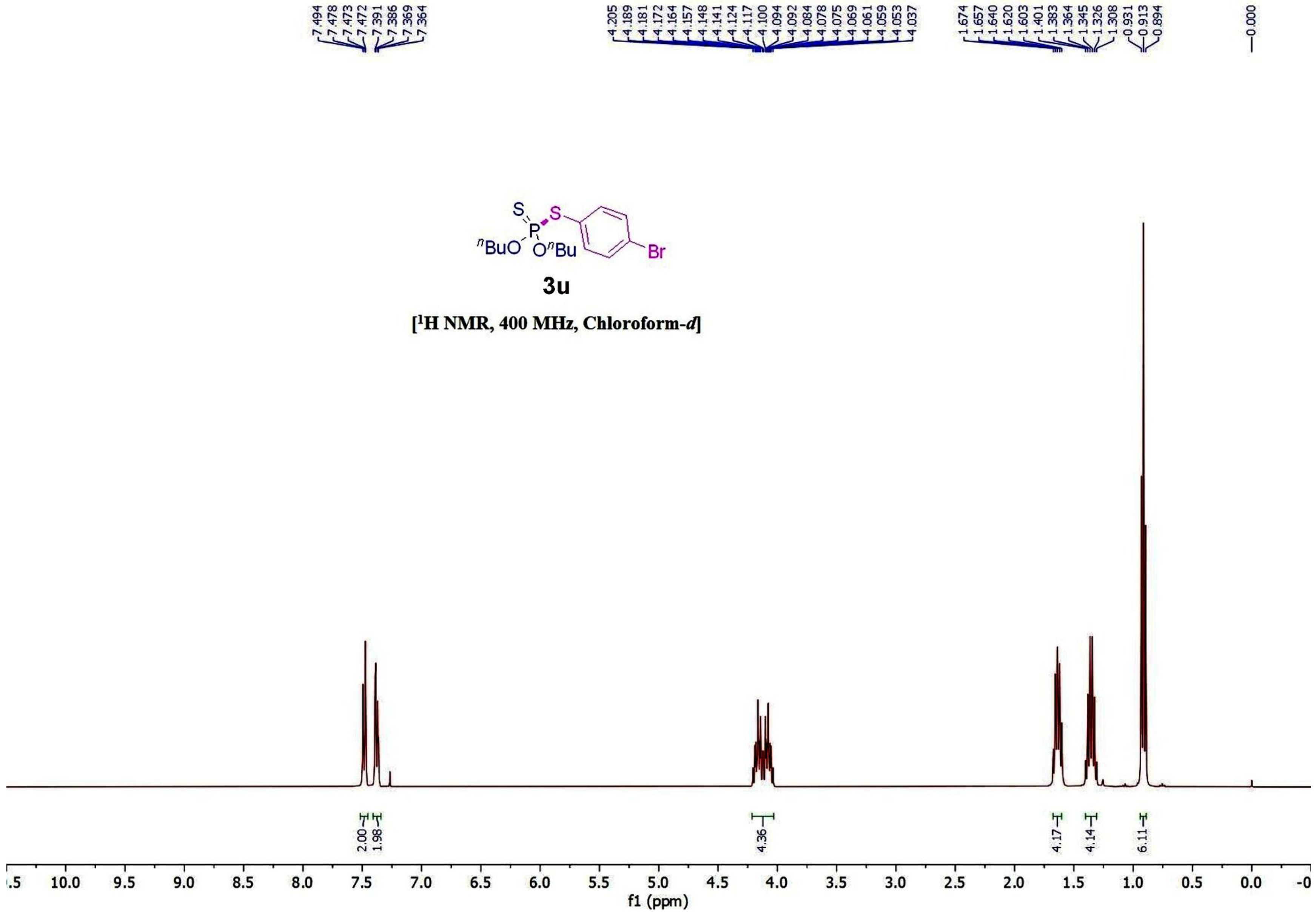

7.494
7.478
7.473
7.472
7.391
7.386
7.369
7.364
4.205
4.189
4.181
4.172
4.164
4.157
4.148
4.141
4.124
4.117
4.100
4.094
4.092
4.084
4.078
4.075
4.069
4.061
4.059
4.053
4.037
1.674
1.657
1.640
1.620
1.603
1.401
1.383
1.364
1.345
1.326
1.308
0.931
0.913
0.894
0.000
S
S
P
ⁿBuO
OⁿBu
Br
3u
[1H NMR, 400 MHz, Chloroform-d]
2.00
1.98
4.36
4.17
4.14
6.11
10.0
9.5
9.0
8.5
8.0
7.5
7.0
6.5
6.0
5.5
5.0
4.5
4.0
3.5
3.0
2.5
2.0
1.5
1.0
0.5
0.0
f1 (ppm)

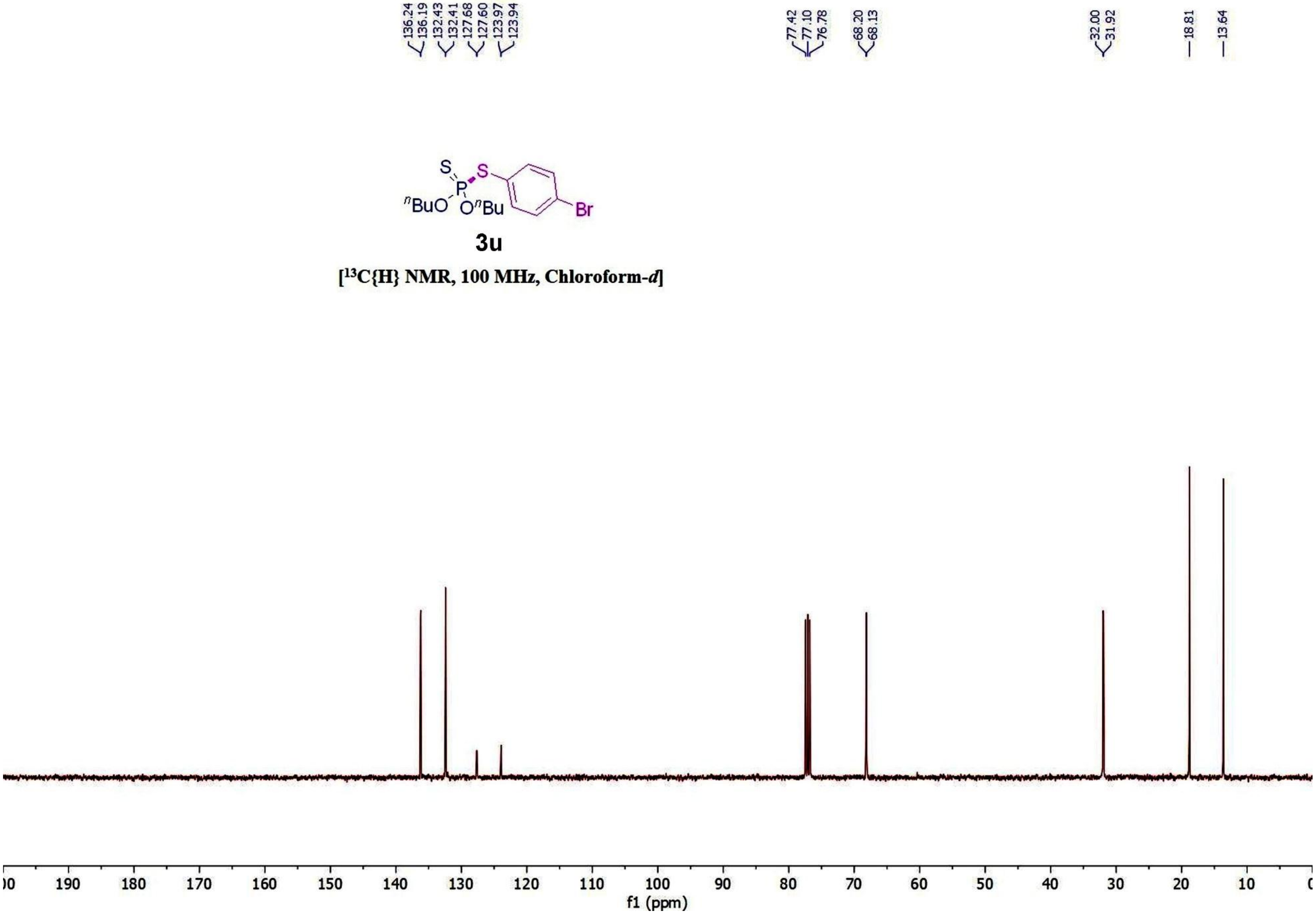

3u

[^{13}C{H} NMR, 100 MHz, Chloroform-*d*]

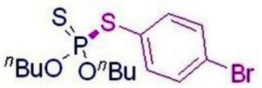

3u

[^{31}P NMR, 162 MHz, Chloroform-*d*]

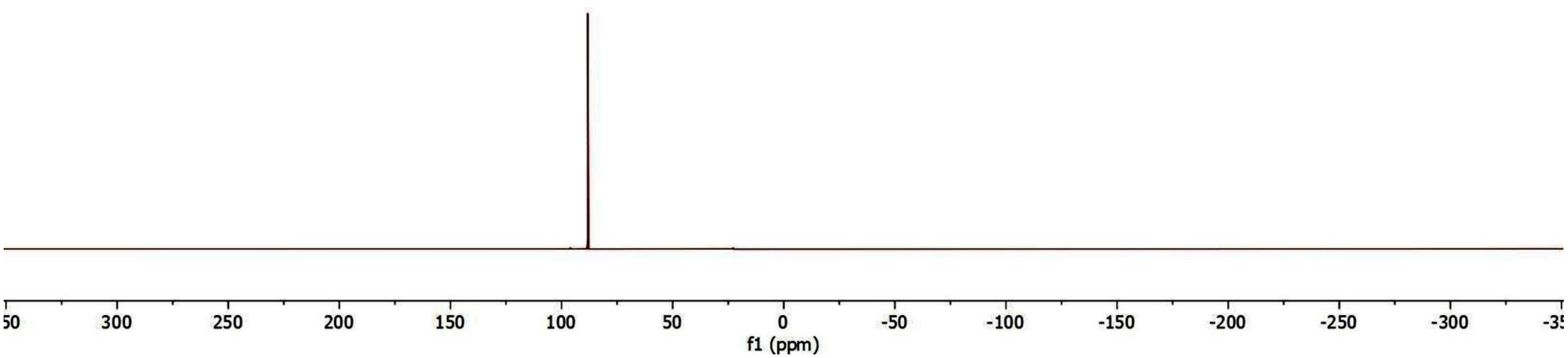

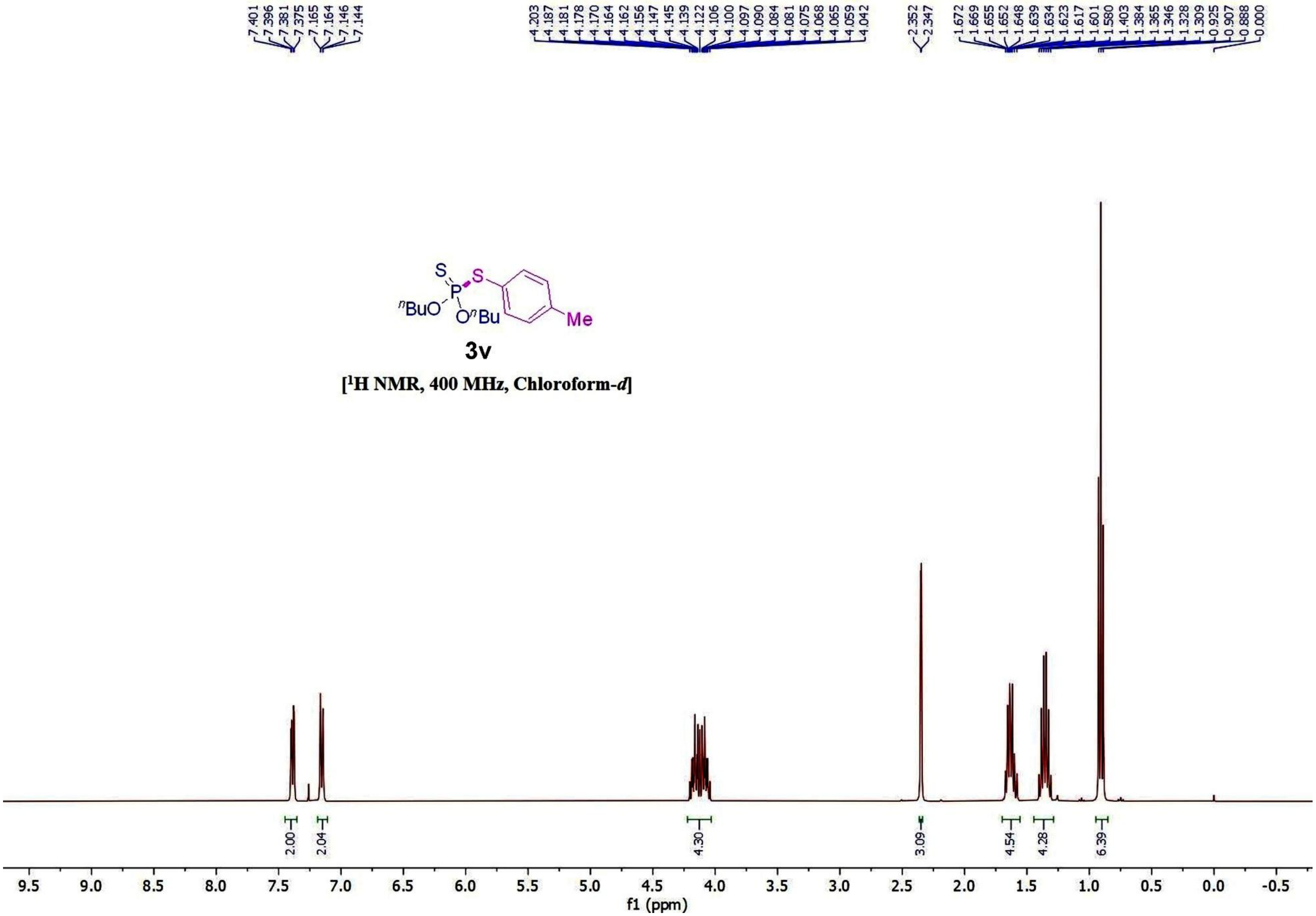
7.401
7.396
7.381
7.375
7.165
7.164
7.146
7.144
4.203
4.187
4.181
4.178
4.170
4.164
4.162
4.156
4.147
4.145
4.139
4.122
4.106
4.100
4.097
4.090
4.084
4.081
4.075
4.068
4.065
4.059
4.042
2.352
2.347
1.672
1.669
1.655
1.652
1.648
1.639
1.634
1.623
1.617
1.601
1.580
1.403
1.384
1.365
1.346
1.328
1.309
0.925
0.907
0.888
0.000
S
S
P
^{n}BuO
O^{n}Bu
Me
3v
[^{1}H NMR, 400 MHz, Chloroform-*d*]
2.00
2.04
4.30
3.09
4.54
4.28
6.39
9.5
9.0
8.5
8.0
7.5
7.0
6.5
6.0
5.5
5.0
4.5
4.0
3.5
3.0
2.5
2.0
1.5
1.0
0.5
0.0
-0.5
f1 (ppm)

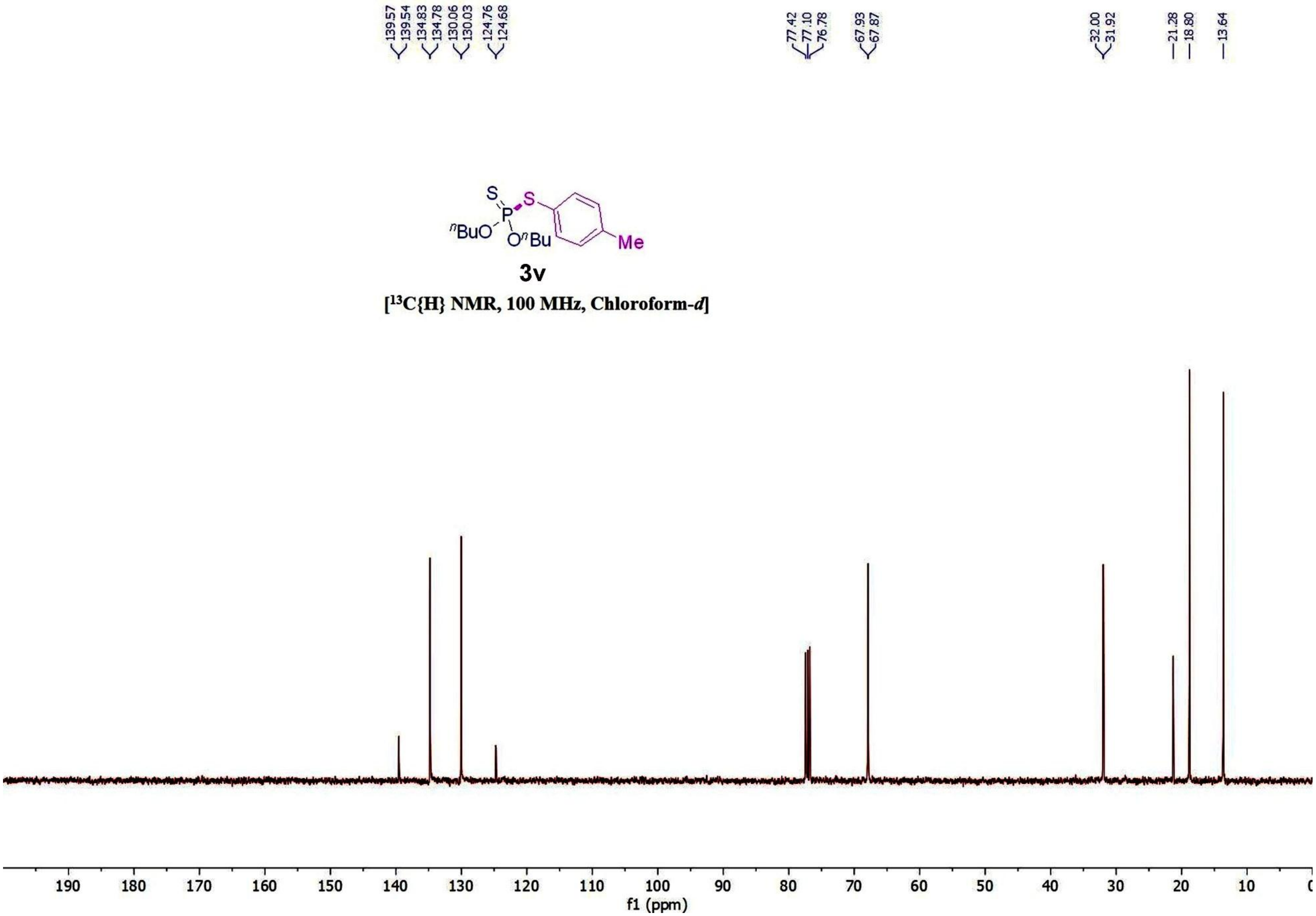

3v

[^{13}C{H} NMR, 100 MHz, Chloroform-*d*]

89.64

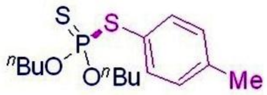

3v

[^{31}P NMR, 162 MHz, Chloroform-*d*]

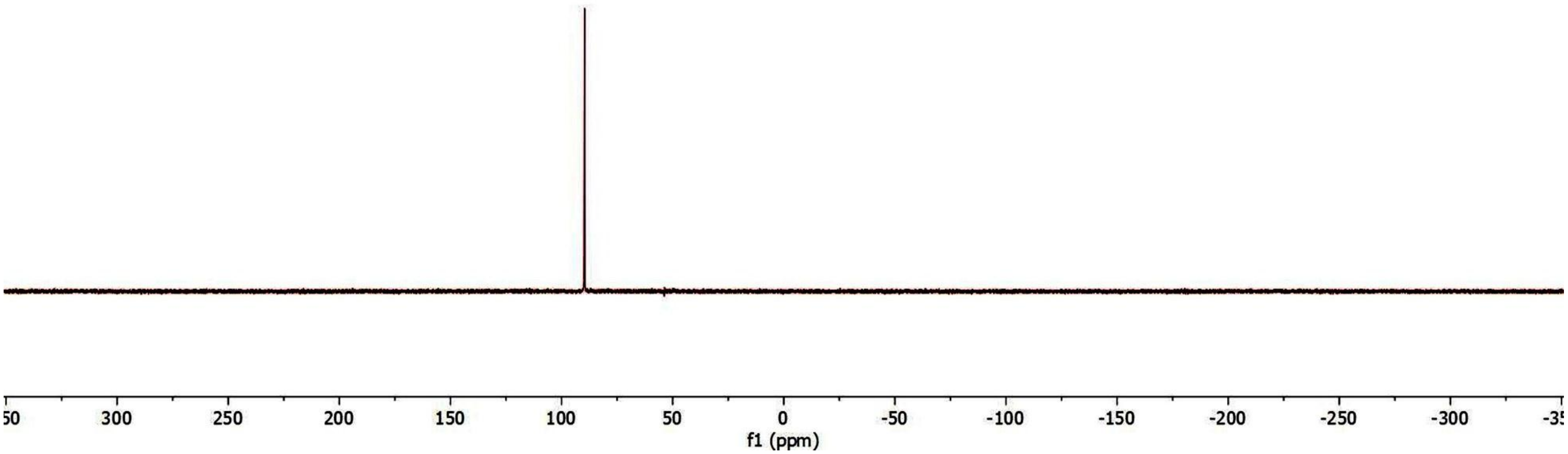

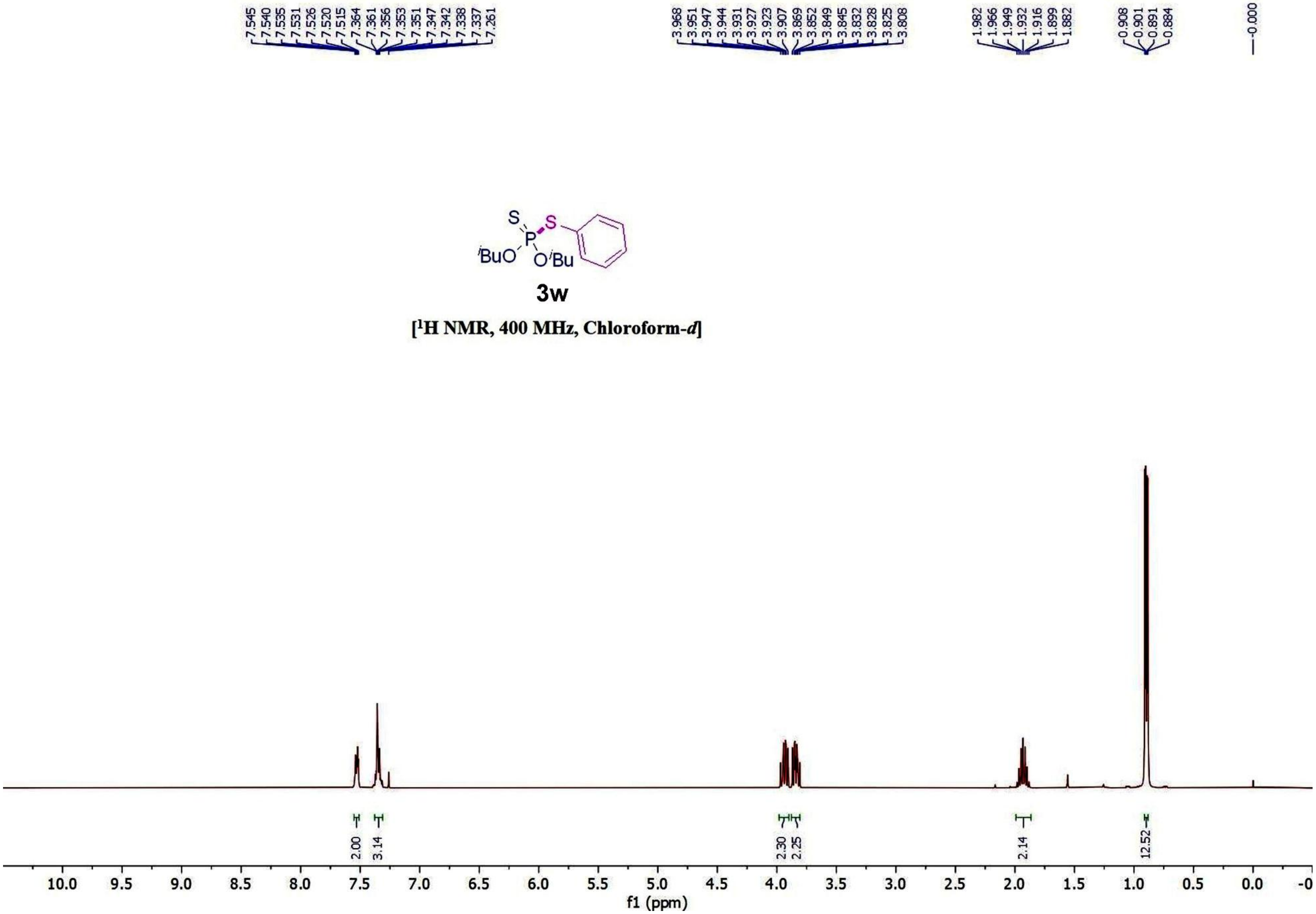

3w

[^{1}H NMR, 400 MHz, Chloroform-*d*]

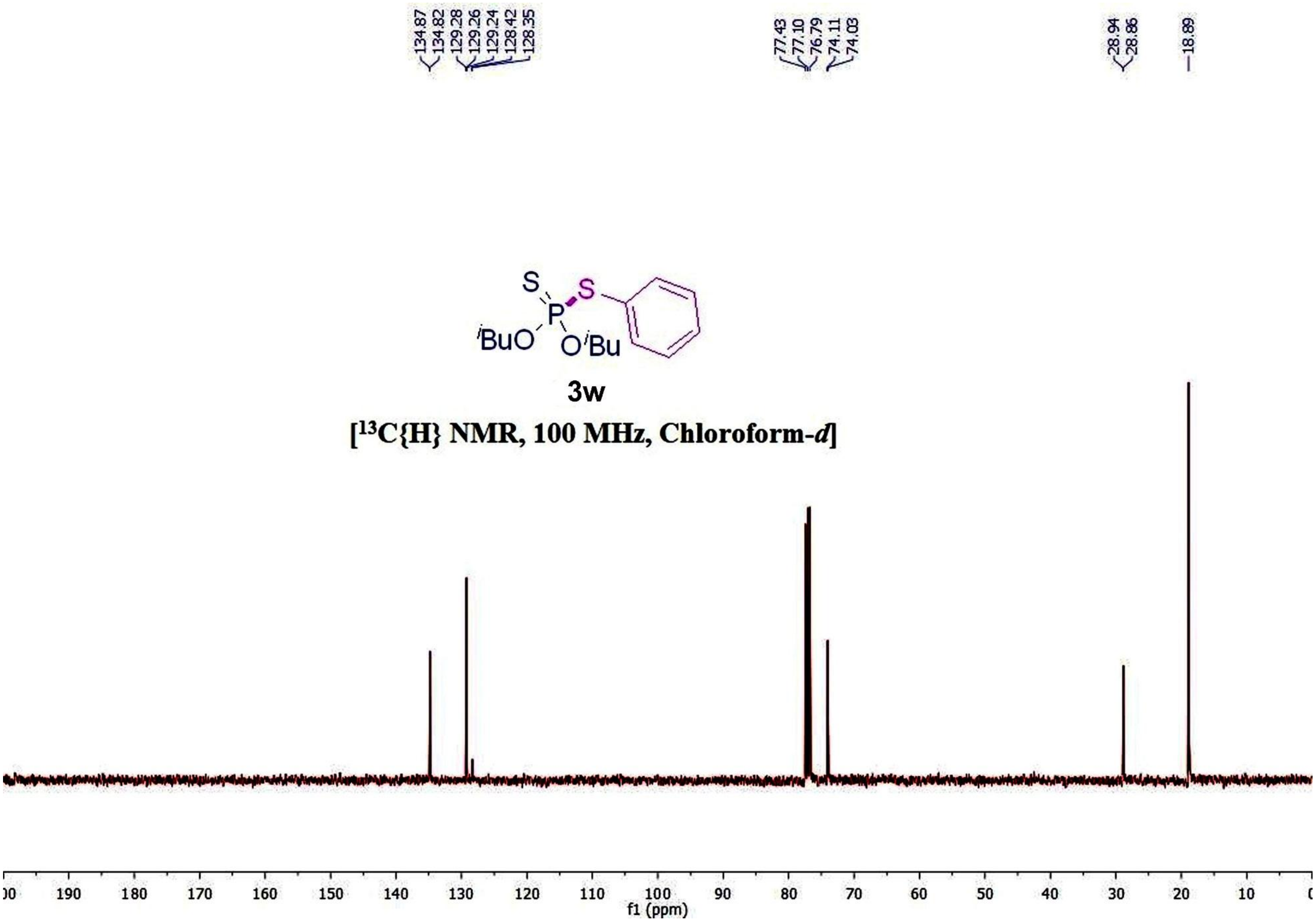

3w

[^{13}C{H} NMR, 100 MHz, Chloroform-*d*]

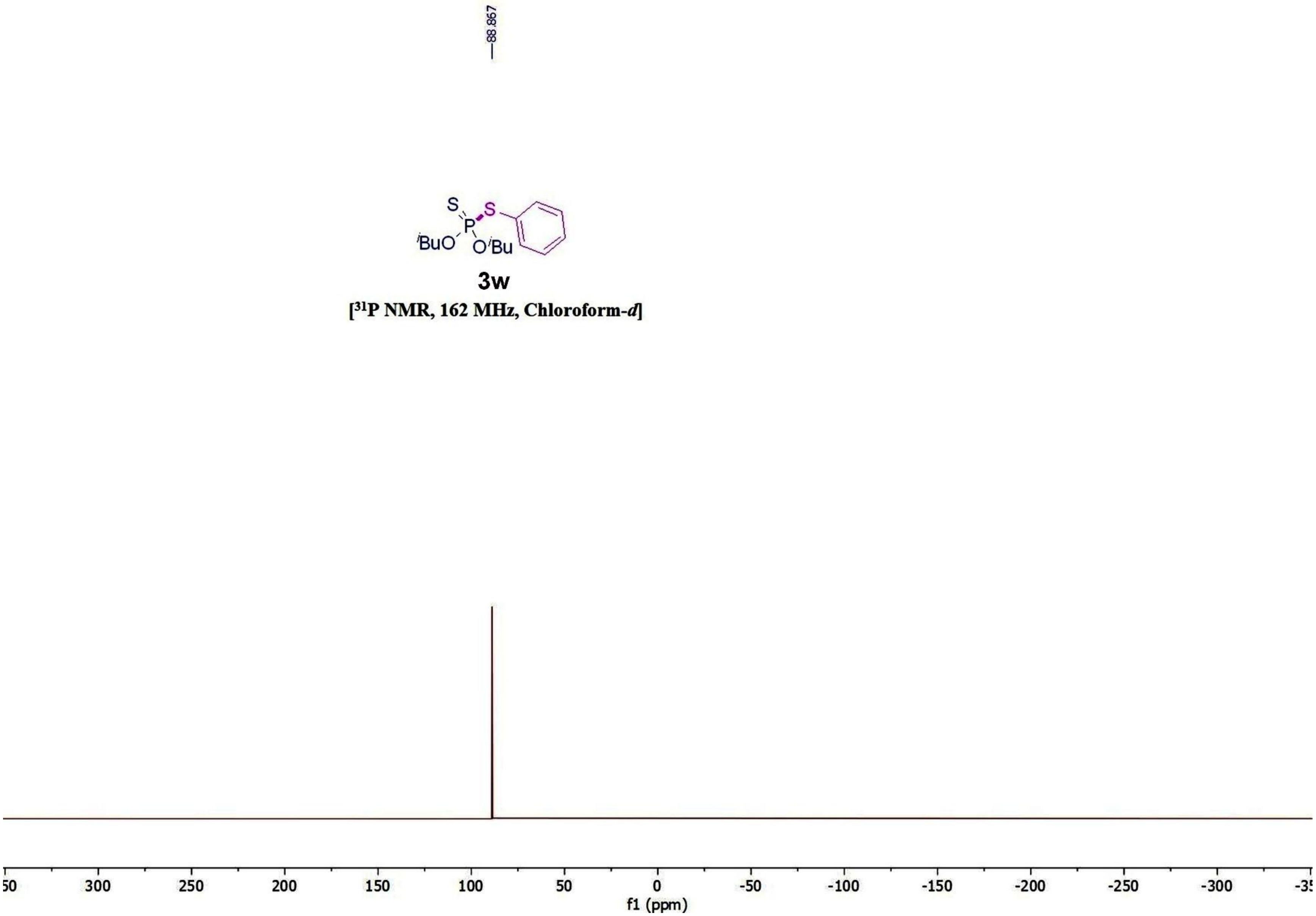
88.867
S
S
P
iBuO
OiBu
3w
[31P NMR, 162 MHz, Chloroform-d]
50
300
250
200
150
100
50
0
-50
-100
-150
-200
-250
-300
f1 (ppm)

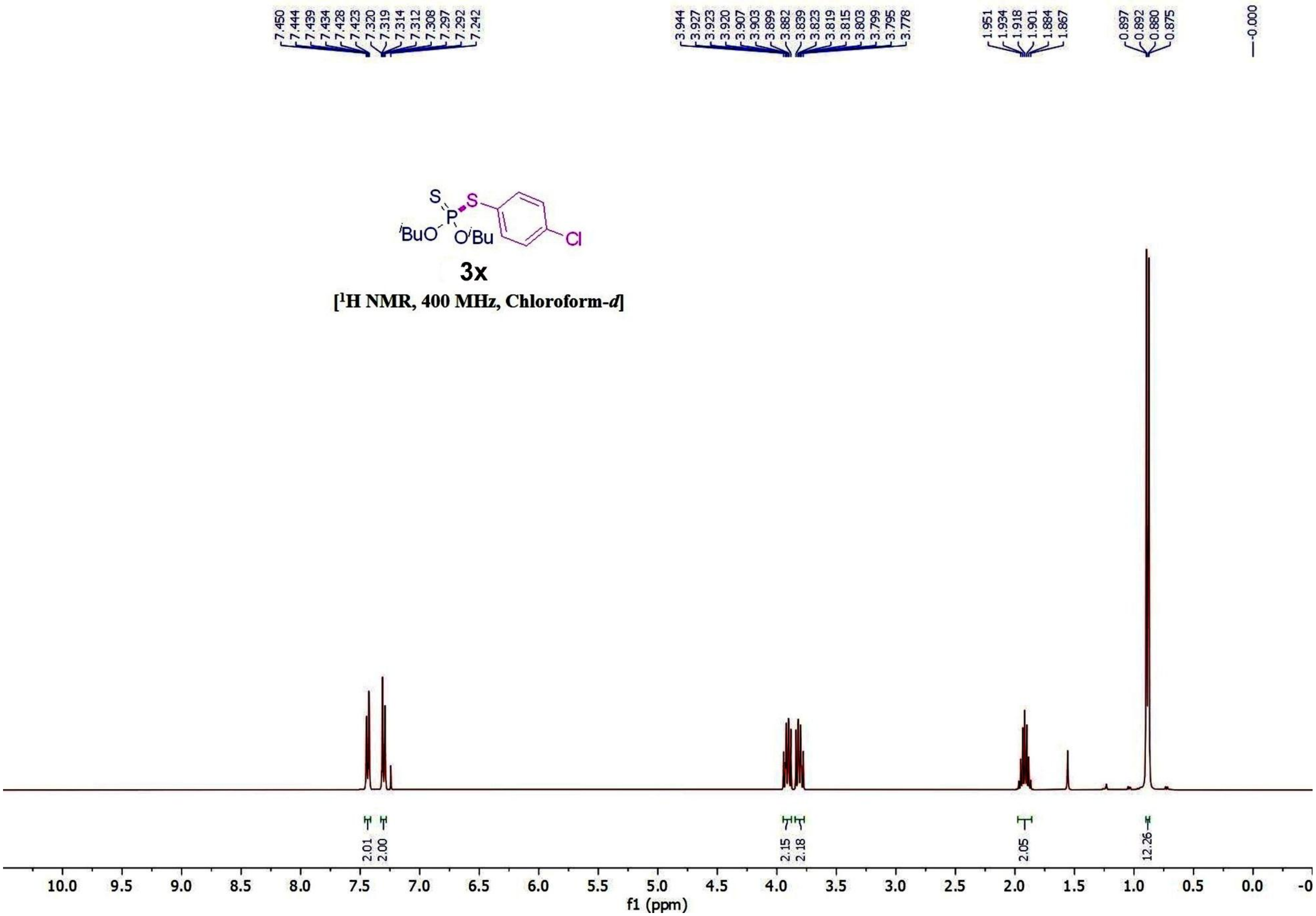
S
S
P
iBuO
OiBu
Cl
3x
[1H NMR, 400 MHz, Chloroform-d]
7.450 7.444 7.439 7.434 7.428 7.423 7.320 7.319 7.314 7.312 7.308 7.297 7.292 7.242
3.944 3.927 3.923 3.920 3.907 3.903 3.899 3.882 3.839 3.823 3.819 3.815 3.803 3.799 3.795 3.778
1.951 1.934 1.918 1.901 1.884 1.867
0.897 0.892 0.880 0.875
-0.000
2.01 2.00 2.15 2.18 2.05 12.26
f1 (ppm)

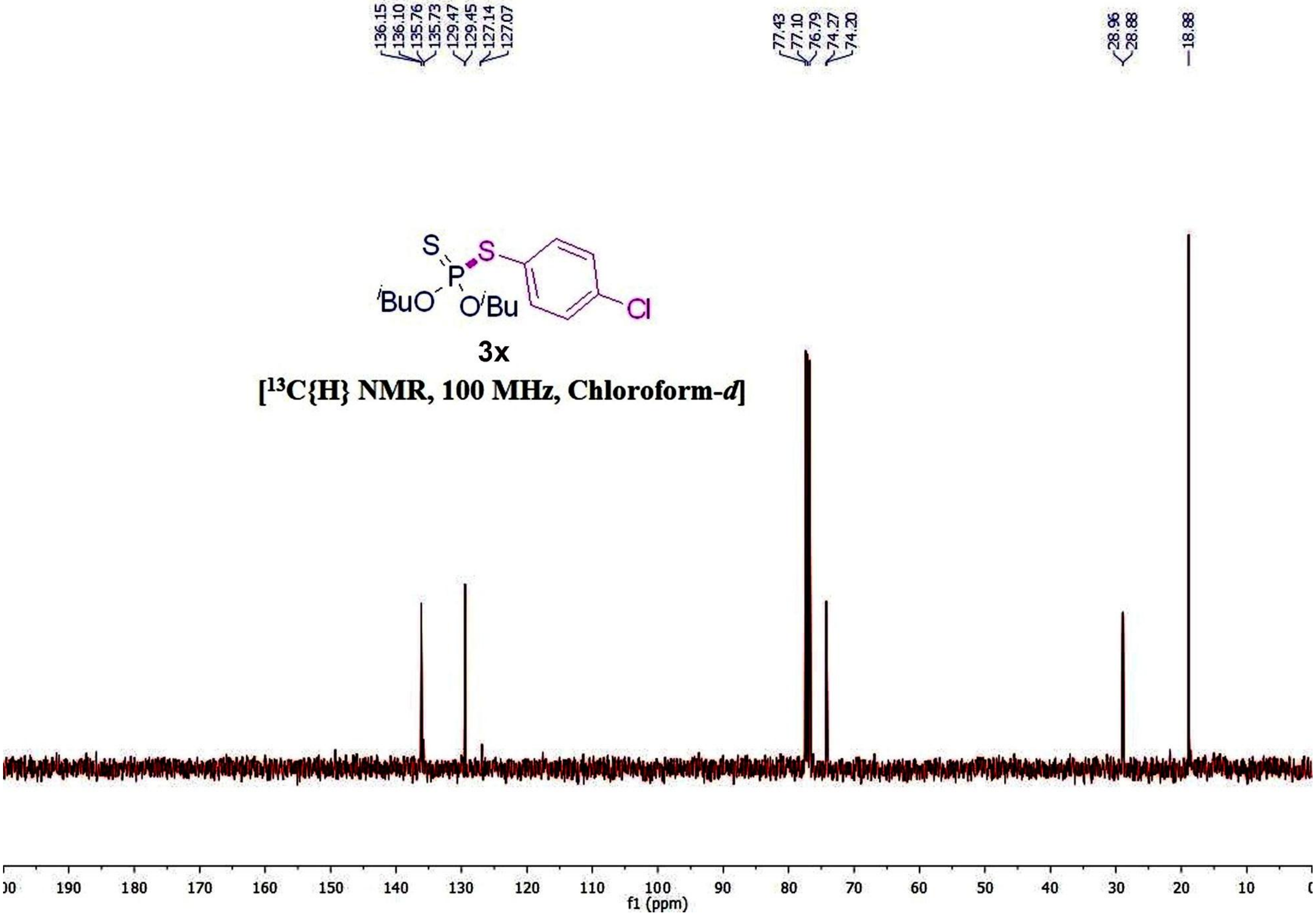

3x

[^{13}C{H} NMR, 100 MHz, Chloroform-*d*]

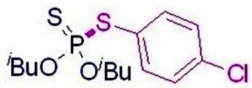

3x

[^{31}P NMR, 162 MHz, Chloroform-*d*]

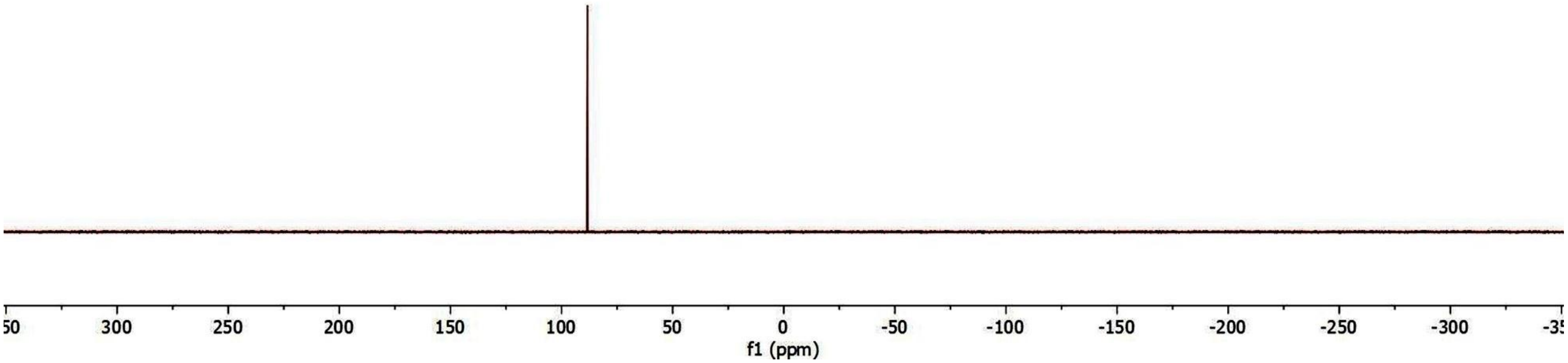

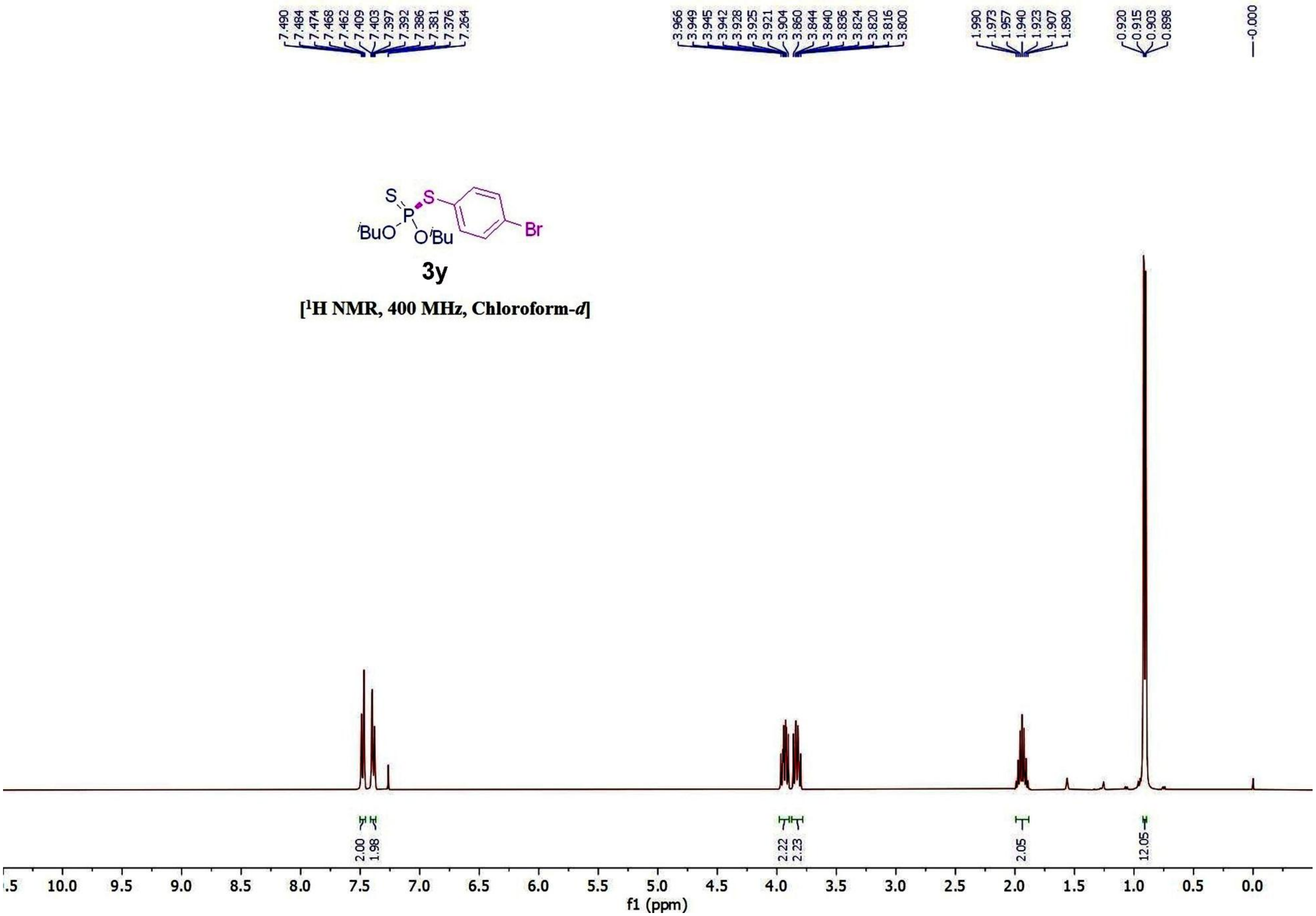

3y

[^{1}H NMR, 400 MHz, Chloroform-*d*]

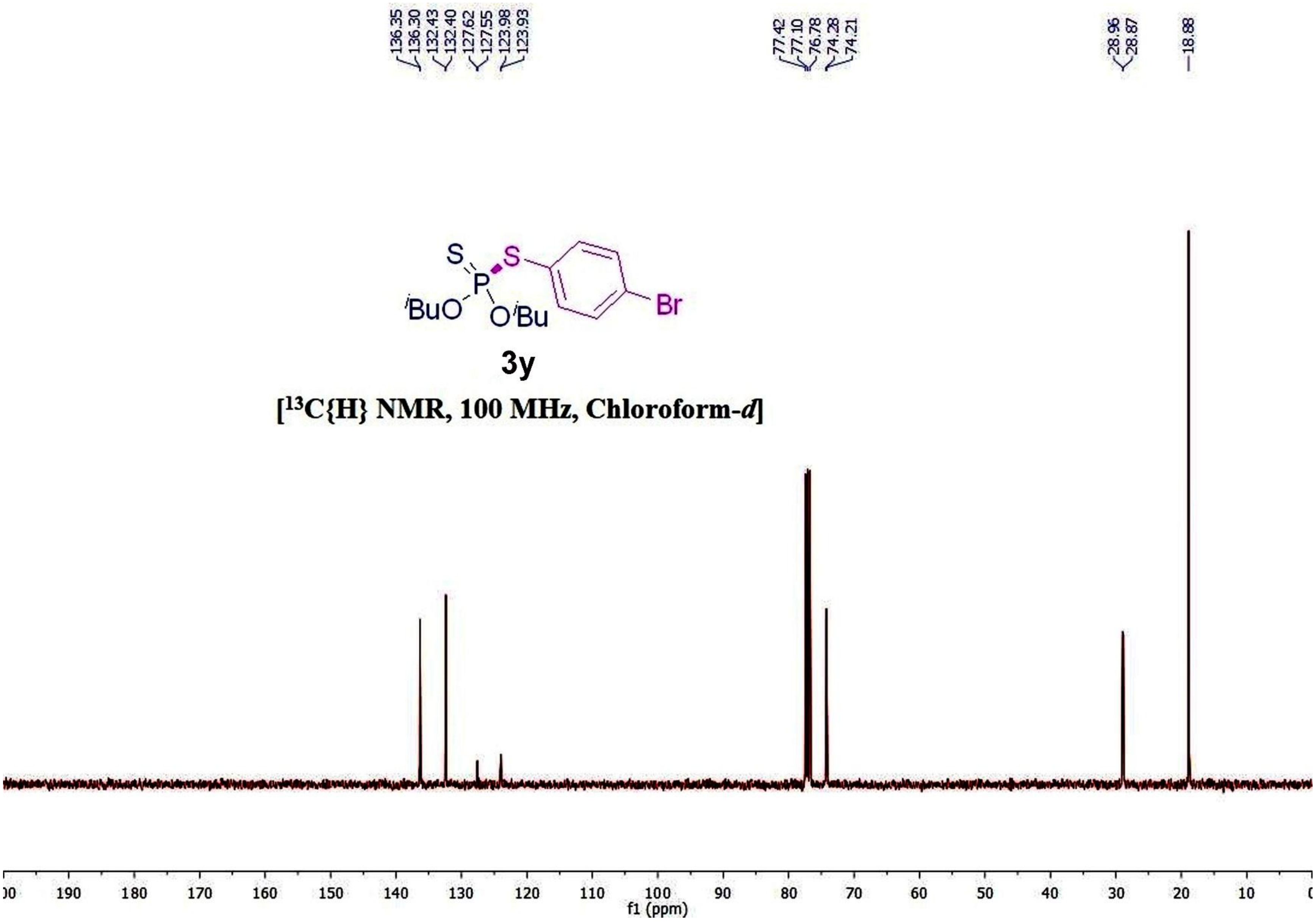
136.35
136.30
132.43
132.40
127.62
127.55
123.98
123.93
77.42
77.10
76.78
74.28
74.21
28.96
28.87
18.88
S
S
P
iBuO
O
iBu
Br
3y
[13C{H} NMR, 100 MHz, Chloroform-d]
190
180
170
160
150
140
130
120
110
100
90
80
70
60
50
40
30
20
10
f1 (ppm)

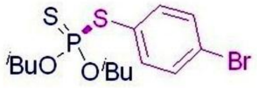

3y

[^{31}P NMR, 162 MHz, Chloroform-*d*]

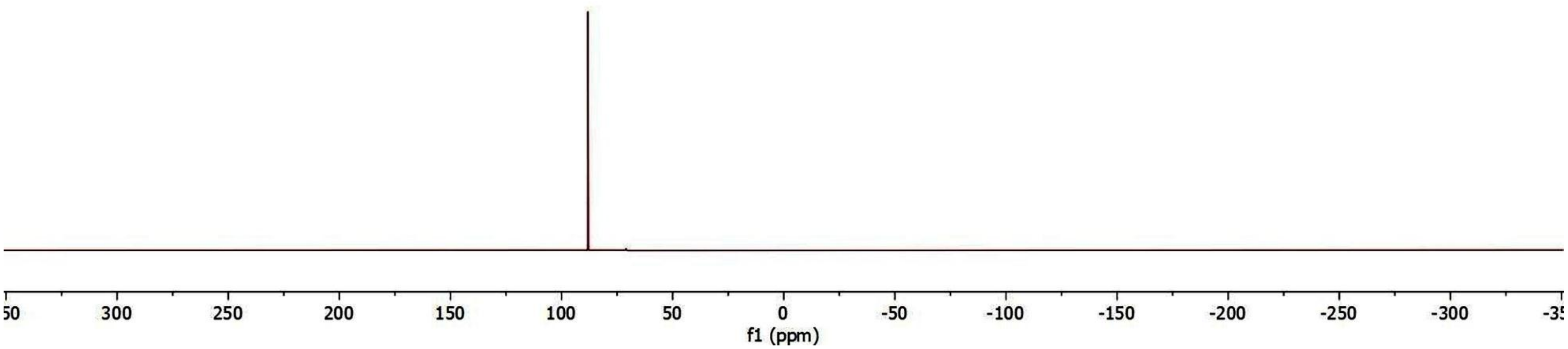

6. Calculation of E-factors for all the synthesized compounds (3a-3y)

The following formulae were used for calculating E-factor.[6] The Calculated data for compounds **3a-3y** are presented in Table S1.

Documentation of Calculations of Green Metric (E-factor) for One Representative Entry, *viz.* *O*,*O*-diethyl *S*-phenyl phosphorodithioate (3a):

$$\text{E-factor} = \frac{\text{Mass of wastes}}{\text{Mass of product}} = \frac{\text{Mass of raw materials} - \text{Mass of product}}{\text{Mass of product}}$$

$$= \frac{(0.0.77\text{ g} + 0.0.66\text{ g} + 0.016\text{ g}) - 0.125\text{ g}}{0.125\text{ g}} = 0.29\ \ (\text{g/g})$$

Table S1. Calculated E-factors for the compounds (**3a-3y**)

**Sl. No**	**Product**	**E-Factor** (g/g)	**Sl. No**	**Product**	**E-Factor** (g/g)
1	**3a**	0.29	14	**3n**	1.02
2	**3b**	0.69	15	**3o**	1.83
3	**3c**	0.41	16	**3p**	0.26
4	**3d**	1.00	17	**3q**	0.35
5	**3e**	1.33	18	**3r**	0.24
6	**3f**	0.72	19	**3s**	1.32
7	**3g**	0.41	20	**3t**	0.58
8	**3h**	0.85	21	**3u**	1.19
9	**3i**	1.13	22	**3v**	1.05
10	**3j**	0.56	23	**3w**	0.48
11	**3k**	0.91	24	**3x**	0.48
12	**3l**	0.77	25	**3y**	0.55
13	**3m**	0.85			

[#]Lower is the value, greener is the process

7. References

(1) B. R. Shen, P. Annamalai, S. F. Wang, R. Bai and C.-F. Lee, *J. Org. Chem*., 2022, **87**, 8858.

(2) S. Kovacs, B. Bayarmagnai, A. Aillerie and L. J. Gooßen, *Adv. Synth. Catal.,* 2018, **360**, 1913.

(3) Mel'nikow et al. *Zhurnal Obshchei Khimii*, 1959, **29**, 3291, 3292, 3294.

(4) B. Kaboudin and H. Norouzi, *Synthesis*, 2004, 2035.

(5) Mel'nikow et al. *Zhurnal Obshchei Khimii,* 1959, **29**, 1612.

(6) (a) R. A. Sheldon, *ACS Sustainable Chem. Eng.*, 2018, **6**, 32. (b) G. Brahmachari, I karmakar and P. Karmakar, *Green Chem*., 2021, **23**, 4762.
